# A New Comprehensive Generic Framework for *Tettigometra* Latreille, 1804 *s.l.*: A Taxonomic and Nomenclatural Revision of the Tribe Tettigometrini (Hemiptera: Fulgoromorpha)

**DOI:** 10.3390/insects17010030

**Published:** 2025-12-24

**Authors:** Fariba Mozaffarian, Thierry Bourgoin

**Affiliations:** 1 Insect Taxonomy Research Department, Iranian Research Institute of Plant Protection, Agricultural Research, Education and Extension Organization, Tehran P.O. Box 1454-19395, Iran; mozaffarian@iripp.ir; 2 Institut de Systématique, Évolution, Biodiversité, ISYEB-UMR 7205 MNHN-CNRS-Sorbonne, Université-EPHE-University Antilles, Muséum National d’Histoire Naturelle, CP 50, 57 rue Cuvier, 75005 Paris, France

**Keywords:** tettigometridae, informal taxonomic group, new genera, identification key to genera, comparative morphology, male genitalia

## Abstract

The taxonomy of *Tettigometra* Latreille, 1804 *s.l.* (Hemiptera, Tettigometridae, Tettigometrini) has long remained unstable due to weak diagnostic characters, conflicting interpretations, and frequent misidentifications. Based on extensive examination of the illustrated literature and major museum collections, a new generic framework is proposed, relying primarily on comparative analyses of male genital morphology (particularly the mediodorsal aedeagal process and paired ventral anal processes) while external morphology alone is shown to be unreliable for stable delimitation. In discussing the need for a taxonomy that is both phylogenetically grounded and operational, allowing reliable identifications, preserving compatibility with past determinations despite the lack of molecular evidence, and remaining flexible enough to accommodate future results, we recognize two informal taxonomic groups and fourteen genera. The tettigometrinan group includes *Tettigometra*, *Brachyceps*, *Metroplaca*, *Mimarada*, *Mediodentometra* ***gen. nov.***, and *Persiametra* ***gen. nov.***; the apexometrinan group includes *Apexometra* ***gen. nov.***, *Erratometra* ***gen. nov.***, *Eurychila*, *Hystrigonia*, *Micracanthometra* ***gen. nov.***, *Mitricephalus*, *Stirometra*, and *Macrometrina*. Following a conservative and operational taxonomic approach, we refrained from describing new species or accepting unsubstantiated synonymies, preferring to retain potentially distinct forms as provisionally valid species pending molecular confirmation. Each taxon is listed with its taxonomic and nomenclatural status, diagnosis, species composition, and distribution. Problematic taxa and misapplied names are clarified, and a key to genera is provided.

## Simple Summary

The taxonomy of the traditionally broad genus *Tettigometra* Latreille, 1804, *s.l*. has long been problematic, largely due to the lack of clear diagnostic characters, conflicting identification keys, and the accumulation of inconsistent species assignments and synonymies. Based on an extensive comparative analysis of male genital morphology from both historical literature and museum collections, this study redefines the generic framework of the *Tettigometrini* into fourteen distinct valid genera, five of which are newly described. The genera are diagnosed primarily on male genital structures and are arranged into two informal taxonomic groups, following a conservative and operational taxonomic approach that is discussed. Accordingly, also several undescribed or misinterpreted forms that may represent new species are addressed, although we refrained from formally describing new species or accepting unsubstantiated synonymies, preferring instead to retain potentially distinct forms as provisionally valid species pending confirmation through future molecular data. We believe that this approach ensures greater nomenclatural stability while preserving the flexibility required for future integrative revisions. While this new morphological framework provides a robust basis for species identification and clarifies long-standing ambiguities, it still requires molecular confirmation. Nevertheless, it establishes a coherent foundation for future molecular, ecological, biogeographic, and phylogenetic studies of *Tettigometrini*.

## 1. Introduction

The family Tettigometridae Germar, 1821 is a small but morphologically distinctive lineage within the Fulgoromorpha, currently comprising fewer than 90 described species and accounting for less than 0.7% of known planthopper diversity (Mozaffarian et al., 2018, Bourgoin, 2025) [1,2]. Despite this limited species count, Tettigometridae remains one of the most taxonomically challenging families at the species level. The scarcity of discrete, non-overlapping diagnostic characters, combined with pronounced intraspecific polymorphism in coloration, body form, and continuous morphological traits, makes species identification particularly difficult.

**Figure 1:**
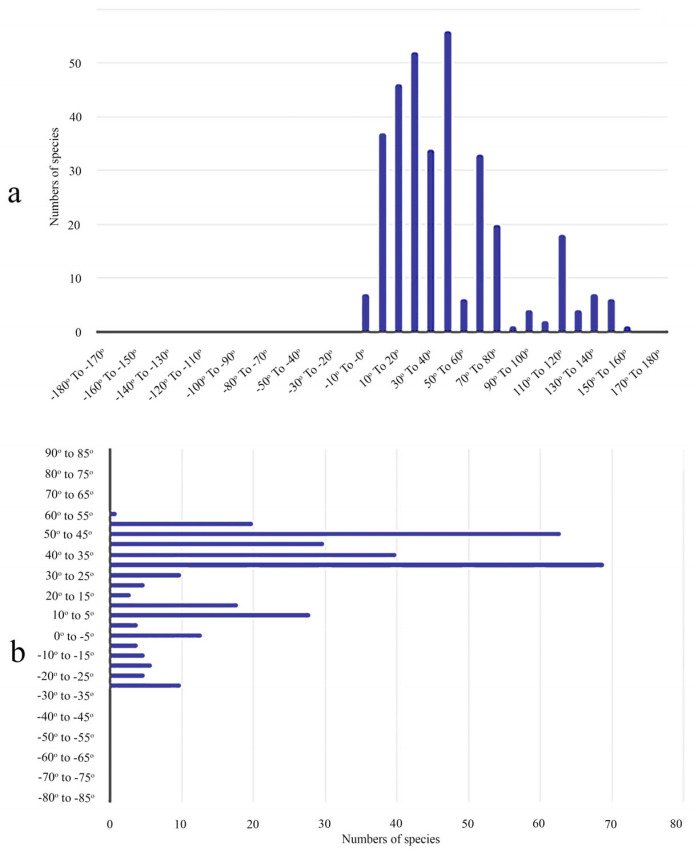
Longitudinal (**a**) and latitudinal (**b**) profile of Tettigometridae in terms of species richness according to (Bourgoin, 2025) [2]. Species numbers represent the count of distinct described species per latitudinal (**b**) and longitudinal (**a**) band (5° intervals), based on published country-level records compiled in the FLOW database. Data are qualitative and reflect species presence only; record frequency and abundance are not considered.

Tettigometridae exhibit a markedly uneven global distribution, with a broad presence across the Afrotropical, Palearctic, and parts of the Oriental regions (Bourgoin, 2025) [2], following a unimodal longitudinal pattern, and are entirely absent from the Nearctic fauna (Bourgoin, 1988a, 1989; Mozaffarian et al., 2018) [1,3,4]. This pattern is reflected in a sharp peak in species richness between 20° E and 70° E (Figure 1a), covering Europe, sub-Saharan Africa, the Middle East, and southwestern Asia, while the Far East show only scattered records. Latitudinally (Figure 1b), their distribution appears bimodal, concentrating first between 25° and 45° N, notably in North Africa, the Mediterranean, and western to central Asia, and with a secondary peak around 0° to 15° S, aligning with their Afrotropical diversity. The family is almost entirely absent from higher latitudes in both hemispheres, confirming a clear ecological preference for warm, often xeric or mesic environments.

As with all planthoppers, tettigometrids are obligate phytophagous insects and some species have been reported as agricultural pests. For instance, *Nototettigometra patruelis* (Stål, 1855) has been found on groundnut crops in Zimbabwe (Weaving, 1980) [5] and on *Vernonia amygdalina* Delile (Asteraceae), a vegetable widely consumed in West Africa (Aléné et al., 2016) [6]. In Europe, Sajó’s earlier observation (1895) [7], probably later reported by Haupt (1935) [8], noted that *Tettigometra obliqua* may occasionally act as a cereal pest, with larvae and nymphs sucking in the leaf axils of wheat and causing complete grain abortion. *Tettigometra hexaspina* has occasionally been recorded as a pest of *Papaver somniferum* L. (Papaveraceae) in Serbia (Gradojević, 1931) [9]. *Tettigometra costulata* was reported by Rajabi (1991) [10] and Abaii (2000) [11] as causing minor damage to orchard trees and young oaks in Iran, although no data has been published on its economic impact (Mozaffarian, 2018) [12]. Damage attributed to *T. virescens* and *T. hexaspina* has also been observed on *Juniperus* spp. in Turkey (Lodos & Kalkandelen, 1980) [13] and on poppy (Wilson & O’Brien, 1987) [14]. However, the most striking feature of tettigometrid biology is their widespread tendency to form long-term trophobiotic associations with ants (Bourgoin et al., 2023) [15].

Morphologically, Tettigometridae exhibits several unique features that have historically complicated its classification among planthopper families. From a phylogenetic perspective, and in contrast to the earlier morphology-based hypotheses that placed Tettigometridae as a sister to all other planthoppers (i.e., basal to both Delphacoidea Leach, 1815 and Fulgoroidea Latreille, 1807), more recent analyses combining detailed morphological data (Bourgoin, 1987) [16] with increasingly robust molecular datasets (Bourgoin et al., 1997; Yeh & Yang, 1999; Yeh et al., 2005; Urban & Cryan, 2007; Bucher et al., 2023; Wang et al., 2023; Ai et al., 2024) [17,18,19,20,21,22,23] now support the inclusion of the family among the more recently diversified lineages of Fulgoroidea *sec.* Bourgoin & Szwedo, 2023 [24]. However, the precise phylogenetic position of Tettigometridae remains unresolved, largely due to insufficient taxon sampling, the weakness of its taxonomic base, and the lack of comprehensive molecular data representing the full extent of the family’s taxonomic diversity (Bucher et al., 2023) [21].

From a classification standpoint, although already recognized as separate family in 1818 [25] (p. 205), Germar only proposed Tettigometridae as a formal family in 1821 [26]; Latreille (1810) [27] had earlier included them within the broader group of ‘Fulgorelles’. In accordance with current phylogenetic evidence, the family is now firmly placed within Fulgoromorpha Fulgoroidea (Bourgoin & Szwedo, 2023) [24]. A comprehensive historical review of the internal classification of Tettigometridae is already available (Mozaffarian et al., 2018) [1], retracing the evolution of its suprageneric structure. Today, the family is divided into four subfamilies: *Phalixinae* Ghauri, 1964, *Egropinae* Baker, 1924 (including the tribes *Egropini* Baker, 1924 and *Cyranometrini* Bourgoin, 1987), *Nototettigometrinae* Bourgoin, 2018 and *Tettigometrinae* Germar, 1821, further subdivided into two tribes: *Plesiometrini* Bourgoin, 2018 and *Tettigometrini* Germar, 1821 (Mozaffarian et al., *op. cit*.) [1]. However, here as well, these subdivisions remain to be rigorously tested and confirmed through molecular phylogenetic analyses.

Within most of these tribes, species-level identification remains particularly challenging due to subtle interspecific differences and considerable intraspecific variation in head morphology, tegmen coloration, and male genitalia (Holzinger et al., 2003) [28]. Moreover, the scarcity of reliable diagnostic morphological characters (especially in female specimens) and the current absence of molecular identification tools further contribute to persistent taxonomic ambiguities. These difficulties are especially pronounced in the large Afrotropical genus *Nototettigometra* Muir, 1924, and particularly in the Palearctic genus *Tettigometra* Latreille, 1804, which is the focus of the present study. In the latter, misidentifications and misinterpretations of species boundaries are particularly frequent and easily made, as already previously reported (Emeljanov, 1980 [28]; Holzinger et al., 2003 [29]).

The genus *Tettigometra* was originally established with two species: *T. obliqua* (Panzer, 1799), later synonymized with *T. leucophaea* (Preyssler, 1792), and *T. virescens* (Panzer, 1799). Latreille (1810) [27] subsequently considered *Tettigometra* part of a broader familiar group within the “Fulgorelles” (planthoppers), and the family Tettigometridae was formally recognized by Germar in 1821 [26]. However, this familial status was not widely adopted until Amyot & Serville (1843) [30] assigned the tribe Planigeni to Tettigometridae. Around twenty years later, Stål (1866) [31] placed *Tettigometra* within the group Issida, and Melichar (1903) [32] subsequently classified it under the family Issidae, in the tribe Tettigometrini. The family Tettigometridae was re-established by Kirkaldy in 1907 [33], with *Tettigometra* as its sole genus. Since then, the genus has expanded to include more than 60 described species (Bourgoin, 2025) [2], making it the most diverse lineage within the family (Mozaffarian et al., 2018) [1].

Efforts to establish a robust suprageneric classification of the genus *Tettigometra s.l.* have been ongoing since the 19th century, in response to persistent challenges in species delimitation and identification. In 1865, Fieber [34] published the first identification key for the 23 species then known, all of them still maintained within a single, undivided genus, *Tettigometra*. A first attempt at generic subdivision followed just a year later, when Signoret (1866) [35]—likely unaware of Fieber’s work—proposed four subgenera based on characters such as the number of antennal segments (sic), the overall shape of the head, and the presence or absence of a raised costal margin on the forewings: *Tettigometra* Latreille, 1804; *Mitricephalus* Signoret, 1866; *Brachycephalus* Signoret, 1866; and *Eurychila* Signoret, 1866. These taxa were later elevated to generic rank within the subfamily Tettigometrinae by Baker (1924) [36], a treatment that was followed by Metcalf (1932) [37]. In contrast, Lindberg (1948) [38] maintained Signoret’s groupings as subgenera [35] under a broadly defined, predominantly Palaearctic genus *Tettigometra*, and introduced two additional subgenera: *Macrometrina* Lindberg, 1948 and *Micrometrina* Lindberg, 1948.

In an effort to produce a more natural classification, Emeljanov (1980) [29] proposed a revised system based primarily on male genital structures (notably the aedeagus), supplemented by a few external morphological traits. He acknowledged that no consistent diagnostic features could be identified to clearly separate *Tettigometra* from other closely related taxonomic units previously described: *Mitricephalus*, *Eurychila*, *Micrometrina*, *Macrometrina*, and *Brachyceps*, and therefore did not consider them to warrant recognition as distinct autonomous genera. Instead, he subdivided *Tettigometra* into eight subgenera: *Tettigometra* Latreille, 1804 (17 species), *Eurychila* Signoret, 1866 (2 species), *Mitricephalus* Signoret, 1866 (8 species), *Macrometrina* Lindberg, 1948 (1 species), and introduced four new subgenera: *Mimarada* Emeljanov, 1980 (1 species), *Hystrigonia* Emeljanov, 1980 (1 species), *Metroplaca* Emeljanov, 1980 (2 species), *Striometra* Emeljanov, 1980 (1 species).

However, in most of these groupings (six out of the eight currently recognized subgenera) the definitions rely on narrowly circumscribed and highly specific characters, often applicable to only one or two species, or on subtle morphological trends that are difficult to delimit in a consistent and operational way. In addition, a number of species were left unassigned to any subgenus, and the published key was presented without accompanying descriptions or formal diagnoses, requiring users to infer defining features solely from the key itself. Combined with the limited number of often vague or weakly discriminant characters employed, this minimalist approach raises concerns not only about the practical usability but also about the taxonomic validity and conceptual soundness of the proposed divisions.

As a result, the efficiency of Emeljanov’s 1980 [29] system remains quite limited and many described species today cannot be confidently assigned to any of the existing subgenera, while several taxa with broad geographic or altitudinal ranges are suspected to be polyphyletic, potentially encompassing cryptic species complexes (Mozaffarian & Bourgoin, 2019) [39]. Consequently, the persistent challenges of species identification and subgeneric delimitation within *Tettigometra* remain largely unresolved.

In this study, we revisit the supraspecific classification of *Tettigometra*, guided by the search for more stable, consistent, and broadly applicable morphological characters. We propose an alternative arrangement of species into smaller, morphologically coherent units. This revised framework, deliberately adopted as a phenetic rather than phylogenetic approach, aims above all to enhance taxonomic resolution, ensure more consistent and reliable identifications, and minimize misclassifications. It constitutes a necessary preliminary step before any meaningful reconstruction of the evolutionary history of these taxa can be undertaken.

## 2. Materials and Methods

### 2.1. Specimens

The primary collections studied were those of the Muséum national d’Histoire naturelle (MNHN) in Paris, France, which include type and reference material identified by specialists such as E. de Bergevin, J. Dlabola, M. Noualhier, L.-F. Lethierry, V.T. Oshanin, A. Puton, H. Ribaut, and possibly Perrier. Additional material was studied from the Hayk Mirzayans Insect Museum (HMIM) in Tehran, Iran, comprising both historical and more recent collections of the family, identified primarily by J. Dlabola and F. Mozaffarian. Further specimens borrowed from the Muséum d’Histoire naturelle de Dijon (MHND), France and the National Museum Cardiff (NMWC) in Wales, UK were also examined in the course of this study.

Both interspecific and intraspecific variation were assessed through comparative analysis of morphological characters. Photographs and detailed illustrations were prepared to document habitus, male genitalia, and other diagnostic features.

Because most type specimens remain unavailable, or in some cases, are based solely on uninformative female specimens, this comparative study primarily relied on identified male specimens from the examined collections. These specimens, often showing morphological variation or bearing alternative identifications, are recorded as separate items in the paper and linked to their original identifiers.

### 2.2. Taxa and Names

Due to numerous identification discrepancies in the literature and collections, taxa were recorded under their taxonomic names contextualized according to their original usage, as reflected in the cited publications or based on specimens identified by those authors in collections. For all taxa listed in this paper, their taxonomic and nomenclatorial status is clarified.

- Taxonomic status. In this revision, all nominal taxa historically assigned to *Tettigometra* were assessed as either valid taxa, recognized as identifiable biological entities that can be diagnosed, and classified under their original or revised combinations, or as *taxa inquirendae,* and type-bearing status where available. The designation *species inquirenda* is not a formal nomenclatural status in the International Code of Zoological Nomenclature (ICZN) but a long-standing taxonomic label, traditionally restricted to the species level.
- Nomenclatural status. All names are treated according to the rules of the ICZN, with explicit mention of their original spelling, authorship. To clarify how names were used in the literature and collections, we followed Bourgoin et al., 2021 [40] in adopting the convention of Berendsohn (1995) [41], adding the qualifier *sec.* (*secundum*, “according to”) to indicate the particular taxonomic concept applied by an author in a given context (e.g., *Taxon name sec.* Author, year or Author coll.). For each taxon, specimens examined were associated with the names under which they were determined, including referenc.es to published illustrations, institutional specimens, and genitalia dissections when available. Nomenclatural clarifications (e.g., corrected original spellings, unjustified emendations, synonymies, homonymies, or changes in rank or combination) are explicitly stated when relevant. This approach ensures a consistent separation between the nomenclatural status of names, governed by the Code, and their taxonomic interpretation, which may vary depending on authors or new evidence.

### 2.3. Methodology

To manage the large volume of data and to facilitate our search for reliable characters suitable for species-level classification within the genus *Tettigometra s.l.*, the database module of the software Xper^2^ 2.3.2 was used [42]. This software, developed by the Muséum national d’Histoire naturelle in Paris, was originally designed to facilitate the creation of interactive identification keys, but also proved highly effective for managing and comparing the large amount of morphological data processed in this study.

For the taxonomic analysis, diagnostic characters were entered into the database as *descriptors* and scored across all items whenever data were available. Specimens were entered into the software as individual *items* with their respective species names, possible varieties, and the collections or cited literature to which they belong, allowing all records to be precisely traced. The “Comparison” function in Xper2 was used to detect identical items, which were then merged where appropriate. This process resulted in a working dataset of 108 items, described using up to 50 descriptors, depending on the availability of material and data in the literature. For species not represented in the collections, character data were extracted from published descriptions and figures whenever possible. A total of 671 photographs and illustrations, including original material and figures from existing publications, were integrated into the database to document habitus, male genitalia, and selected diagnostic traits. Geographic distribution of the recognized genera is listed by the name of the recorded countries, classified in the United Nations geoscheme divisions from west to east worldwide.

**Figure 2:**
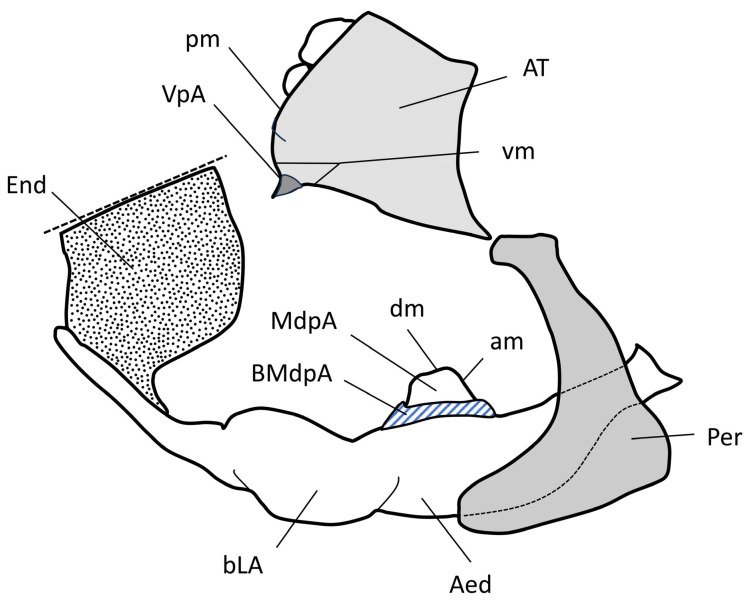
Schematic diagram of male genitalia in *Tettigometra s.l*. Terminology used: am, anterior margin of mediodorsal process of aedeagus; Aed, aedeagus s.s.; AT, anal tube; bLA, basal lobe of aedeagus s.s.; BMdpA, base of mediodorsal aedeagal process; dm, dorsal margin of mediodorsal process of aedeagus; End, membranous endosoma; MdpA, mediodorsal process of aedeagus; Per, periandrium; pm, posterior margin of anal tube; vm, ventral margin of anal tube, anterior and posterior to ventral anal process; VpA, ventral process of anal tube.

Terminology for male genitalia follows Bourgoin (1988b) [43] and Bourgoin & Huang (1990) [44], as illustrated in Figure 2.

Acronyms for collections are as follows:

HMHN: Hayk Mirzayans Insect Museum (Iranian Research Institute of Plant Protection, Tehran, Iran)

MNHN: Muséum national d’Histoire naturelle (Paris, France)

MHND: Muséum d’Histoire naturelle de Dijon (France)

NMWC: National Museum Cardiff (UK)

## 3. Results

**Tribe Tettigometrini Germar, 1821**

⁠

**Type genus.** *Tettigometra* Latreille, 1804 (by subsequent designation of Kirkaldy, 1900) [45].

⁠

**Taxonomic status.** Valid tribe within Tettigometrinae Germar, 1821 (Tettigometridae), restricted to the Palaearctic, corresponding to the nominotypical tribe of the family. It currently includes all the species formerly placed in *Tettigometra* Latreille *s.l.*, including with this paper two taxonomically informal groups, the tettigometrinan and the apexometrinan groups. These two groups encompass all the species previously described under *Tettigometra* Latreille *s.l.*

⁠

**Nomenclatural status.** Name available and valid (Germar, 1821) [26]. Type genus *Tettigometra* Latreille, 1804, designated by Kirkaldy (1900) [45]. *Tettigometra* is also the type genus of the family-group name Tettigometridae.

⁠

**Diagnosis.** Members of Tettigometrini are characterized by the absence of a sub-anal process in males; the gonostyli are distinct, distally hook-shaped, and connected by a lamina gonostyli shorter than the gonostyli. The periandrium is stirrup-shaped, not tubular, surrounding a basally tubular aedeagus developing or not a short impair medio-dorsal aedeagal process; aedeagus (Figure 2) more developed ventrally ending with a membranous endosoma distally developed. Anal tube, short, tube-like, bearing a pair of subapical or submedian ventral processes (Figure 2). Females with a sternite VII divided into three plates posteriorly, including a median ovivalvula, without an additional unpaired plate (versus with in Plesiometrini).

⁠

**Comparative notes**. Within Tettigometrinae, Tettigometrini differ from the Afrotropical Plesiometrini Bourgoin, 2018 by the absence (or strong reduction) of a medioventral process on the pygofer, by their stirrup-shaped periandrium and developed apical endosoma (versus tubular aedeagus only without periandrium, and without membranous endosoma in Plesiometrini), and by the lack of an additional unpaired female sternal plate. They also differ from Nototettigometrinae by the absence of the male sub-anal process and by a different configuration of the female sternite VII (three plates rather than two). Compared to Egropinae and Phalixinae, Tettigometrini are strictly Palaearctic and are distinguished by the general conformation of the male genitalia, the shape and orientation of the gonostyli hooks (turned upward, not latero-externally), the narrower lamina gonostyli, and the periandrium type.

⁠

**Included genera**. Two groups: tettigometrinan and apexometrinan, 14 genera. *Apexometra* Bourgoin & Mozaffarian, ***gen. nov.***; *Brachyceps* Kirkaldy, 1906; *Erratometra* Mozaffarian & Bourgoin, ***gen. nov.***; *Eurychila* Signoret, 1866; *Hystrigonia* Emeljanov, 1980; *Macrometrina* Lindberg, 1948; *Mediodentometra* Bourgoin & Mozaffarian, ***gen. nov.***; *Metroplaca* Emeljanov, 1980; *Micracanthometra* Mozaffarian & Bourgoin, ***gen. nov.***; *Mimarada* Emeljanov, 1980; *Mitricephalus* Signoret, 1866; *Persiametra* Mozaffarian & Bourgoin, ***gen. nov.***; *Stirometra* Emeljanov, 1980; *Tettigometra* Latreille, 1804 s.s.

⁠

**Problematic taxa.** In addition to 9 undescribed species reported in this paper, 14 nominal species historically assigned to *Tettigometra* remain unassignable to any genus within Tettigometrini. Seven of them are treated as Tettigometrini species *incertae sedis* and the seven others as *taxa inquirendae* (Section IV).

⁠

Distribution. Entirely Palaearctic, extending from Western Europe and North Africa through Western, Southern, and Central Asia to East Asia. This contrasts with Afrotropical Plesiometrini and Nototettigometrinae, or with Oriental Egropinae.

⁠

**I. Key to Groups and Genera**

⁠

**Figure 3:**
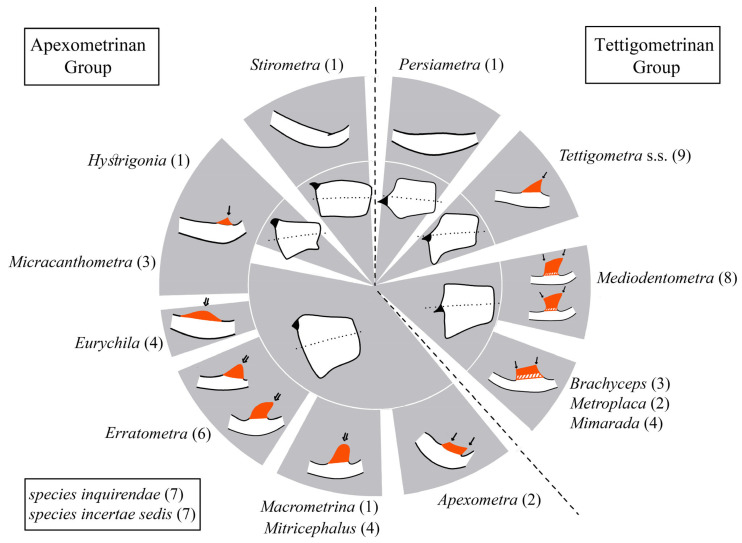
Comparative schematic synthesis of male genital structures within Tettigometrini. The circular diagram contrasts the two main structural groups of the tribe: the apexometrinan group (**left**) and the tettigometrinan group (**right**). Each genus is shown with its number of included species in parentheses, also counting *species inquirendae* and *species incertae sedis*. The inner shape represents the anal tubes with their subapical or median paired ventral processes (in black), while the outer shape depicts the mediodorsal aedeagal process absent, triangular, quadrangular, or finger-like (in red) and their dorsal apexes acute (single arrow) or rounded (double arrow).

**Figure 4:**
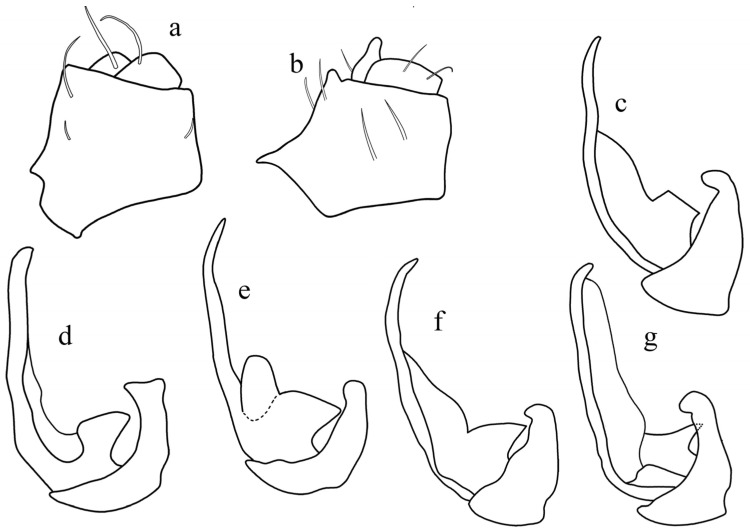
Representative male genitalia in the tettigometrinan group: (**a**,**b**) anal tubes, (**a**) *Mimarada angulata* (Lindberg, 1948) (HMIM), (**b**) *Persiametra pantherina* (Horváth, 1891) (HMIM), (**c**–**g**) aedeagus, (**c**) *Mimarada angulata* (Lindberg, 1948) (HMIM), (**d**) *Mediodentometra sulphurea* (Mulsant et Rey, 1855) (HMIM), (**e**) *Tettigometra virescens* var*. concolor* Fieber, 1865 (MNHN, Ribaut collection), (**f**) *Tettigometra distincta* (Lucas, 1849) (MNHN, Dlabola collection), (**g**) *Tettigometra picea* Kirschbaum, 1868 (MNHN, Puton collection).

**Figure 5:**
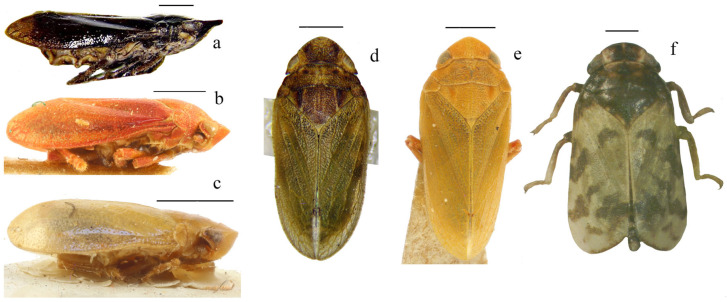
Habitus of six representative species for the six genera of the tettigometrinan group. (**a**) *Mimarada angulata* (Lindberg, 1948) (Hayk Mirzayans Insect Museum) [1], (**b**) *Brachyceps* sanguinea (Lethierry, 1876) (Muséum national d’Histoire naturelle, Puton collection, photo by Laurent Fauvre), (**c**) *Metroplaca baranii* Signoret, 1866 (Muséum national d’Histoire naturelle, Puton collection, photo by Laurent Fauvre), (**d**) *Mediodentometra sulphurea* var. *scutellaris* Horváth, 1903 (Muséum national d’Histoire naturelle, Dlabola collection), (**e**) *Tettigometra virescens* var. *acephala* (Muséum national d’Histoire naturelle, Puton collection, photo by Laurent Fauvre), (**f**) *Persiametra pantherina* (Horváth, 1891) (Hayk Mirzayans Insect Museum) [1]. Scale: 1 mm.

**Figure 6:**
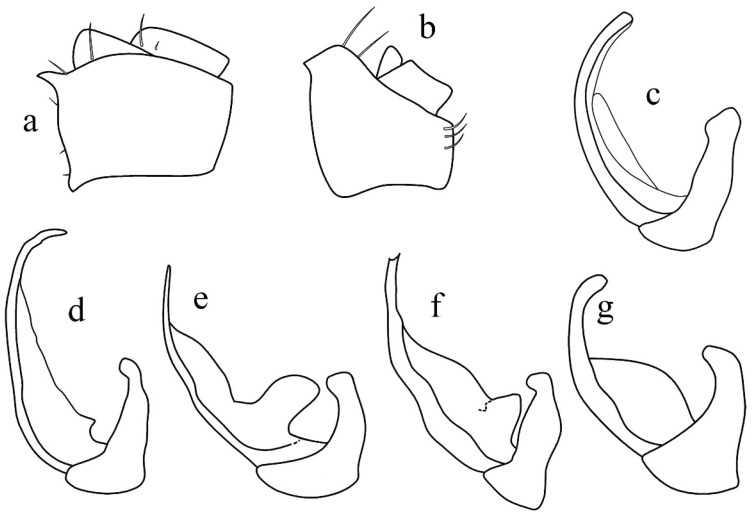
Representative male genitalia in apexometrinan group: (**a**,**b**) anal tubes, (**a**) *Stirometra costulata* (Fieber, 1865) (HMIM), (**b**) *Micracanthometra impressifrons* (Mulsant et Rey, 1855) (HMIM), (**c**–**g**) aedeagus, (**c**) *Stirometra costulata* (Fieber, 1865) (HMIM), (**d**) *Hystrigonia hexaspina* (Kolenati, 1857) (HMIM), (**e**) *Mitricephalus macrocephalus* (Fieber, 1865) (HMIM), (**f**) *Erratometra sordida* (Fieber, 1865) (MNHN, Dlabola collection), (**g**) *Eurychila afra* (Kirschbaum, 1868) (MNHN, Noualhier–Lethierry collection).

1. In lateral view: paired ventral anal processes situated around mid-length of anal tube, protruding from its ventral margin (Figure 3 and Figure 4a,b); the ventral margin itself meets the posterior margin at a distinct angle. When present, mediodorsal aedeagal process in lateral view, truncated, basally triangular or transversely expanded to triangular, not apically rounded (Figure 4c–g): **Tettigometrinan group** ....................................................... 9

– In lateral view: paired anal ventral processes in last third of anal tube, apical or posterior margin of anal tube (Figure 3 and Figure 5a,b), often smoothly continuous with subapical ventral process (Figure 6b); when present, mediodorsal aedeagal process generally apically rounded, or much longer than high if subrectangular (Figure 5e,f): **Apexometrinan group** .......................................................................................................................................................... 2

⁠

2. Dorsal aedeagus margin smooth, without distinct mediodorsal process (Figure 5c,g) .... .......................................................................................................................................................... 3

– Mediodorsal aedeagal process present (minute triangular, quadrangular, or finger-like, Figure 5d,f) .................................................................................................................................... 4

⁠

3. Anal tube longer than high (Figure 3); no mediodorsal aedeagal process, though the aedeagal shaft slightly swollen at this level (Figure 6g) ................... ***Eurychila* Signoret, 1866**

– Anal tube shorter than high (Figure 6a); scutellum carinate; integument matte and rough .......................................................................................................... ***Stirometra* Emeljanov, 1980**

⁠

4. Mediodorsal process strongly developed, triangular, or finger-like, apically rounded (Figure 6e,f) .......................................................................................................................................................... 5

– Mediodorsal process weak, minute triangular, or subrectangular (Figure 6d) .................. 7

⁠

5. Mediodorsal process strongly developed, finger-like, with parallel margins, rounded tip (Figure 6e) ...................................................................................................................................... 6

– Mediodorsal process triangular (Figure 6f) to oval (margins converging), sometimes with constricted base, apex rounded ..... ***Erratometra* Mozaffarian & Bourgoin*, gen. nov.***

⁠

**Figure 7:**
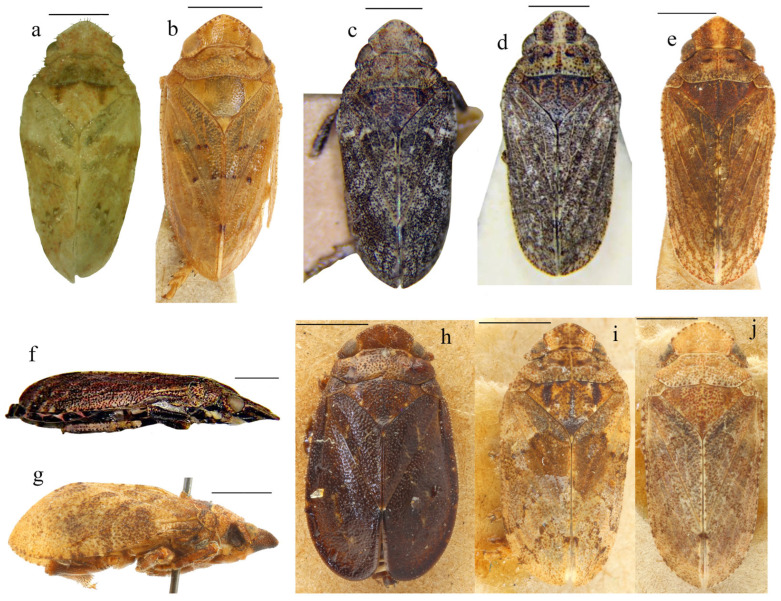
Habitus of six representative species for six genera in apexometrinan group: (**a**) *Hystrigonia hexaspina* (Kolenati, 1857) (Hayk Mirzayans Insect Museum) [1], (**b**) *Micracanthometra picta* (Fieber, 1865) (Muséum national d’Histoire naturelle, Puton collection, photo by Laurent Fauvre), (**c**) *Apexometra sororcula* (Horváth, 1897) (Muséum national d’Histoire naturelle, Ribaut collection), (**d**) *Apexometra griseola* (Fieber, 1865) (Muséum national d’Histoire naturelle, Ribaut collection), (**e**) *Apexometra griseola* (Fieber, 1865) (Muséum national d’Histoire naturelle, Puton collection, photo by Laurent Fauvre), (**f**) *Mitricephalus macrocephalus* (Fieber, 1865) (Hayk Mirzayans Insect Museum) [1], (**g**) *Macrometrina fusca* (Melichar, 1902) (Muséum national d’Histoire naturelle, Noualhier–Lethierry collection, photo by Laurent Fauvre), (**h**) *Eurychila afra* (Kirschbaum, 1868) (Muséum national d’Histoire naturelle, Noualhier–Lethierry collection, photo by Laurent Fauvre), (**i**) *Stirometra costulata* (Fieber, 1865) (Muséum national d’Histoire naturelle, Puton collection, photo by Laurent Fauvre), (**j**) *Erratometra sordida* (Fieber, 1865) (Muséum national d’Histoire naturelle, Puton collection, photo by Laurent Fauvre). Scale: 1 mm.

6. Tegminae in lateral view more elevated posteriorly over the body, (Figure 7g) ...................................................................................................... ***Macrometrina* Lindberg, 1948**

– Tegminae in lateral view not more elevated posteriorly over the body (Figure 7f) ........................................................................................................ ***Mitricephalus* Signoret, 1866**

⁠

7. Mediodorsal process quadrangular, transverse, weakly elevated, dorsal margin concave ...................................................................... ***Apexometra* Bourgoin & Mozaffarian, *gen. nov.***

– Mediodorsal process minute, pointed, triangular (Figure 6d) .............................................. 8

⁠

8. Anal tube short: higher than long in lateral view with dorsal margin strongly concave (Figure 3). Body covered with long bristle-like setae (macrosetae) conspicuous on all the body (Figure 7a) ........................................................................ ***Hystrigonia* Emeljanov, 1980**

– Anal tube long (Figure 6b): longer than high in lateral view with dorsal margin more or less straight. Such long bristle-like setae absent (body glabrous or with only short ordinary setae) ...................................... ***Micracanthometra* Mozaffarian & Bourgoin, *gen. nov.***

⁠

9. Mediodorsal aedeagal process absent, paired ventral processes of anal tube long (Figure 4b) ................................................................ ***Persiametra* Mozaffarian & Bourgoin*, gen. nov.***

– Mediodorsal aedeagal process distinct, triangular, or quadrangular (Figure 4c–g), paired ventral process of anal tube short (Figure 4a) ........................................................................ 10

⁠

10. Dorsal aedeagal process quadrangular, truncated, wider than high (Figure 4c) ............. 11

– Dorsal aedeagal process triangular, high, apically pointed, with or without quadrangular base (Figure 4d–g) ................................................................................................................ 13

⁠

11. Frons concave Figure 5a, vertex produced ........................ ***Mimarada* Emeljanov, 1980**

– Frons convex to straight ............................................................................................................ 12

⁠

12. Frons convex Figure 5c, vertex evenly rounded; tegmina shiny or not ........................................................................................................ ***Metroplaca* Emeljanov, 1980**

– Frons somewhat straight Figure 5b, vertex rounded; tegmina matte .................................... .............................................................................................................***Brachyceps* Kirkaldy, 1906**

⁠

13. Dorsal aedeagal process directly triangular (single-pointed), without quadrangular base (Figure 4e–g) ........................................................................ ***Tettigometra* Latreille, 1804 (*s.s.*)**

– Dorsal aedeagal process globally quadrangular, with distinct base, constricted or not, upper margin bi-pointed, anterior angle more pronounced (Figure 3 and Figure 4d) ........................................................... ***Mediodentometra* Bourgoin & Mozaffarian*, gen. nov.***

⁠

Summarizing in a single view this new generic framework, Figure 3 provides a schematic synthesis of the main structural patterns of male genitalia observed within *Tettigometrini* and their distribution among the different genera. In particular, the diagram illustrates the variation of two key diagnostic elements: the conformation of the anal tube and the location of its paired ventral processes and the shape of the mediodorsal aedeagal process. The circle is divided into two nearly symmetrical halves corresponding to the two structural groups recognized in this study: the apexometrinan group on the left and the tettigometrinan group on the right. For each genus, the inner ring depicts a general morphology of the anal tube, while the outer ring represents a schematic outline of the mediodorsal aedeagal process (absent, triangular, quadrangular, or finger-like). As no phylogenetic hypothesis is currently available or proposed in this study, the figure does not imply any evolutionary sequence. Rather, it provides a visual framework for comparing genera both vertically (between the two major groups) and horizontally (between corresponding sectors), thereby highlighting recurrent patterns and possible parallel developments in genital structures within the tribe.

**II. The tettigometrinan group**

⁠

**Taxonomic status**. Phenetic, morphologically defined group within Tettigometrini Germar, 1821. It is used here as a practical framework to delimit a group of genera sharing a common set of diagnostic morphological characters, distinct from taxa in the apexometrinan group, but it is not treated as a formal taxonomic group.

⁠

**Diagnosis**. Species assigned to the tettigometrinan group are characterized, in lateral view, by a ventral process of the anal tube protruding ventrally, positioned slightly after mid-length along the ventral margin (Figure 4a,b). This contrasts with the apical to subapical positioning observed in the apexometrinan group (Figure 3). In this configuration, the posterior and ventral margins of the anal tube meet at a distinct angle, rather than forming a continuous curve as in most apexometrinan taxa.

⁠

The mediodorsal aedeagal process is variable across genera: It may be absent in *Persiametra* Mozaffarian & Bourgoin, ***gen. nov.***, triangular in *Tettigometra* s.s. (Figure 4e–g), or quadrangular-trapezoidal in all other genera of the group, but it is always apically acute or truncate, never rounded or elongate finger-like (in contrast to apexometrinan taxa). When quadrangular-trapezoidal, its dorsal margin is transverse, straight to slightly convex or concave, and it is more strongly developed than the parallel forms observed in the apexometrinan genus *Apexometra Bourgoin & Mozaffarian*, ***gen. nov.*** (Figure 3). The vertex in dorsal view ranges from evenly rounded to slightly medially produced; in lateral view, the frons may appear concave or convex. The tegmina can be shiny or matte, with or without a marginal costal groove.

⁠

**Note**. The tettigometrinan group forms a coherent assemblage based on male genital structures, which provide the main basis for the generic delimitations adopted in this revision. The unusual median position of the anal tube ventral process may represent a derived apomorphic condition. This supports the interpretation of the group as a potentially natural lineage, possibly deserving formal recognition at subtribal level in the future, pending molecular phylogenetic confirmation.

⁠

**Included genera**. *Brachyceps* kirkaldy 1906, ***stat. rev.***; *Mediodentometra* Bourgoin & Mozaffarian & Bourgoin, ***gen. nov.***; *Metroplaca* Emeljanov, 1980 ***stat. nov.*;** *Mimarada* Emeljanov, 1980, ***stat. nov.***; *Persiametra* Mozaffarian & Bourgoin, ***gen. nov.***; *Tettigometra* Latreille, 1804 (*sensu stricto*, as interpreted herein).

⁠

**1. Genus *Brachyceps* Kirkaldy 1906 *stat. rev.***

*Brachycephalus* Signoret, 1866 *nom. praeocc.* Kirkaldy, 1906

⁠

**Type species**. *Tettigometra (Brachycephalus) lucida* Signoret, 1866 by subsequent designation (Baker, 1924: 93) [36], synonymized with *Tettigometra brachycephala* Fieber, 1865 by Puton, 1886 [46].

⁠

**Taxonomic status**. *Brachyceps* Kirkaldy, 1906 is reinstated here as a valid genus (***stat. rev.***).

⁠

**Nomenclatural status**. Name available and validly published (Kirkaldy, 1906) [47]. *Brachycephalus* Signoret, 1866 is an unavailable junior homonym (ICZN Art. 52). Type species designation follows Baker (1924) [36]. The synonymy of *lucida* with *brachycephala* follows Puton (1886) [46] and is accepted herein. Gender: masculine.

⁠

**Diagnosis**. As in all species of the tettigometrinan group, the ventral process of the anal tube is positioned approximately at mid-length along the ventral margin in lateral view. Along with species of *Metroplaca* and *Mimarada*, those of *Brachyceps* are characterized by a nearly truncated dorsal aedeagal process, which is wider than high in lateral view and forms a broad, non-rounded rectangular and truncated shape (Figure 4c), in contrast to the triangular or trapezoidal processes respectively observed in *Tettigometra s.str.* ***stat. nov.*** and *Mediodentometra* Bourgoin & Mozaffarian, ***gen. nov.*** With *Metroplaca, Brachyceps* share a vertex not strongly produced anteromedially, leaving a frons convex in lateral view, as opposed to the medially produced anterior margin of the vertex in *Mimarada* but showing a concave frons in lateral view. The posterior lateral margin of periandrium in lateral view is straight to regularly concave and its ventral margin more than twice as long as the mediodorsal process, which is distinctly shorter in *Metroplaca*. *Brachyceps* also differs from *Metroplaca* by matte tegmina.

⁠

**Taxonomic note**. The taxon was originally described as a subgenus of *Tettigometra* and later elevated to genus rank by Metcalf (1932) [37]. Emeljanov (1980) [29] synonymized it under *Tettigometra* mainly by default, relying on the absence rather than the presence of diagnostic characters. *Brachyceps* Kirkaldy, 1906 is here reinstated as a valid genus (***stat. rev.***), replacing *Brachycephalus* Signoret, 1866, an unavailable junior homonym recognized by Kirkaldy, 1906) [47], who also designated *Brachycephalus lucida* as the type species of the subgenus at this time. This species was previously placed in synonymy with *Tettigometra brachycephala* Fieber, 1865 by Puton (1886) [46], a synonymy accepted herein. In accordance with Article 67.8 of the International Code of Zoological Nomenclature (ICZN), the nominal type species of *Brachyceps* remains *lucida*, but the taxonomic type corresponds to *Tettigometra brachycephala*.

⁠

- **Included species**. *Brachyceps brachycephala* (Fieber, 1865) ***comb. nov.***; *Brachyceps laeta* (Herrich-Schäffer, 1835) ***comb. nov.***; *Brachyceps* sanguinea (Lethierry, 1876) ***comb. nov.***

⁠

**Geographical distribution:** Northern Africa [Algeria, Morocco, Tunisia], Southern Europe [Italy, Greece, Malta, Montenegro, Spain, Macedonia, Serbia, Slovenia], Western Europe [Austraia, Belgium, Germany, France, Netherlands], Eastern Europe [Bulgaria, Czech Republich, Hungary, Moldova, Poland, Romania, Slovakia, Ukraine], Western Asia [Turkey] (Bourgoin, 2025 [2], Fokker, 1900 [48]; Jankovic, 1966, [49], 1971a [50], Seljak, 2004 [51] Nast, 1972 [52]; Lehouck et al., 2004 [53]; Krstic et al., 2012 [54]; Gebicki et al., 2013 [55]; Albre & Gibernau, 2019 [56]; D’Urso et al., 2019 [57]; Den Bieman et al., 2020 [58]).

⁠

**Specimens studied:** 

- ***Brachyceps brachycephala* (Fieber, 1865)** ***comb. nov.***

*Tettigometra brachycephala* Fieber, 1865 (original combination)

=*Tettigometra tumidifrons* Kirschbaum, 1868: 57 (syn. by Fieber, 1872a: 30)

=*Tettigometra (Brachycephalus) lucida* Signoret, 1866 (syn. by Puton, 1886: 75)

**→***Brachyceps brachycephala* (Fieber, 1865) (transfer by Metcalf, 1932: 58)

**→***Tettigometra* (*Tettigometra*) *brachycephala* Fieber, 1865 (transfer by Emeljanov, 1980)

**→***Brachyceps brachycephala* (Fieber, 1865) ***comb. nov.*** (present transfer)

⁠

**Taxonomic status**. Valid species of *Brachyceps*, with synonymies proposed without explicit justification [46] but consistently accepted by later authors.

**Nomenclatural status.** Name available and valid (Fieber, 1865) [34]. *T. tumidifrons* Kirschbaum, 1868 and *T. (Brachycephalus) lucida* Signoret, 1866 were synonymized with *B. brachycephala* by Fieber (1872a) [59] and Puton (1886) [46], respectively. The Puton specimen labeled as *T. lucida* in MNHN was most likely the basis for Puton’s synonymy. Metcalf (1932) [37] first followed Kirkaldy’s (1906) [47] new genus name for the species which was later transfered by Emeljanov (1980) [29] in *Tettigometra (Tettigometra)*.

⁠

**Illustrated**. Lindberg (1948, based on non-type material) [38].

**Identified specimens**. Bergevin, Dlabola, Oshanin, and Puton (MNHN); one specimen identified as *T. lucida* (MNHN, coll.), later relabeled by G. Kunz as *T. brachycephala* (“Holotype removed from Fieber type box”).

**Dissected material**. Male genitalia examined from the Dlabola collection (MNHN).

⁠

- ***Brachyceps laeta*** **(Herrich-Schäffer, 1835)** ***comb. nov.***

*Tettigometra laeta* Herrich-Schäffer, 1835 (original combination)

=*Tettigometra lepida* Fieber, 1876 (nomen nudum Fieber, 1872b: 2 [60]; validated and syn. by Horváth, 1897a: 54 [61]; see Metcalf, 1932: 36) [37].

**→** Brachyceps laeta (Herrich-Schäffer, 1835) ***comb. nov.*** (present transfer)

⁠

**Taxonomic status**. Valid species of *Brachyceps*.

**Nomenclatural status**. Name available and valid (Herrich-Schäffer, 1835) [62]. *T. lepida* Fieber, 1876 is treated as its junior synonym (Horváth, 1897a) [61].*T. lepida* Fieber, 1876 was long considered problematic and as a *nomen nudum* (Fieber, 1872b) [60], but it has been subsequently validated and treated as junior synonym of *T. laeta* by Horváth (1897a) [61].

⁠

**Illustrated**. Lindberg (1948, based on non-type material) [38]

**Identified specimens**. Bergevin and Dlabola (MNHN coll.); *T. lepida* Fieber, 1835 identified by Noualhier–Lethierry and Puton (MNHN coll.).

**Dissected material**. Male genitalia examined from the Dlabola collection (MNHN).

⁠

- ***Brachyceps*** ***sanguinea*** **(Lethierry, 1876)** ***comb. nov.***

Figure 5b

*Tettigometra sanguinea* Lethierry, 1876 (original combination)

**→** *Brachyceps sanguinea* (Lethierry, 1876) ***comb. nov.*** (present transfer)

⁠

**Taxonomic status**. Valid species of *Brachyceps*.

**Nomenclatural status.** Name available and valid (Lethierry, 1876) [63]; not addressed by Emeljanov (1980) [29].

⁠

**Identified specimens.** Dlabola; Noualhier–Lethierry and Puton (MNHN coll.).

**Dissected material**. Male genitalia examined from the Dlabola collection (MNHN).

⁠

**2. Genus *Metroplaca* Emeljanov, 1980, *stat. nov.***

⁠

**Type species**. *Tettigometra (Brachycephalus) longicornis* Signoret, 1866 (designated by Emeljanov, 1980) [29].

⁠

**Taxonomic status**. *Metroplaca* Emeljanov, 1980 is here elevated to full generic status (***stat. nov.***). Originally described as a subgenus of *Tettigometra*, it includes two species: *Brachycephalus baranii* Signoret, 1866 and *Brachycephalus longicornis* Signoret, 1866, the latter designated as the type species by Emeljanov (1980) [29].

**Nomenclatural status**. Name available and valid (Emeljanov, 1980) [29]. Type species designation follows Emeljanov (1980) [29]. The original combinations of the included species (*Brachycephalus baranii* and *B. longicornis*) are validly published names (Signoret, 1866) [35], here transferred to *Metroplaca*. Gender: feminine.

⁠

**Diagnosis**. A genus closely related to *Brachyceps*, with which it shares similar male genitalia character states: medioventral anal process and mediodorsal aedeagal process quadrangular. In *M. longicornis*, presence of a distinct shallow groove along the costal margin of the forewings, which are knife-shaped, broadened, and flattened upwards and outwards. Tegmina characteristically shiny (Emeljanov, 1980) [29], in contrast to the matte tegmina of *Brachyceps*. It also separates from *Brachyceps* by a shorter ventral margin of the periandrium (less than twice as long as the process in lateral view) and by the lateral posterior margin, which is slightly convex in its upper part (although concave in *M. longicornis sec.* Emeljanov, 1980: Figure 3.6) [29].

⁠

**Taxonomic note**. *Metroplaca* Emeljanov, 1980 is here elevated to full generic status (***stat. nov.***) in the present treatment. Originally proposed as a subgenus of *Tettigometra*, it includes two species originally described by Signoret (1866) [35] as *Brachycephalus baranii* and *Brachycephalus longicornis*. The latter was designated as the type species of *Metroplaca* by Emeljanov (1980) [29].

⁠

**Included species**. *Metroplaca baranii* (Signoret, 1866) ***comb. nov.***; *Metroplaca longicornis* (Signoret, 1866)**.**

⁠

**Geographical distribution:** Northern Africa [Algeria], Southern Europe [Albania, Italy, Greece, Spain], Western Europe [France, Germany, Switzerland], Eastern Europe [Bulgaria, Czech Republic, Romania], Western Asia [Armenia, Azerbaijan, Isreal, Turkey], Southern Asia [Afghanistan, Iran], Central Asia [Kazakhstan, Tajikistan, Turkmenistan, Uzbekistan] (Mitjaev, 1971 [64]; Nast, 1972 [52]; Demir, 2006 [65]; Mozaffarian et al., 2018 [1]; Bourgoin, 2025 [2]).

⁠

**Specimens studied:**

- ***Metroplaca baranii*** **(Signoret, 1866) *comb. nov.***

*Tettigometra (Brachycephalus) baranii* Signoret, 1866 (original combination)

→*Metroplaca baranii* (Signoret, 1866) ***comb. nov.*** (present transfer)

⁠

**Taxonomic status**. Valid species of *Metroplaca*. Considered distinct from *T. diminuta* Matsumura, 1910 by Metcalf (1932) [37] and Lindberg (1948) [38]. The synonymy proposed by Holzinger et al. (2003) [28] with *T. diminuta* Matsumura, 1910, remains to be justified and this species is left as *species inquirenda*.

**Nomenclatural status**. Name available and valid (Signoret, 1866) [35]. No type specimen known.

**Illustrated**. Lindberg (1948) [38] and Mitjaev (1971) [64], both under this name, from non-type material.

**Identified specimens**. Noualhier–Lethierry, Oshanin, Puton, and Ribaut (MNHN collections).

**Dissected material**. Male genitalia examined from the Puton collection (MNHN).

⁠

**Note**. Identification appears stable across authors, though all references are based on non-type material. *T. baranii* and *T. diminuta* Matsumura, 1910 were treated as distinct species by Lindberg (1948: 32, listed as species no. 34 and 35) [38], but synonymyzed by Holzinger et al. (2003) [28] (see further comments in Section IV).

⁠

- ***Metroplaca longicornis*** **(Signoret, 1866)**

*Tettigometra (Brachycephalus) longicornis* Signoret, 1866 (original combination)

→*Metroplaca longicornis* (Signoret, 1866) (transfer by Emeljanov, 1980)

⁠

**Taxonomic status**. Valid species of *Metroplaca* and type species of the genus (by designation of Emeljanov, 1980) [29]. *Tettigometra longicornis sec.* illust. Mitjaev, 1971 (Figures 18, 35–37) [64] belong to another taxon in the apexometrinan group in the new genus *Apexometra* Bourgoin & Mozaffarian, ***gen. nov.***

**Nomenclatural status.** Name available and valid (Signoret, 1866) [35]. Type species fixation by Emeljanov (1980) [29].

⁠

**Illustrated**. Lindberg (1948) [38], and Emeljanov (1980) [29], under this name, from non-type specimens.

**Identified specimens**. Bergevin, Noualhier, Lethierry, and Puton (MNHN collections).

⁠

**3. Genus *Mimarada* Emeljanov, 1980, *stat. nov.***

⁠

**Type species.** *Tettigometra bipunctata* Matsurmura, 1900 by original designation and monotypy.

⁠

**Taxonomic status**. *Mimarada* Emeljanov, 1980 is here elevated to full generic status (***stat. nov.***).

⁠

**Nomenclatural status**. Name available and valid (Emeljanov, 1980) [29]. Type species fixation by original designation and monotypy (Emeljanov, 1980) [29]. Gender: feminine.

⁠

**Diagnosis**. Ventral process of the anal tube positioned medially on its ventral margin in lateral view (Figure 4a). In lateral view, periandrium with posterior margin concave, or straight and then angularly concave; the ventral margin is long, more than twice the length of the mediodorsal process. *Mimarada* shares with *Brachyceps* and *Metroplaca* an almost truncated dorsal aedeagal process that is wider than high in lateral view, forming a broad, non-rounded rectangular shape (Figure 4c), as opposed to the triangular or trapezoidal shaped processe observed respectively in *Tettigometra* ***stat. nov.*** and *Mediodentometra* Bourgoin & Mozaffarian, ***gen. nov.*** *Mimarada* is further distinguished by the anterior margin of the vertex being medially produced, which results in a concave frons in lateral view, contrasting with the regularly more rounded vertex and convex frons observed in *Brachyceps* and *Metroplaca*.

⁠

**Included species**. *Mimarada akramowskajae* (Lindberg, 1960) ***comb. nov.***; *Mimarada angulata* (Lindberg, 1948) ***comb. nov.***; *Mimarada bipunctata* (Matsumura, 1900); *Mimarada pseudovitellina* (Mitjaev, 1971) ***comb. nov.***; *Mimarada vitellina* (Fieber, 1865) ***comb. nov.***; *Mimarada ziaratensis* (Sohail et al., 2020) ***comb. nov.***

⁠

**Note.** The three species *M. vitellina*, *M. pseudovitellina*, and *M. ziaratensis* cannot be confidently distinguished based solely on their original descriptions. Available illustrations (Lindberg, 1948 [38]; Logvinenko, 1975 [66]) suggest that in *M. vitellina*, the dorsal margin of the aedeagal dorsal process is distinctly concave. However, none of the specimens we examined, including those identified as *M. vitellina* by Dlabola in the HMIM collections, showed such a marked concavity; instead, they exhibited a nearly straight dorsal margin, as illustrated for *M. pseudovitellina* by Mitjaev (1971) [64]. In *M. ziaratensis*, the dorsal concavity appears to accentuate the anterior tip of the process more than the posterior one. Pending molecular data and further material, we tentatively maintain these three taxa as distinct species, although the observed differences may ultimately prove to represent intraspecific polymorphism within a single species.

⁠

**Problematic taxa assigned to *Mimarada***. *T. beckeri* Horváth, 1909 *sec.* Mitjaev (1971), *nec auth.*; unidentified taxon treated as *T. fusca* Fieber, 1865 *sec.* Mitjaev (1971) or as *T. cerina* Lindberg, 1948 *sec.* Dlabola (MNHN coll.).

⁠

**Geographical distribution:** Southern Europe [Serbia], Eastern Europe [Russia, Ukraine], Western Asia [Armenia, Azerbaijan, Georgia, Israel, Turkey], Southern Asia [Afghanistan, Iran, Pakistan], Central Asia [Kazakhstan, Kirghizia, Tajikistan, Turkmenistan, Uzbekistan], Northern Asia [Russian Far East], Eastern Asia [Japan, Korea, Mongolia] (Mozaffarian et al., 2018 [1]; Bourgoin, 2025 [2]; Lindberg, 1948 [38]; Jankovic (1966) [49]; Nast, 1972 [52]; Mitjaev, 1971 [64]; Demir, 2006 [65]; Logvinenko, 1975 [66]; Dlabola, 1957 [67]; Anufriev & Emeljanov, 1988 [68]; Mozaffarian & Wilson, 2011 [69]; Hayashi & Fujinuma, 2016 [70]; Sohail et al., 2020 [71]).

⁠

**Specimens studied:**

- ***Mimarada akramowskajae* (Lindberg, 1960) *comb. nov.***

*Eurychila akramowskajae* Lindberg, 1960 (original combination)

→*Mimarada akramowskajae* (Lindberg, 1960) ***comb. nov.*** (present transfer)

⁠

**Taxonomic status.** Valid species of *Mimarada*, here transferred from *Tettigometra* (*Eurychila)* (*sec.* Lindberg, 1960).

**Nomenclatural status.** Name available and valid (Lindberg, 1960: 63) [72]. No synonymies known.

⁠

**Illustrated.** Lindberg (1960: Figure 1a–c, based on type material) [72]. Later cited by Nast (1972) [52].

**Identified specimens.** No specimen was available for examination in the present study.

⁠

**Note.** Lindberg (1960) [72] originally described the species under *Eurychila*, based on two specimens from Armenia. Although he stated on p. 58 that two females were available, the description on p. 62 clearly refers to one male and one female. According to Lindberg’s figures, particularly the male genitalia (Figure 1c), the species belongs to the tettigometrinan group, characterized by a quadrangular mediodorsal aedeagal process. This feature is diagnostic for genera such as *Brachyceps*, *Metroplaca*, and *Mimarada*, and contrasts with *Eurychila*, where the dorsal process is reduced to a single swelling of the aedeagal shaft. The transfer to *Mimarada* is further supported by external characters noted in Lindberg’s description: vertex broadly rounded, shortly produced in front of the eyes dorsally, frons apically concave in lateral view, absence of pubescence, and a uniformly pale yellow, matte body coloration.

⁠

- ***Mimarada angulata* (Lindberg, 1948) *comb. nov.***

Figure 5a

*Tettigometra angulata* Lindberg, 1948 (original combination)

→*Mimarada angulata* (Lindberg, 1948) ***comb. nov.*** (present transfer)

⁠

**Taxonomic status.** Valid species of *Mimarada*.

**Nomenclatural status.** Name available and valid (Lindberg, 1948) [38].

⁠

**Illustrated.** Lindberg (1948, type material) [38]; Mitjaev (1971 [64], non-type material).

**Identified specimens.** Dlabola (MNHN coll., HMIM coll.); Mozaffarian (HMIM coll.).

**Dissected material.** Male genitalia examined in HMIM (Figure 4c).

⁠

- ***Mimarada bipunctata* (Matsumura, 1900)**

*Tettigometra bipunctata* Matsumura, 1900 (original combination)

=*Tettigometra shikokuana* Ishihara, 1954 (syn. by Emeljanov, 1977: 100)

→*Mimarada bipunctata* (Matsumura, 1900) (transfer by Emeljanov, 1980)

⁠

**Taxonomic status.** Valid species of *Mimarada*; type species of the genus.

**Nomenclatural status.** Name available and valid (Matsumura, 1900) [73]. Type species fixation by original designation and monotypy (Emeljanov, 1980) [29]. *T. shikokuana* Ishihara, 1954 was synonymized with *T. bipunctata* by Emeljanov (1977) [74].

⁠

**Illustrated.** Emeljanov (1980) [29]; Anufriev & Emeljanov (1988) [68], both from non-type material.

**Identified specimens.** No specimen was available for examination in the present study.

⁠

- ***Mimarada pseudovitellina* (Mitjaev, 1971) *comb. nov.***

*Tettigometra pseudovitellina* Mitjaev, 1971 (original combination)

→*Tettigometra* (*Tettigometra*) *pseudovitellina* Mitjaev, 1971 (Emeljanov, 1980)

→*Mimarada pseudovitellina* (Mitjaev, 1971) (present transfer)

⁠

**Taxonomic status.** Valid species of *Mimarada*, though potentially difficult to separate from *M. vitellina* and *M. ziaratensis*.

**Nomenclatural status.** Name available and valid (Mitjaev, 1971) [64].

⁠

**Illustrated.** Mitjaev (1971, based on type material) [64].

**Identified specimens.** One specimen, identified by Dlabola (MNHN).

**Dissected material.** Male genitalia examined from the Dlabola collection (MNHN).

⁠

- ***Mimarada vitellina* (Fieber, 1865) *comb. nov.***

*Tettigometra vitellina* Fieber, 1865 (original combination)

=*Tettigometra pallicornis* Signoret, 1866 (syn. by Fieber 1872b: 2)

→*Tettigometra* (*Tettigometra*) *vitellina* Mitjaev, 1971 (Emeljanov, 1980)

→*Mimarada vitellina* (Fieber, 1865) here transferred.

⁠

**Taxonomic status.** Valid species of *Mimarada*, though potentially difficult to separate from *M. pseudovitellina* and *M. ziaratensis*.

**Nomenclatural status.** Name available and valid (Fieber, 1865) [34].

⁠

**Illustrated.** Lindberg (1948, based on type material) [38].

**Identified specimens.** Dlabola, Mozaffarian (MNHN and HMIM).

**Dissected material.** Male genitalia examined in MNHN an HMIM.

⁠

- ***Mimarada ziaratensis* (Sohail et al., 2020) *comb. nov.***

*Tettigometra ziaratensis* Sohail et al., 2020 (original combination)

→*Mimarada ziaratensis* (Sohail et al., 2020) ***comb. nov.*** (present transfer)

⁠

**Taxonomic status.** Valid species of *Mimarada*, tentatively maintained separated from *M. vitellina* and *M. pseudovitellina*.

**Nomenclatural status.** Name available and valid (Sohail et al., 2020) [71].

⁠

**Illustrated.** Sohail et al. (2020, type material, photographs and description) [71].

**Identified specimens.** No specimen was available for examination in the present study.

⁠

**Problematic taxa assignated to the genus *Mimarada***

- **Unidentified (misapplied, new?) species**

=*Tettigometra beckeri* Horváth, 1909 *sec.* Mitjaev (1971), *nec auth.*

⁠

**Taxonomic status.** Misidentified taxon; the specimen figured by Mitjaev (1971) [64] as *T. beckeri* belongs to this genus *Mimarada*, and is not a true *T. beckeri* that belongs to the genus *Mediodentometra* Bourgoin & Mozaffarain ***gen. nov.*** (see further)

**Nomenclatural status.** name valid but misapplied usage by Mitjaev (1971) [64].

⁠

**Illustrated.** Mitjaev (1971: Figures 18, 21–24) [64].

⁠

**Note.** The species illustrated and described as *T. beckeri* by Mitjaev (1971) [64] does not correspond to the taxon *T. beckeri sensu* Horváth, nor to later interpretations by Lindberg (1948: Figure 18B) [38] and Logvinenko, 1975 [66]. Mitjaev’s concept, here cited as *T. beckeri sec.* Mitjaev (1971), shows characters typical of *Mimarada*, in contrast to *T. beckeri sensu* Horváth, which is transferred to the new genus *Mediodentometra*. The material figured by Mitjaev (1971) [64] is therefore considered a misidentified, possibly undescribed species within *Mimarada*. True *T. beckeri* is here transferred to *Mediodentometra **gen. nov.***

⁠

- **Unidentified (new?) species**

=*Tettigometra fusca* (Fieber, 1865) *sec.* Mitjaev (1971: Figures 18, 4–7)

=?*Tettigometra cerina* Lindberg, 1948 *sec.* Dlabola (MNHN coll., dissected)

⁠

**Taxonomic status.** Unidentified, possibly new taxon, tentatively placed in *Mimarada*.

**Nomenclatural status.** Misapplied names (*T. fusca sec.* Mitjaev, 1971; *T. cerina sec.* Dlabola, identif.).

⁠

**Illustrated.** Mitjaev (1971: Figures 18, 4–7) [64].

**Identified specimens.** Dlabola (MNHN).

**Dissected material.** Male genitalia examined from the Dlabola collection (MNHN)

⁠

**Note.** This unidentified, possibly new taxon is assigned to the genus *Mimarada*, based on the illustrations provided by Mitjaev (1971: Figures 4–7 and 18) [64]. It is represented by two different species concepts: (1) a dark brown *Tettigometra fusca* Fieber, 1865 as interpreted and illustrated by Mitjaev (1971) [64], and (2) a paler form identified by Dlabola as *T. cerina* Lindberg, 1948 in the MNHN collection (male genitalia dissected). Both belong to the same taxon concept and share key diagnostic features: a dorsally straight, truncated aedeagal dorsal process that is longer anteriorly than posteriorly, and a frons in lateral view, similar to the illustration of frons for *T. cerina* by Lindberg (1948) [38]. The true *T. cerina sec. author* is placed in *Mitricephalus* in this study. Specimens of the unidentified taxon are only known from Kazakhstan (Mitjaev, 1971; MNHN collections).

⁠

Lindberg (1948) [38] did not illustrate the male genitalia of *T. fusca*. However, the habitus, suboccular calus and profile of frons illustrated by Lindberg (1948) [38] is the same as in the specimens identified as *T. fusca* by Dlabola and dissected from the MNHN collection, both do not correspond to Mitjaev’s interpretation. These specimens are morphologically distinct and we follow here Dlabola’s concept of *fusca* as the reference for the 1865’s Fieber taxon, and place it within *Mediodentometra*.

**4. Genus *Persiametra* Mozaffarian & Bourgoin,** ***gen. nov.***

urn:lsid:zoobank.org:act:D254995E-4894-4502-B7E0-103F11220B7D

(Figure 5f)

**Type species.** *Tettigometra pantherina* Horváth, 1891, here designated.

⁠

**Etymology.** The generic name *Persiametra* is derived from Persia, the classical Latin name for the region corresponding to modern-day Iran, where the genus is endemic along with some adjacent areas, and the suffix*-metra*, borrowed from the type genus *Tettigometra*. The name reflects both the restricted geographic distribution of the genus and its morphological and phylogenetic affinity with *Tettigometra* and related genera within the tribe Tettigometrini. The genus name is feminine in gender.

⁠

**Taxonomic status.** *Persiametra* ***gen. nov.*** is established here as a valid genus within Tettigometrini, based on the distinctive morphology of its type species, *T. pantherina*.

**Nomenclatural status.** Name newly proposed and available under ICZN Art. 13.1, with type species fixed by present designation.

⁠

**Diagnosis.** As in all species of the tettigometrinan group, the ventral process of the anal tube is positioned approximately at mid-length along the ventral margin in lateral view (Figure 4b). While the general habitus of the unique species of this genus allows a quick identification, it differs from all other taxa of the group by noticeably longer ventral process of anal tube (Figure 4b) and the absence of the mediodorsal aedeagal process. The ventral margin of the periandrium is rather short. Another characteristic of possible generic value is the elongate prefemora, distinctly visible in dorsal view and surpassing the pronotum.

⁠

**Included species in its new combination.** *Persiametra pantherina* (Horváth, 1891) ***comb. nov.***

⁠

**Geographical distribution:** Western Asia [Azerbaijan, Turkey], Southern Asia [Afghanistan, Iran] (Horváth, 1891 [75]; Nast, 1972 [52]; Dlabola, 1957 [67]; Mozaffarian et al., 2018 [1]; Bourgoin, 2025 [2]; Schlitt et al., 2025 [76]).

⁠

**Specimens studied:**

- ***Persiametra pantherina* (Horváth, 1891) *comb. nov.***

*Tettigometra pantherina* Horváth, 1891 (original combination)

→*Persiametra pantherina* (Horváth, 1891) ***comb. nov.*** (present transfer)

⁠

**Taxonomic status.** Valid species of *Persiametra*.

**Nomenclatural status.** Name available and valid (Horváth, 1891) [75].

⁠

**Illustrated.** Emeljanov (1980, based on non-type material).

**Identified specimens.** Dlabola, Mozaffarian (HMIM and MNHN).

**Dissected material.** Male genitalia examined in HMIM.

⁠

**Note.** The aedeagus illustration published by Emeljanov (1980) [29] confirm our dissections with lack of the mediodorsal aedeagal process (see Figure 1h). In contrast, Lindberg’s illustration of the male genitalia (1948: Figure 20C) [38] differs markedly from both Emeljanov’s (1980: Figure 3.2) [29], and from specimens of the Dlabola HMIM collection, or identified and examined by us. Re-examining Lindberg’s original material would be highly valuable to determine whether multiple species were involved. At present, it is not possible to assess which taxonomic concept corresponds most closely to Horváth’s original description. We hypothesize that Lindberg may have inadvertently illustrated a different species, due either to a dissection error or a specimen mix-up. The genital structures he figured correspond more closely to members of the newly described genus *Erratometra* ***gen. nov.*** (in apexometrinan group). Accordingly, we later record this concept as *Tettigometra pantherina* Horváth, 1891 *sec.* Lindberg, 1948 *nec* Horváth, in *Erratometra*, which may represent a previously unrecognized species.

⁠

**5. Genus *Mediodentometra* Bourgoin & Mozaffarian, *gen. nov.***

⁠

urn:lsid:zoobank.org:act:94D421B8-6A47-4D8C-8912-62CF0F107770

**Type species:** *Tettigometra sulphurea* Mulsant & Rey, 1855, here designated.

⁠

**Etymology.** Arbitrary combination of the Latin elements *medio-*(middle), *dento-*(tooth), and the suffix*-metra*, in reference to the genus *Tettigometra*, the type genus of the family. The name refers to the diagnostic presence of a ventral process of the anal tube in medial position. Gender: feminine.

⁠

**Taxonomic status.** *Mediodentometra* ***gen. nov.*** is here established as a valid genus within Tettigometrini, based on the distinct configuration of the mediodorsal aedeagal process and the submedial position of the anal ventral process.

⁠

**Nomenclatural status.** Name newly proposed and available under ICZN Art. 13.1, with type species fixed by present designation.

⁠

**Diagnosis.** A genus within the tettigometrinan group characterized by a ventral process of the anal tube positioned submedially (Figure 4a). The periandrium bears a lateral posterior margin that is more or less straight, becoming angularly concave, and joins a ventral margin at least twice as long as the mediodorsal aedeagal process. The latter is prominent, higher than width, and well-developed (Figure 4d), trapezoidal in shape; its anterior margin is often concave, making the process somewhat constricted; it is shaped as a roughly quadrangular base surmounted by a distinct triangular apex pointing anteriorly. The consistently well-developed, trapezoidal mediodorsal aedeagal process distinguishes this genus from all others within the tettigometrinan group (Figure 3), and particularly sets it apart from *Tettigometra s. str*. (Figure 4e–g), in which the mediodorsal aedeagal process lacks a quadrangular base and is only triangular in shape. From *Mimarada*, *Mediodentometra* ***gen. nov.*** differs by a higher-than-wide dorsal process versus lower-than-high in the former.

⁠

**Included species in their new combination:** *Mediodentometra beckeri* (Horváth, 1909) ***comb. nov.***, *Mediodentometra damryi* (Lethierry, 1876) ***comb. nov.***, *Mediodentometra impressopunctata* (Dufour, 1846) ***comb. nov.***, *Mediodentometra parihana* (Mozaffarian et Bourgoin, 2018) ***comb. nov.***, *Mediodentometra sulphurea* (Mulsant et Rey, 1855) ***comb. nov.***, *Mediodentometra vanai* (Dlabola, 1981) ***comb. nov.***, *Mediodentometra ventralis* (Signoret, 1866) ***comb. nov.***, and the problematic species *Mediodentometra fusca* (Fieber, 1865) ***comb. nov.*** *sec.* Dlabola (MMHN collections).

⁠

**Note.** Within this new genus, two morphological subdivisions appear to occur. In the first one (subdivision 1), the dorsal aedeagal process exhibits a wide base progressively joining the triangular upper part of it, as in the species *beckeri sec.* Lindberg, 1948, *sec.* Logvinenko, 1975 and *fusca* Fieber *sec.* Dlabola; in the second (subdivision 2), the dorsal aedeagal process shows a base constricted with a distinct concave basal posterior margin joining a dorsally more rounded process, wider than its base. However morphological variations might occur, and study of larger series of these taxa will be necessary before formalizing further these subdivisions.

⁠

**Geographical distribution:** Northern Africa [Algeria, Morocco, Tunisia], Southern Europe [Albania, Greece, Italy, Macedonia, Portugal, Serbia, Slovenia, Spain], Western Europe [Austria, Belgium, France, Germany, Switzerland], Northern Europe [Great Britain (England, Wales)], Eastern Europe [Czech Republic, European Turkey, Hungary, Moldova, Poland, Romania, Slovakia, Ukraine, Western Asia (Azerbaijan, Cyprus, Georgia, Jordan, Turkey), Southern Asia [Afghanistan, Iran], Central Asia [Kazakhstan, Kirghizia, Turkmenistan] (Fieber, 1865 [34]; Buckton, 1890 [77]; Horváth, 1897a [61]; Lindberg, 1949 [78], 1963 [79]; Jankovic, 1962 [80], 1966 [49], 1971 [50], Dlabola, 1965a [81]; Mitjaev, 1971 [64], 2002 [82]; Nast, 1972 [52]; Logvienko, 1975 [66]; Dlabola, 1981 [83]; Holzinger & Seljak, 2001 [84], Nickel, 2003 [85]; Demir, 2006 [65]; Oromi et al., 2010 [86]; Mozaffarian & Wilson, 2011 [69]; Swierczewski & Stroinski, 2011 [87]; Collgros & Lebecque, 2012 [88]; Gebicki et al., 2013 [55]; Mozaffarian et al., 2018 [1]; Gnezdilov, 2022 [89]; Seljak, 2023 [90], Bourgoin, 2025 [2]).

⁠

**Specimens studied:**

**- Subdivision 1:**

⁠

The two species included in this group present considerable difficulties in identification, either because of potential morphological variation that still requires evaluation through more rigorous molecular evidence (e.g., DNA barcoding), or owing to markedly divergent interpretations of their respective taxon concepts. Consequently, their current circumscription and the names proposed herein should be regarded as provisional and subject to future reassessment.

⁠

- ***Mediodentometra beckeri*** (Horváth, 1909) ***comb. nov.***

*Tettigometra beckeri* Horváth, 1909 (original combination)

→*Mediodentometra beckeri* (Horváth, 1909) ***comb. nov.*** (present transfer)

⁠

**Taxonomic status.** Valid species of *Mediodentometra* (subdivision 1).

**Nomenclatural status.** Name available and valid (Horváth, 1909).

⁠

**Illustrated.** Lindberg (1948) [38]; Logvinenko (1975) [66] (based on non-type material). Possibly Vilbaste (1971) [91] as *Tettigometra atrata*.

**Identified specimens.** Dlabola (MNHN coll.).

**Dissected material.** Male genitalia examined from the Dlabola collection (MNHN).

⁠

**Note.** The specimens identified by Dlabola as *T. beckeri* do not match the illustrations provided by Lindberg (1948: Figure 18B) [38] or Logvinenko (1975) [66], nor those of Mitjaev (1971: Figures 18, 21–24) [64]. Although the habitus of *T. beckeri* according to Dlabola’s identification and Lindberg’s figures appear similar, the specimens dissected during the present revision and previously identified as *T. beckeri* by Dlabola actually belong to the second division, lacking the wide-based dorsal process.

According to the taxon concepts of Lindberg [38] and Logvinenko [66], *T. beckeri* Horváth, 1909 *sec.* Mitjaev, 1971 *nec auth.* [64] represents a misidentification, probably corresponding to another species of *Mimarada* or to an undescribed taxon within that genus (see above).

Finally, the species illustrated by Vilbaste (1971: Figure 38c) [91] as *Tettigometra atrata* does not correspond to the *atra-atrata-depressa* concept discussed further (shape of aedeagal process, periandrium and anal tube conformations) and is here regarded as belonging to the *Mediodentometra* subdivision 1 concept as proposed in this study, probably to *Mediodentometra beckeri* (Horváth, 1909) ***comb. nov.***

Further dissections of series from various localities are needed to assess the extent and significance of morphological variation in this species.

⁠

- ***Mediodentometra fusca*** (Fieber, 1865) ***comb. nov.***

*Tettigometra fusca* Fieber, 1865 (original combination)

→*Mediodentometra fusca* (Fieber, 1865) ***comb. nov.*** (present transfer)

⁠

**Taxonomic status.** Tentatively placed in *Mediodentometra* subdivision 1, following Dlabola’s concept; identity remains uncertain pending examination of type material.

**Nomenclatural status.** Name available and valid (Fieber, 1865).

⁠

**Illustrated.** No illustration of male genitalia available for the true *fusca*.

**Identified specimens.** Dlabola (MNHN coll.).

**Dissected material.** Male genitalia examined from the Dlabola collection (MHNH).

⁠

**Note.** We follow here Dlabola’s interpretation of *T. fusca*, which is closer to Lindberg (1948) [38] regarding the habiuts and suboccular calus but differs markedly from Mitjaev (1971) [64]. The specimens illustrated by Mitjaev (1971: Figures 4–7 and 18) [64], treated above as *Mimarada* sp., likely represent an undescribed taxon distinct from Fieber’s species. Because the type specimen of *T. fusca* has not been examined, it remains uncertain which concept truly corresponds to Fieber’s original description. This taxonomic decision should be revisited in the future, ideally supported by molecular data from topotypic material.

⁠

**- Subdivision 2:**

⁠

In all species of this second group, the mediodorsal aedeagal process is strongly developed and distinctly higher than wide. The base of the process is not broad, as observed in subdivision 1, but its very presence allows a quick distinction from the species of the genus *Tettigometra s. str.*, in which such a base of the process is absent.

- ***Mediodentometra damryi*** (Lethierry, 1876) ***comb. nov.***

*Tettigometra damryi* Lethierry, 1876 (original combination)

→*Mediodentometra damryi* (Lethierry, 1876) ***comb. nov.*** (present transfer)

⁠

**Taxonomic status.** Valid species of *Mediodentometra* (subdivision 2).

**Nomenclatural status.** Name available and valid (Lethierry, 1876) [63].

⁠

**Identified specimens.** Noualhier, Lethierry, Puton (MNHN coll.).

**Dissected material.** Male genitalia examined from the Puton collection (MHNH).

- ***Mediodentometra impressopunctata*** (Dufour, 1846) ***comb. nov.***

*Tettigometra impresso-punctata* Dufour, 1846 (original combination)

=*Tettigometra frontalis* Fieber, 1865 (syn. by Fieber, 1872b: 2)

=*Tettigometra nitidula* Kirschbaum, 1868 (syn. by Fieber, Fieber, 1872b: 2)

→*Mediodentometra impressopunctata* (Dufour, 1846) ***comb. nov.*** (present transfer)

⁠

**Taxonomic status.** Valid species of *Mediodentometra* (subdivision 2).

**Nomenclatural status.** Name available and valid (Dufour, 1846) [92], with two junior synonyms proposed by Fieber 1872 [59,60].

⁠

**Identified specimens.** Dlabola, Noualhier–Lethierry, Oshanin, Perrier (MNHN coll.).

Dissected material. Male genitalia examined from the Dlabola collection (MNHN).

⁠

- ***Mediodentometra parihana*** (Mozaffarian & Bourgoin, 2018) ***comb. nov.***

*Tettigometra parihana* Mozaffarian & Bourgoin, 2018 (original combination)

→*Mediodentometra parihana* (Mozaffarian & Bourgoin, 2018) ***comb. nov.*** (present transfer)

⁠

**Taxonomic status.** Valid species of *Mediodentometra* (subdivision 2).

**Nomenclatural status.** Name available and valid (Mozaffarian et al., 2018) [1].

⁠

**Illustrated.** Mozaffarian & Bourgoin (2018, type material).

**Identified specimens.** Mozaffarian & Bourgoin (HMIM coll., type series).

**Dissected material.** Male genitalia examined in HMIM.

⁠

- ***Mediodentometra sulphurea*** (Mulsant & Rey, 1855) ***comb. nov.***

(Figure 5d)

*Tettigometra sulphurea* Mulsant & Rey, 1855 (original combination)

→*Mediodentometra sulphurea* (Mulsant & Rey, 1855) ***comb. nov.*** (present transfer)

⁠

**Taxonomic status.** Valid species of *Mediodentometra* (subdivision 1); type species of the genus.

**Nomenclatural status.** Name available and valid (Mulsant & Rey, 1855).

⁠

**Illustrated.** Lindberg (1948) [38]; Logvinenko (1975, Figure 219.3) [66]; Bourgoin (1988b) [43], all based on non-type material.

**Identified specimens.** Dlabola, Mozaffarian (HMIM coll.); Bergevin, Dlabola, Puton, Ribaut (MNHN coll.).

**Dissected material.** Male genitalia examined from the Dlabola collection) (MNHN, Figure 4d).

⁠

- ***Mediodentometra vanai*** (Dlabola, 1981) ***comb. nov.***

*Tettigometra vanai* Dlabola, 1981 (original combination)

→*Mediodentometra vanai* (Dlabola, 1981) ***comb. nov.*** (present transfer)

⁠

**Taxonomic status.** Valid species of *Mediodentometra* (subdivision 1).

**Nomenclatural status.** Name available and valid (Dlabola, 1981) [83].

⁠

**Illustrated.** Dlabola (1981, based on type material).

**Identified specimens.** No specimens examined.

⁠

- ***Mediodentometra ventralis*** (Signoret, 1866) ***comb. nov.***

*Tettigometra ventralis* Signoret, 1866 (original combination)

→*Mediodentometra ventralis* (Signoret, 1866) ***comb. nov.*** (present transfer)

⁠

**Taxonomic status.** Valid species of *Mediodentometra* (subdivision 2).

**Nomenclatural status.** Name available and valid (Signoret, 1866) [35].

⁠

**Identified specimens.** Noualhier–Lethierry, Puton (MNHN coll.).

**Dissected material.** Male genitalia examined from the Puton collection (MNHN).

⁠

**6. Genus *Tettigometra* Latreille, 1804, s.s.**

⁠

**Type species:** *Fulgora virescens* Panzer, 1799 by subsequent designation Kirkaldy, 1900: 264 [45].

⁠

**Taxonomic status.** *Tettigometra* Latreille, 1804 is here restricted to its nominotypical group (*sensu stricto*), grouping species characterized by a distinctly triangular mediodorsal aedeagal process without a distinct base (Figure 4e–g), with paired submedian ventral anal processes.

**Nomenclatural status.** Name available and valid (Latreille, 1804) [93]. Type species fixed by subsequent designation (*Fulgora virescens* Panzer, 1799 by Kirkaldy, 1900) [45]. *Tettigometra* is also the type genus of the tribe Tettigometrini. Gender: feminine.

⁠

**Diagnosis.** Species of *Tettigometra* s.s. exhibit a well-developed dorsal aedeagal process (Figure 4e–g), distinctly triangular and without distinct base in lateral view (vs. quadrangular-based in *Mediodentometra*, *Mimarada*, *Metroplaca*, and *Brachyceps*, or minute/almost absent in *Persiametra*) (Figure 3). As in other genera of the tettigometrinan group, the paired ventral processes of the anal tube are positioned near the mid-length of the tube in lateral view. The periandrium shows a lateral posterior margin that is nearly straight, becoming angularly more or less concave in the ventral third before merging with the ventral margin, which is at least twice as long as the mediodorsal aedeagal process.

⁠

**Note.** This group includes *Fulgora virescens* Panzer, 1799, the type species of *Tettigometra* as subsequently designated by Kirkaldy (1900: 264) [45]. The consistently triangular shape of the mediodorsal aedeagal process defines *Tettigometra* s.s. and distinguishes it from related genera within Tettigometrini.

⁠

**Included species.** *Tettigometra atra* Hagenbach, 1825; *Tettigometra atrata* Fieber, 1872 *Tettigometra brunnea* Signoret, 1866; *Tettigometra burjata* Kusnezov, 1929; *Tettigometra depressa* Fieber, 1865; *Tettigometra distincta* (Lucas, 1849); *Tettigometra virescens* (Panzer, 1799).

⁠

**Geographical distribution:** Northern Africa [Algeria, Libya, Morocco, Tunisia], Southern Europe [Greece, Italy, Malta, Portugal, Serbia, Spain], Western Europe [Austria, Belgium, France, Germany, Luxembourg, Netherlands, Switzerland], Northern Europe [Estonia Poland, Romania], Eastern Europe [Czech Republic, Hungary, Latvia, Slovakia, Ukraine], Western Asia [Armenia, Azerbaijan, Turkey], Southern Asia [Iran], Central Asia [Kazakhstan, Uzbekistan], Northern Asia [Russian Far East, Siberia], Eastern Asia [China, Mongolia], Southeastern Asia [Maritime Territory] (Mozaffarian et al., 2018 [1]; Bourgoin, 2025 [2]; Lindberg, 1948 [38]; Fokker, 1900 [48]; Jankovic, 1966 [49], 1971 [50]; Nast, 1972 [52]; Gebicki et al., 2013 [55]; Albre & Gibernau, 2019 [56]; D’Urso et al., 2019 [57]; Den Bieman et al., 2020 [58]; Logvinenko (1975) [66]; Anufriev & Emeljanov, 1988 [68]; Mozaffarian & Wilson, 2011 [69]; Lindberg, 1949 [78], 1963 [79]; Nickel, 2003 [85]; Collgros & Lebecque, 2012 [88]; Gnezdilov, 2022 [89]; Seljak, 2023 [90]; Haupt, 1917 [94]; Linnavuori, 1965 [95]; Dlabola, 1965b [96], 1970 [97]; Anufriev, 1969 [98]; Emeljanov, 1982 [99]; Holzinger et al., 2020 [100]).

⁠

**Specimens studied:**

- ***Tettigometra atra*** **Hagenbach, 1825**

=*Tettigometra piceola* Burmeister, 1835 (syn. by Fieber, 1872b: 2)

⁠

**Taxonomic status.** Provisionally maintained as a valid species of *Tettigometra* s.s., pending clarification of its distinction from *T. atrata* Fieber, 1872 and *T. depressa* Fieber, 1865 (see *atra/atrata/depressa* issue below).

**Nomenclatural status.** Name available and valid (Hagenbach, 1825) [101]. The original description was not available to Metcalf (1932: 16) [37], who flagged it as “not checked”. The first subsequent mention and acceptance of the name appeared in Herrich-Schäffer (1835: 2) [62], confirming its usage. *T. atra* was firstly synonymized with *T. piceola* Burmeister, 1835 by Fieber (1865: 565) [34] then correctly reversed in 1872b: 2 [60].

⁠

**Illustrated.** Lindberg (1948) [38]; Logvinenko (1975) [66], both based on non-type material.

**Identified specimens.** Bergevin, Dlabola, Noualhier–Lethierry, Oshanin, Perrier, Puton, and Ribaut (MNHN collections).

**Dissected material.** Male genitalia examined from the Dlabola collection (MNHN).

⁠

- ***Tettigometra atrata*** **Fieber, 1872**

=*Tettigometra atra* Fieber 1865 *nec* Hagenbach, 1825 (syn. by Fieber 1872b: 2 [60])

⁠

**Taxonomic status.** Provisionally maintained as a distinct species of *Tettigometra s.s.*, though possibly conspecific with *T. atra* Hagenbach, 1825 and/or *T. depressa* Fieber, 1865 (see issue note, further).

**Nomenclatural status.** Name available and valid (Fieber, 1872a) [59]. Puton (1875) [102] and Lindberg (1948) [38] synonymized the species with *T. atra* Fieber *nec* Hagenbach, 1825 following Fieber 1872b [60], therefore leaving *T. atra* Hagenbach, 1825, *nec* Fieber, 1865 valid. Holzinger (2009) [103] considered *T. atrata* Fieber, 1872 as a synonym of *T. atra* Hagenbach, 1825, but without justification.

⁠

**Illustrated.** Lindberg (1948) [38], non-type material.

**Identified specimens.** Dlabola, Perrier and Ribaut (MNHN collections).

**Dissected material.** Male genitalia examined from the Dlabola collection (MNHN).

⁠

**Note.** The specimen illustrated by Vilbaste (1971) [91] differs markedly from Lindberg 1948’s concept [38]. Its distinct wide base of the dorsal process places it into the new genus *Mediodentometra* ***gen. nov.*** (see above), and might represent a misidentified specimen of *M. beckeri* (Horváth, 1909) ***comb. nov.*** as here recognized (see above).

⁠

- ***Tettigometra depressa*** **Fieber, 1865**

=*Tettigometra atrata* Haupt, 1935 *nec* Fieber, 1872 (syn. by Lindberg, 1948 [38])

⁠

**Taxonomic status.** Provisionally maintained as a distinct species of *Tettigometra* s.s., though possibly conspecific with *T. atra* Hagenbach, 1825 and/or *T. atrata* Fieber, 1872 (see issue note).

**Nomenclatural status.** Name available and valid (Fieber, 1865) [34]. Junior synonym: *T. atrata* Haupt, 1935 *nec* Fieber, 1872 (synonymized by Lindberg, 1948 [38]). Holzinger (2009) [103] mentioned *T. depressa* as a junior synonym of *T. atra* Hagenbach, 1825, but the reference or justification for this synonymy was not provided.

⁠

**Illustrated.** Lindberg (1948) [38]; Logvinenko (1975) [66], both based on non-type material.

**Identified specimens.** Dlabola, Puton (MNHN collections).

**Dissected material.** Male genitalia examined from the Dlabola collection (MNHN).

⁠

**Note. The *atra*/*atrata*/*depressa* issue.** All synonymies discussed here are subjective, as they involve distinct name-bearing types and their validity therefore depends on the taxonomic interpretation adopted by successive authors. A critical review of the literature and of all available specimens identified under the names *Tettigometra atra*, *T. atrata*, and *T. depressa* in the various collections has not allowed us to distinguish these taxa in a consistent manner. Variations in coloration, the degree of anterior projection of the vertex (slightly projected or more rounded), and the posterior tapering of the tegmina (slightly acute or more rounded) were observed, but these features might be continuous variations and strongly dependent on the viewing angle adopted by the observer. However, observations based on series of specimens and from different localities are still necessary and lacking to evaluate whether these subtle morphological differences reflect true species boundaries or intraspecific variability.

⁠

Holzinger et al. (2003) [28] mentioned the three taxa as conspecific. Although he mentionned that “in his view he did not formally synonymize the three species” (pers. com.), the synonymy these authors attributed to Lindberg [38] should therefore be credited to Holzinger et al. (2003) [28] under ICZN authorship conventions. Our examination of male genitalia likewise raised a similar conclusion, failing to reveal consistent diagnostic differences. Instead, all specimens shared two common characters: an anal tube with a slightly but distinctly concave dorsal margin, and a ventral margin sinuated by a slight convex swelling. These characters separate the assemblage from other *Tettigometra* s.s., in particular, from the type species *T. virescens*.

⁠

Although the synonmy of these three species is probable, pending molecular evidence, it is not possible at this stage to determine whether the material represents a single polymorphic species or several distinct but closely related species. Accordingly, we provisionally maintain the three species taxa concept names (*atra*, *atrata*, *depressa*) in current usage, although the possibility remains that only one or more valid biological entities are involved.

⁠

Finally, the specimen figured by Vilbaste (1971) [91] as *T. atra* clearly displays a different mediodorsal aedeagal process conformation with a rectangular base, placing it within *Mediodentometra*, highlighting the persistent confusion surrounding these taxa and their names.

⁠

- ***Tettigometra picea*** **Kirschbaum, 1865**

*Tettigometra picea* Kirschbaum, 1868: 58 [104]. (original comb.)

≠*Tettigometra brunnea* Signoret, 1866 (err. syn. by Puton, 1899, rejected herein, treated as species incertae sedis; see below).

≠*Tettigometra afra* Kirschbaum, 1868 (err. syn. by Horváth, 1912, rejected herein, treated as distinct in *Eurychila **stat. nov.***; see below).

⁠

**Taxonomic status.** Valid species of *Tettigometra s. str*.

Nomenclatural status. Name available and valid (Kirschbaum, 1868) [104].

⁠

**Illustrated.** No illustrations available.

**Identified and dissected specimen.** One specimen from Puton’s collection previously identified as *T. brunnea* and synonymized with *T. picea* by Puton 1899 [105]

⁠

**Additional diagnosis**. Body slender, slightly smaller and narrower than *afra*. Coloration dark brown to piceous; pronotum smooth and slightly shiny; tegmina narrow, elongate, with a faint but distinct costal groove. Frons bearing a pale whitish basal band, slightly convex in lateral view. Aedeagus with a distinctly elongate, antero-dorsally directed triangular dorsal process (Figure 4g).

⁠

**Note.** *T. picea* was first synonymized with *T. brunnea* by Puton (1899) [105], but Horváth (1912) [106] restored its validity based on habitus and tegmen morphology. Examination of Puton’s material confirms *picea* as a Sicilian distinct species distinct from *afra* Kirschbaum, 1868 and probably *brunnea* Signoret (1866) from Algeria [35]. The aedeagal structure supports its placement in the tettigometrinan group, while *afra* belongs to the apexometrinan one.

⁠

**Note. The *picea*/*afra*/*brunnea* issue.** The long-standing confusion surrounding *Tettigometra brunnea* Signoret, 1866, *T. afra* Kirschbaum, 1868, and *T. picea* Kirschbaum, 1865 results from synonymies proposed by Puton (1899) [105] and Horváth (1912) [106], for which no explanation was provided. None of these synonymies relied on male genital features, which are essential for reliable species delimitation within the group.

⁠

Our re-examination of historical specimens from the Puton, and Noualhier–Lethierry collections, as well as from Dlabola’s material identified as *brunnea*, reveals the presence of four morphologically distinct species. To properly associate these taxa with their corresponding names, the following facts must be considered:

- A series of specimens in the Puton collection is labeled “*brunnea = picea*”. They most likely represent the individuals on which Puton (1899) [105] based his synonymy, treating *picea* as a synonym of *brunnea*. Within his collection, two distinct specific forms can be recognized: one corresponding to *picea* as defined by Horváth (1912) [106], and another that we tentatively recognize as the true *brunnea* form. Dissection of the former places *picea* within *Tettigometra* s.s., characterized by a distinctly elongate, anteriorly and dorsally directed triangular dorsal aedeagal process (Figure 4g). The latter differs clearly from *T. afra* Kirschbaum (1868) by its less convex pronotum, paler coloration, shorter tegmina with a weaker costal groove, shinier pronotum, and a pale whitish basal band on the frons, which is distinctly absent in *afra*. This specimen, however, is a female and was not dissected; its taxonomic position therefore remains uncertain (*incertae sedis*) (see part IV of the taxonomic results).
- Horváth (1912) [106] regarded *picea* as a valid species, basing his conclusion on several external morphological characters that we confirm here plus genital features: a thinner overall habitus, tegmina with a shallow costal groove, a whitish band at the base of the frons, slightly convex in lateral view, and the characteristic dorsal aedeagal process (Figure 4g).
- While Horváth (1912) [106] listed *afra* as a new synonym of *brunnea* (without justification); specimens of *afra* from both the Puton, and Noualhier–Lethierry collections correspond to the same species concept, which in all three cases differs from the true *brunnea* concept of the Signoret *sec.* Puton collection. This third species belongs to the genus *Eurychila* ***stat. nov.*** within the apexometrinan group, and is treated above as a valid distinct species.
- A fourth form, *brunnea sec.* Dlabola coll., previously included under the general *brunnea* concept according to Dlabola identified specimens in MNHN collections, also belongs to the apexometrinan group, but within the genus *Stirometra* as defined in the present revision, and is listed below as a distinct species.

⁠

At this stage, we therefore observe four distinct morphological species concepts for which only three distinct taxon-concepts have been described, each associated with a valid name, while two of these taxa remain currently accepted. At present, none of the historical taxon-concepts can be confidently linked to their original name, and the synonymies proposed by both Puton (1899) [105] and Horváth (1912) [106] remaining without justification cannot be confirmed. In the absence of type material, illustrations of male genitalia, or reliable diagnostic evidence linking names to species concepts, the most conservative and defensible approach is to treat all taxa as provisionally valid. We therefore cautiously maintain the different described species as valid (based on Horváth’s (1912) [106] definition of *T. picea* Kirshbaum, 1868 with the additional characters observed here for *T. brunnea* Signoret (1866) and *E. afra* (Kirschbaum, 1868) and reject the synonymies proposed by Puton (1899) [105] and Horváth (1912) [106]. We also recognize, in addition, one clearly distinct species now placed in *Stirometra*. This interpretation preserves nomenclatural stability while leaving open the possibility of future synonymization once the type material and additional populations can be reassessed through both morphological and molecular analyses.

⁠

Based on indirect locality evidence, the Algerian material most likely represents the true *brunnea* described by Signoret 1866 [35], whereas the Sicilian material (Kirschbaum, 1868 [104], Horvath (1912) [106]) corresponds to different species from the apexometrinan group.

⁠

- ***Tettigometra burjata*** Kusnezov, 1929

=*Tettigometra picicolor* Lindberg, 1948 (syn. by Emeljanov, 1977)

=*Tettigometra mongolica* Lindberg, 1948 (syn. by Emeljanov, 1977)

⁠

**Taxonomic status.** Valid species of *Tettigometra* s.s.

**Nomenclatural status.** Name available and valid (Kusnezov, 1929) [107]. Junior synonyms: *T. picicolor* Lindberg, 1948; *T. mongolica* Lindberg, 1948 (both synonymized with *T. burjata* by Emeljanov, 1977 [74]).

⁠

**Illustrated.** Lindberg (1948: type material of *T. picicolor* and *T. mongolica*) [38]; Mitjaev (1971: non-type material of *T. mongolica*) [64]; Anufriev & Emeljanov (1988: non-type material of *T. burjata*) [68].

**Identified specimens.** *T. mongolica* Lindberg, 1948 identified by Dlabola (MNHN collections).

**Dissected material.** Male genitalia examined from the Dlabola collection (MNHN).

⁠

- ***Tettigometra distincta*** (Lucas, 1849)

*Ptyleus distincta lucas*, 1849 (original combination)

→*Tettigometra distincta* (Lucas, 1849) (tranferred by Signoret, 1866: 153)

⁠

**Taxonomic status.** Valid species of *Tettigometra* s.s.

**Nomenclatural status.** Name available and valid (Lucas, 1849) [108].

⁠

**Illustrated.** Lindberg (1948) [38], based on non-type material.

**Identified specimens.** Bergevin, Dlabola (MNHN).

**Dissected material.** Male genitalia examined from the Dlabola collection (MNHN, Figure 4f).

⁠

- ***Tettigometra virescens*** (Panzer, 1799) (nomen protectum)

Figure 5e

*Fulgora virescens* Panzer, 1799 (original combination, *nomen protectum*)

=*Cicada acephala* Fourcroy, 1785 *(nomen oblitum)* (syn. by Puton, 1886: 75; reiterated 1899)

→*Tettigometra virescens* (Panzer, 1799*)* (transferred by Latreille, 1804: 312)

=*Tettigometra dorsalis* Latreille, 1804 (syn. by Puton, 1886: 75)

=*Tettigometra bicolor* Costa, 1834 (syn. by Amyot, 1847: 179)

=*Tettigometra brachynota* Fieber, 1865 (syn. by Holzinger, 2009: 55)

=*Tettigometra concolor* Fieber, 1865 (orig. as var. of *T. virescens*; raised to species by Horváth, 1898, syn. by Holzinger, 2009: 55)

=*Tettigometra sicula* Kirschbaum, 1868 (syn. by Fieber, 1872b: 2)

=*Tettigometra fuscipes* Fieber, 1876 (orig. as var. of *T. virescens*; raised to species by Horváth, 1903; syn. by Lindberg, 1948)

⁠

**Taxonomic status.** Valid taxon. Type species of *Tettigometra s.s*.

**Nomenclatural status.** Name available and valid (Panzer, 1799) [109]. Type species fixed by subsequent designation (*Fulgora virescens* Panzer, 1799; Kirkaldy, 1900).

⁠

**Illustrated.** Lindberg (1948) [38]; Logvinenko (1975) [66]; Emeljanov (1980) [29], all based on non-type material.

**Identified specimens.** Bergevin, Dlabola, Noualhier–Lethierry, Perrier, Ribaut (MNHN collections); Mozaffarian (NMWC).

**Dissected material.** Male genitalia examined from the Dlabola collection (MNHN) and NMWC collections.

*-* var*. bicolor* Costa, 1834

**Identified specimens.** Noualhier–Lethierry, Oshanin, Puton, Ribaut (MNHN coll.); Mozaffarian (NMWC).

**Dissected material.** Male genitalia examined from the specimen of NMWC.

*-* var*. dorsalis* Latreille, 1804

**Identified specimens.** Perrier, Puton (MNHN coll.); Mozaffarian (NMWC).

**Dissected material.** Male genitalia examined from the specimen of NMWC.

*-* var*. concolor* Fieber, 1865

**Identified specimens.** Perrier, Ribaut (MNHN coll.).

**Dissected material.** Male genitalia examined from the Ribaut collection (MNHN Figure 4e).

⁠

***Note.*** *Cicada acephala* Fourcroy, 1785 [110] (also cited in Geoffroy, 1799, [111]), was placed in synonymy with *Fulgora virescens* Panzer, 1799 (transferred to *Tettigometra* by Latreille, 1804) [93] by Puton (1886 [44], reiterated 1899 [105]), but has never been used as a valid name after 1899. According to ICZN Art. 23.9.1.1, the senior synonym (*acephala*) has not been used as valid after 1899, whereas the junior synonym (*virescens*) has been consistently employed in prevailing usage (ICZN Art. 23.9.1.2), with numerous citations by many authors over the past two centuries. Under ICZN Art. 23.9 (“Reversal of precedence”), *virescens* must therefore be maintained as the valid name (*nomen protectum*), and *acephala* treated as a nomen oblitum.

⁠

Several forms historically associated with *Tettigometra virescens* were either described as distinct species (*T. bicolor* Costa, 1834; *T. dorsalis* Latreille, 1804; *T. fuscipes* Horváth, 1897 = var. *concolor* Fieber, 1865, *fide* Oshanin, 1907; *T. sicula* Kirschbaum, 1868) or later raised to species rank (e.g., var. *hispanica* Fieber, 1872, elevated by Chicote, 1880). Others were introduced directly as infrasubspecific taxa (varieties), such as var. *brunnescens* Rey, 1894; var. *concolor* Fieber, 1872 = var. *confusa* Metcalf, 1932; var. *fasciata* Fieber, 1872; var. *grisescens* Amyot, 1847; var. *luteicollis* Rey, 1894; var. *luteiventris* Rey, 1894; var. *notaticollis* Rey, 1891; var. *sanguinolenta* Rey, 1894; var. *subgrisea* Rey, 1891; and var. *variegata* Rey, 1891. *T. brachynota* Fieber, 1865, also listed as valid in Metcalf (1932), is currently informally (pers. com.) treated as a synonym by Holzinger, 2009: 55 [103] followed by the Dmitriev website [112].

⁠

All these names, reproduced by Metcalf (1932) [37] as varieties, are unavailable under ICZN Articles 45.6.1 and 45.5.1 unless explicitly adopted at species rank by subsequent authors. Even those few temporarily considered as species were later treated as synonyms of *T. virescens* by Lindberg (1948) [38] or by Holzinger (2009) [103]. All these synonymic statements were again merely listed and followed in the nomenclator of Dmitriev et al. (2025) [112]. We did not re-examine all these forms and follow the interpretations of the above authors, who uniformly regard them as infrasubspecific or junior synonyms of *T. virescens.*

⁠

However, as in many other tettigometrid species of this revision, several synonymies historically attached to *Tettigometra virescens* were never explicitly justified. This is particularly the case for *T. bicolor* Costa, 1834 and *T. concolor* Fieber, 1865, for both of which the synonymies require confirmation, as doubts have been respectively expressed by Holzinger (pers. comm.) and Nickel (pers. comm.). In accordance with our conservative approach, and for the sake of nomenclatural stability, these names are provisionally retained as synonyms following Holzinger (2009) [103], pending future revision based on type material when possible, or, alternatively, on clearer molecular evidence.

⁠

**III. The apexometrinan group**

⁠

**Taxonomic status.** Phenetic, morphologically defined group within *Tettigometrini* Germar, 1821. It is used here as a practical framework to delimit genera sharing a common set of diagnostic morphological characters distinct from the tettigometrinan group. It is not treated as a formal taxonomic taxa and its potential recognition at subtribal rank remain to be tested with molecular phylogenetic data for its monophyly.

⁠

**Diagnosis.** Species assigned to the apexometrinan group are characterized by the apical to subapical position of the ventral process of the anal tube in lateral view (Figure 6a,b), located in the last quarter of its length. The process often arises as a smooth prolongation of the posterior margin of the anal tube, which curves ventrally before giving rise to the process, and the ventral margin begins only after the process or directly on the ventral margin of anal tube (Figure 6b), very close to the apex. This condition contrasts with that of the tettigometrinan group, where the process distinctly protrudes medially from the ventral margin and is never aligned with the posterior one (Figure 4a,b).

⁠

Within the apexometrinan group, generic delimitation is mainly supported by variation in the shape of the mediodorsal aedeagal process (absent, triangular, finger-like, or subrectangular, Figure 5c–g), most often apically rounded, together with the conformation of the periandrium and anal tube. When subrectangular, as in *Apexometra*, the process is weakly elevated, in contrast to the more strongly developed condition observed in the tettigometrinan genera *Mediodentometra **gen. nov.***, *Brachyceps* ***stat. nov.***, *Metroplaca* ***stat. nov.***, and *Mimarada* ***stat. nov.*** (Figure 3). The genera *Eurychila* ***stat. nov.***, *Erratometra* ***gen. nov.***, *Macrometrina* ***stat. nov.***, *Mitricephalus* ***stat. nov.*** and *Apexometra* ***gen. nov.*** show an anal ventral process in a little less apical position than other genera of the group *Stirometra* ***stat. nov.***, *Hystigonia* ***stat. nov.***, *and Micracanthometra* ***gen. nov.***, though not in the distinctly submedian position typical of tettigometrinan genera (Figure 3).

⁠

**Note.** The apexometrinan group is morphologically defined by a clear and unique apical to subapical positioning of the anal tube process. This striking feature, in combination with variation in anal tube and periandrium conformations and aedeagal structures, supports the grouping as a distinct and diagnosable assemblage. As with the tettigometrinan group of genera, its monophyly and possible recognition at a higher taxonomic rank as a subtribe remain to be tested through future molecular phylogenetic analyses, and it is therefore not formally described here.

⁠

**Included genera.** *Apexometra* Bourgoin & Mozaffarian, ***gen. nov.***; *Erratometra* Mozaffarian & Bourgoin, ***gen. nov.***; *Eurychila* Signoret 1866 ***stat. rev.***; *Hystrigonia* Emeljanov, 1980 ***stat. nov.***; *Macrometrina* Lindberg, 1948 ***stat. rev.***, *Micracanthometra* Mozaffarian & Bourgoin, ***gen. nov.***; *Mitricephalus* Signoret, 1866 ***stat. rev.***; *Stirometra* Emeljanov 1980 ***stat. nov.***

⁠

**1. Genus *Apexometra* Bourgoin & Mozaffarian, *gen. nov.***

urn:lsid:zoobank.org:act:FCC1F992-C4B9-459B-AB35-6E4E34934

⁠

**Type species:** *Tettigometra griseola* Fieber, 1865, here designated.

⁠

**Etymology.** Arbitrary combination of the Greek-derived element *apex-*(referring to the apical position) and the suffix*-metra*, referencing the genus *Tettigometra*, the type genus of the family. The name highlights the diagnostic presence of a ventral process of the anal tube in apical position. Gender: feminine.

⁠

**Taxonomic status.** *Apexometra* ***gen. nov.*** is here established as a valid genus within Tettigometrini, apexometrinan group, based on the distinct configuration of the mediodorsal aedeagal process and the submedial position of the anal ventral processes.

**Nomenclatural status.** Name newly proposed and available under ICZN Art. 13.1, with type species fixed by present designation.

⁠

**Diagnosis**. In addition to the character states of the apexometrinan group, the anal tube is shorter than high in lateral view. The ventral anal process is positioned more subapically than in *Stirometra* and *Hystrigonia*, arising as a smooth prolongation of the posterior margin turning obliquely ventral. The mediodorsal aedeagal process is quadrangular, distinctly longer than high, and oriented antero-dorsally. It is never triangular or finger-like, nor apically rounded. This condition contrasts with *Eurychila* ***stat. nov.*** (exhibiting only a swollen aedeagal dorsal margin), *Macrometrina* ***stat. nov.*** and *Mitricephalus* ***stat. nov.*** (with finger-like processes), and *Erratometra* ***gen. nov.*** (triangular to trapezoidal processes, as high as wide, apically rounded) (Figure 3).

⁠

**Included species.** *Apexometra griseola* (Fieber, 1865); *Apexometra sororcula* (Horváth, 1897).

⁠

**Geographical distribution:** Northern Africa [Algeria, Morocco], Southern Europe [Italy, Greece, Malta, Serbia, Spain], Western Europe [France], Eastern Europe [Hungary, Moldova, Romania], Western Asia [Azerbaijan, Jordan], Southern Asia [Iran], Central Asia [Kazakhstan] (Horváth, 1897b [113]; Lindberg, 1963 [79]; Dlabola, 1965a [81]; Linnavuori, 1965 [95]; Jankovic, 1971b [114]; Nast, 1972 [52]; Mozaffarian & Wilson, 2011 [69]; Mozaffarian et al., 2018 [1]; Albre & Gibernau, 2019 [56]; D’Urso et al., 2019 [57]; Bourgoin, 2025 [2]).

⁠

**Specimens studied:**

- ***Apexometra*** ***griseola*** **Fieber, 1865, *comb. nov.***

(Figure 7d,e)

*Tettigometra griseola* Fieber, 1865 (original combination)

=*Tettigometra scutellata* Signoret 1866 (syn. by Puton, 1886: 75)

=*Tettigometra umbrosa* Signoret 1866 (syn. by Puton, 1886: 75)

→*Apexometra griseola* (Fieber, 1865), ***comb. nov.*** (present transfer)

⁠

**Taxonomic status.** Valid species of *Apexometra*.

**Nomenclatural status.** Name available and valid (Fieber, 1865) [34] for the type species of the genus. *T. bimaculata* was originally described by Signoret (1866) [35] as a variety of *T. griseola*. It was consistently treated at varietal rank by subsequent authors (e.g., Puton, 1886 [44]; Lindberg, 1948 [38]; Metcalf, 1932) [37]. Nast (1972) [52] unusually reversed the relationship, synonymizing the taxon *T. griseola* under *T. bimaculata*, but this interpretation has not been followed and remains unsupported.

⁠

**Illustrated.** Lindberg, 1948 [38]; Mitjaev, 1971 [64]; Logvinenko, 1975 [66] (all based on non-type material).

**Identified specimens.** Noualhier–Lethierry, Perrier, Puton, and Ribaut (MNHN)

**Dissected material.** Male genitalia examined from the Noualhier–Lethierry, Perrier and Puton collections (MNHN).

***-* var. *frontalis* Rey, 1894** (= var. *frontifera* nom. nov. Metcalf, 1932: 28)

**Identified specimens.** Perrier (MNHN).

**Dissected material.** Male genitalia examined from the Perrier collection (MNHN).

⁠

- ***Apexometra sororcula* (Horváth, 1897), *comb. nov.***

(Figure 7c)

*Tettigometra sororcula* Horváth, 1897 (original combination)

→*Apexometra sororcula* (Horváth, 1897), ***comb. nov.*** (present transfer)

⁠

**Taxonomic status.** Valid species of *Apexometra*.

**Nomenclatural status.** Name available and valid (Horváth, 1897b) [113]. The species was historically confused with *Tettigometra griseola* due to mislabeling in the Dlabola and Noualhier–Lethierry collections, but examination of male genitalia (after Ribaut, Perrier, and Dlabola determinations) confirms its distinctness and justifies its transfer to *Apexometra*.

⁠

**Illustrated.** No illustration of the male genitalia is known for this species.

**Identified specimens.** Dlabola, in part (*T. griseola* Fieber, 1865 *sec.* Malenovsky, misidentification), Perrier, Ribaut (MNHN collections).

**Dissected material.** Male genitalia examined from the Dlabola collection (MNHN).

⁠

**Note.** The species has been confused with *T. griseola* by some authors (e.g., *sec.* Malenovsky *apud* Dlabola, Holzinger et al. (2003) [26], and Kunz et al. (2011) [115]), but examination of male genitalia confirms its distinction. While no published illustration of the male genitalia of *sororcula* is known, the present identification follows the determinations made by Perrier and Ribaut, whose material agrees with part of the specimens identified as *Tettigometra sororcula* by Dlabola (the remaining specimens in his series being females and probably misidentified). Male genitalia were prepared and examined from these specimens and are here considered representative of *Apexometra sororcula* (Horváth, 1897). Accordingly, the two species can be separated by confluent white spots forming a distinct humeral white patch in the tegmina of *A. sororcula*, absent or indistinct and with dispersed spots in *A. griseola*: and by the anal tube ventral margin distinctly longer in the former than in the latter, and the mediodorsal aeadeagal process dorsal margin longer than high and somewhat concave in *A. sororcula*, versus shorter and straight in *A. griseola*.

⁠

Malenovsky subsequently added labels to Dlabola’s *sororcula* specimens identifying them as *T. griseola*, probably following Holzinger et al. (2003) [28] and Kunz et al. (2011) [115], who illustrated a similar habitus under that name. Some white patching is present in *T. griseola* var. *livida sec.* Puton but subtly different, still less distinct, with more dispersed spots.

⁠

In the Noualhier–Lethierry collection, all specimens identified as *T. griseola* correspond to the typical *griseola* form as found in other collections, except for a single female exhibiting the habitus of *sororcula sensu* Ribaut, Perrier, and Dlabola (equivalent to *griseola sensu* Holzinger, Malenovsky, and Kunz). This single female was later selected as the type specimen of *T. griseola* by Kunz and moved to the Fieber type box in MNHN collections, which has further complicated the interpretation of both names.

⁠

Mitjaev’s 1971 illustration [64], probably for *T. griseola*, most probably refers to *A. sororcula **comb. nov.*** as here defined.

⁠

**Misidentification:**

- **Unidentified species**

Misidentified as *Tettigometra longicornis sec.* illust. Mitjaev, 1971 (Figures 18, 35–37).

⁠

**Taxonomic status.** Misidentified taxon belonging to the apexometrinan group. The illustration published by Mitjaev (1971) [64] under the name *Tettigometra longicornis* does not correspond to *Metroplaca longicornis* (Signoret, 1866) [35], but instead represents another species referable to this newly established genus *Apexometra*.

**Nomenclatural status.** The name *Tettigometra longicornis* is available and valid for the Signoret species only. The usage by Mitjaev (1971) [64] represents a misidentification and has no nomenclatural standing under the ICZN for this probable new taxon.

⁠

**Illustrated.** Mitjaev (1971, Figures 18, 35–37) [misidentification].

**Identified specimens.** Not confirmed; material illustrated by Mitjaev requires re-examination.

⁠

**Note.** Pending formal revision, this material is here treated as an unidentified species of *Apexometra **gen. nov.***, based on its anal tube being relatively short (slightly longer than high) and its mediodorsal aedeagal process close to the *A. sororcula* conformation, while the frons in profile is illustrated straight, being concave in *A. sororcula*.

⁠

**2. Genus *Erratometra* Mozaffarian & Bourgoin, *gen. nov.***

⁠

urn:lsid:zoobank.org:act:911250BA-497D-476C-A814-ABA6A8A25652

**Type species:** *Erratometra leucophaea* (Preyssler, 1792), here designated.

⁠

**Etymology.** The generic name *Erratometra* is derived an arbitrary combination of the Latin word *erratus* and the suffix *-metra*, referencing the genus *Tettigometra*, the type genus of the family. The name refers to the historical problematic and misidentification of species of this taxon. Gender: feminine.

⁠

**Taxonomic status.** *Erratometra* ***gen. nov.*** is established here as a valid genus within the apexometrinan group of Tettigometrini.

**Nomenclatural status.** Name newly proposed and available under ICZN Art. 13.1, with type species fixed by present designation.

⁠

**Diagnosis**. In addition to the character states of the apexometrinan group, the anal tube in lateral view is slightly longer than high; the ventral anal process is positioned in a more subapical location than in the genera *Stirometra* ***stat. nov.*** and *Hystrigonia* ***stat. nov.***, arising as a smooth prolongation of the posterior margin, which turns obliquely ventral (Figure 3). The dorsal aedeagal process is always strongly developed, apically rounded, generally as wide as high, triangular in shape (Figure 6f) to somewhat more trapezoidal and directed antero-dorsally when more developed. It differs in this respect from *Eurychila* ***stat. nov.***, which only shows a swollen dorsal aedeagal margin (Figure 6f,g); from *Mitricephalus* ***stat. nov.***, which exhibit a strong finger-like mediodorsal process with parallel margins (Figure 6e); and from *Apexometra* ***gen. nov.***, which exhibits a more rectangularly shaped process (Figure 3).

⁠

**Included species:** *Erratometra bifoveolata* (Signoret, 1866) ***comb. nov.***; *Erratometra eremi* (Lindberg, 1948) n. comb; *Erratometra leucophaea* (Preyssler, 1792) ***comb. nov.***; *Erratometra lyncea* (Horváth, 1903) ***comb. nov.***; *Erratometra sordida* (Fieber, 1865) ***comb. nov.***; *Erratometra stepposa* (Logvinenko, 1975) ***comb. nov.***, and the species taxa recorded as *Tettigometra pantherina* Horváth, 1891 *sec.* Lindberg (1948: 34,36) and *Tettigometra cerina* Lindberg, 1948, *sec.* Mitjaev 1971 Figures 14–17)

⁠

**Geographical distribution:** Northern Africa [Algeria, Morocco, Tunisia], Southern Europe [Albania, Italy, Serbia, Slovenia, Spain], Western Europe [Austria, Belgium, France, Germany, Netherlands, Norway], Eastern Europe [Bulgaria, Czech Republic, Hungary, Moldova, Poland, Romania, Slovakia, Ukraine], Western Asia [Armenia, Azerbaijan, Dagestan Georgia, Jordan, Israel, Syria, Turkey], Southern Asia [Afghanistan, Iran], Central Asia [Kazakhstan, Kirgizia, Tajikistan, Turkmenistan, Uzbeksitan], Northern Asia [Russian, Far East Siberia], Eastern Asia [Mongolia], (Mozaffarian et al., 2018 [1]; Bourgoin, 2025 [2]; Fokker, 1900 [48]; Jankovic, 1966 [49]; Nast, 1972 [52]; Demir, 2006 [65]; Dlabola, 1957 [67]; Anufriev & Emeljanov, 1988 [68]; Mozaffarian & Wilson, 2011 [69]; Lindberg, 1949 [78], 1963 [79]; Jankovic, 1962 [80]; Dlabola 1965a [81], Holzinger & Seljak, 2001 [84]; Linnavuori, 1965 [95]; Dlabola 1970 [97]; Emeljanov, 1982 [99]; Seljak, 2016, [116]).

⁠

**Specimens studied:**

⁠

**(1) Note. The *leucophaea*/*obliqua*/*eremi* issue.** Lindberg (1948) [38] characterized *Tettigometra obliqua* (Panzer, 1799), now *Erratometra leucophaea* (Preyssler, 1792), by a triangular mediodorsal aedeagal process (Figure 14A1), a subapical anal tube process, and variable dorsal coloration, by which it recognized several color forms. This taxon belongs to the genus *Erratometra* within the apexometrinan group, as confirmed here.

⁠

Lindberg (1948) [38] also introduced the name *eremi* Lindberg (1948) as a variety of *T. obliqua*, later treated directly at species rank by Emeljanov (1969: 368) [117], followed by Mitjaev (1971: 73) [64], Logvinenko (1975: 256) [66], and Mozaffarian et al. (2018: 481) [2]. He distinguished *eremi* by its very light yellowish coloration, nearly uniform dorsal surface, and an elongated vertex (0.8–1.0 mm), compared to western forms of *T. obliqua*. He also noted a difference in male genitalia: the dorsal tooth on the aedeagal process was more strongly developed in *eremi* (Figure 14A2) than in typical *T. obliqua*. However, Lindberg (*op. cit*.) did not indicate the locality of the figured specimen, nor did he illustrate the ventral anal tube process, implicitly assuming that it was subapical as in *T. obliqua*.

⁠

Our observations show that *obliqua* (now correctly *leucophaea*) can be well separated from *eremi sec.* Lindberg (1948) by the mediodorsal process shape: triangular and dorsally rounded in *leucophaea* versus stronger, more acute, and antero-dorsally directed in *eremi*. We therefore regard these two as distinct species as treated by Emeljanov (1969) [117].

⁠

By contrast, *eremi sensu* Mitjaev (1971: 73) [64] and *eremi sensu* Logvinenko (1975: Figures 224, 3 and 4) [66] correspond also to Lindberg’s written description (using in fact very polymorphic characters), but not to his illustration (Lindberg, 1948: Figure 27) [38], also refigured by Logvienko (1975: Figure 222.7) [66] as *obliqua*, not *eremi*. In both (*eremi sensu* Mitjaev, 1971 and *eremi sensu* Logvinenko, 1975), male genitalia show a basally constricted mediodorsal aedeagal process, high and apically rounded, combined with uniform pale coloration and an elongated vertex. According to Logvinenko (*op. cit.*), this is the same taxon concept already used by Emeljanov (1969) [117] when he first employed *eremi* as a species. Although the mediodorsal process not being fully finger-like-shaped, these genitalia match the *Mitricephalus* ***stat. nov.*** condition as redefined further in this paper. Accordingly, *Tettigometra obliqua sec.* Lindberg (1948) [38] represents a polyphyletic assemblage, and we distinguish at least three distinct taxon concepts, one of which remains unnamed.

⁠

From the nomenclatural point of view, Lindberg (1948) [38] originally introduced the name *Tettigometra obliqua var. eremi* as a variety of *T. obliqua*, without a formal diagnosis at species rank. According to the ICZN (Arts. 45.5.1–45.6.1), such infrasubspecific names are unavailable. The name *eremi* therefore remained unavailable until it was first adopted at the species level by Emeljanov (1969: 368) [117], who treated it as *Tettigometra eremi* Lindberg, 1948, thereby making the name available from that date, with authorship retained as Lindberg, 1948 (ICZN Art. 50.7).

⁠

From a taxonomic standpoint, three distinct concepts are now associated with this name:

(1) *Tettigometra obliqua* var*. eremi* Lindberg, 1948, as illustrated by Lindberg (1948: Figure 14A2) [38] and later by Logvinenko (1975: Figure 222.7) [66], corresponding to a valid species here recognized as *Erratometra eremi* (Lindberg, 1948) ***stat. nov.***, ***comb. nov.***
(2) *“Tettigometra eremi” sec.* Emeljanov (1969) [117], a misapplied name subsequently followed by Mitjaev (1971) [64] and Logvinenko (1975) [66], representing a different taxon referable to *Mitricephalus* ***stat. nov.***
(3) *Tettigometra obliqua sec.* Lindberg (1948) [38], corresponding to *Erratometra leucophaea* (Preyssler, 1792) ***comb. nov.***

⁠

To restore alignment between nomenclature and Lindberg’s original concept, *Tettigometra obliqua* var*. eremi* Lindberg, 1948 is here formally recognized at species rank and transferred to *Erratometra* as *E. eremi* (Lindberg, 1948) ***stat. nov.***, ***comb. nov.***, restricted to Lindberg’s original diagnosis and illustration. The taxon treated by Emeljanov [117] and subsequent authors remains unnamed pending formal description.

⁠

- ***Erratometra eremi*** **(Lindberg, 1948) *stat. nov.***

*Tettigometra obliqua* var*. eremi* Lindberg, 1948 (orig. comb.; infrasubsp. unavailable name).

*Tettigometra eremi* Lindberg, 1948 [available from Emeljanov, 1969: 368].

→*Erratometra eremi* (Lindberg, 1948) ***stat. nov.***, ***comb. nov.*** (present designation and transfer).

⁠

**Taxonomic status.** Valid species of *Erratometra*, here raised from varietal rank (***stat. nov.***) based on Lindberg’s (1948) [38] original concept and illustration. Distinct from *E. leucophaea* (Preyssler, 1792) ***comb. nov.*** and from “*T. eremi*” *sec.* Emeljanov (1969) [117], as illustrated by Logvinenko (1975: Figures 224, 3 and 4) [66] and Mitjaev (1971: 73) [64].

*E. eremi* differs from *E. leucophaea* by its mediodorsal aedeagal process, more acute and directed antero-dorsally (versus triangular and dorsally rounded in *E. leucophaea*). Lindberg (1948) [38] also noted a paler, almost uniformly yellow dorsal coloration and an elongated vertex (0.8–1.0 mm).

**Nomenclatural status.** Name available from Emeljanov (1969) [117] under ICZN Art. 50.7, with authorship retained as Lindberg, 1948 [38]. The concept used here follows Lindberg’s original variety and differs from Emeljanov’s later misapplied usage (see above).

⁠

**Illustrated.** Lindberg (1948: Figure 14A2) [38], based on type material for *Tettigometra obliqua* ssp. *eremi* as in Logvinenko (1975: Figure 222.7) [66], based on non-type material.

**Identified specimens.** Mozaffarian (HMIM)

**Dissected material.** Male genitalia examined in HMIM

⁠

- ***Erratometra leucophaea*** **(Preyssler, 1792) *comb. nov.***

*Cicada leucophaea* Preyssler, 1792 (original combination)

=*Fulgora obliqua* Panzer, 1799 (syn. by Dlabola, 1972: 207)

=*Tettigometra umbrosa* Germar, 1821 (syn. by Fieber, 1872a: 30)

→*Tettigometra obliqua* (Panzer, 1799) (transferred by Latreille, 1804: 312)

→*Tettigometra leucophaea* (Preyssler, 1792) (reinstated by Dlabola, 1972)

→*Erratometra leucophaea* (Preyssler, 1792) (present transfer)

⁠

**Taxonomic status.** Valid species of *Erratometra*. Type species of the genus *Erratometra* Mozaffarian & Bourgoin, ***gen. nov.***, here designated.

**Nomenclatural status.** Name available and valid (*Cicada leucophaea* Preyssler, 1792). Under ICZN Art. 23.9 (“Reversal of precedence”), *obliqua* Panzer, 1799, although long used, cannot be maintained as valid because *leucophaea* has been in prevailing usage in several works since 2000. Therefore, *leucophaea* is treated as the valid name, and *obliqua* as its junior synonym as reported by Dlabola (1972) [118].

⁠

**Illustrated.** Lindberg (1948) [38]; Mitjaev (1971) [64]; Anufriev & Emeljanov (1988) [68], all based on non-type material.

**Identified specimens.** As *Tettigometra obliqua* Panzer, 1799 by Bergevin, Dlabola, Noualhier–Lethierry, Oshanin, Perrier, Puton, and Ribaut (MNHN coll.).

**Dissected material.** Male genitalia examined from the Dlabola collection (MNHN).

⁠

**Note.** The synonymy of *obliqua* under *leucophaea* was first proposed by Dlabola (1972) [118]. Nast (1987) [119] attempted to preserve *obliqua* as a nomen protectum with *leucophaea* a nomem oblitum. However, this would have required a formal application under the pre-2000 ICZN, which was never submitted. Since the 4th edition of the Code, the conditions of Art. 23.9.1 are not met in favor of *obliqua*, given the documented prevailing use of *leucophaea*.

⁠

**(2) Other *Erratometra* specimens studied:**

- ***Erratometra bifoveolata*** **(Signoret, 1866) *comb. nov.***

*Tettigometra (Eurychila) bifoveolata* Signoret, 1866 (original combination)

→*Erratometra bifoveolata* (Signoret, 1866) ***comb. nov.***(present transfer)

⁠

**Taxonomic status.** Valid species of *Erratometra* ***gen. nov.***

**Nomenclatural status.** Name available and valid (Signoret, 1866) [35].

⁠

Illustrated. No original type illustration; subsequent figures provided by Lindberg (1948) [38] (non-type material, without male genitalia).

⁠

Identified specimens. Dlabola, Noualhier–Lethierry, Perrier, Puton, and Ribaut (MNHN)

Dissected material. Male genitalia examined from sthe Dlabola collection (MNHN).

- ***Erratometra lyncea*** **(Horváth, 1903) *comb. nov.***

*Tettigometra lyncea* Horváth, 1903 (original combination)

→*Erratometra lyncea* (Horváth, 1903) ***comb. nov.*** (present transfer)

⁠

**Taxonomic status.** Valid species of *Erratometra* ***gen. nov.***

**Nomenclatural status.** Name available and valid (Horváth, 1903) [120].

⁠

**Identified specimens.** Ribaut (MNHN).

**Dissected material.** Male genitalia examined from the Ribaut collection (MNHN).

⁠

**Note.** The species was originally described from Serbia, but has rarely been cited in later literature. Male genitalia material examined here confirms placement in the apexometrinan group and justifies transfer to *Erratometra* ***gen. nov.***

⁠

- ***Erratometra sordida*** **(Fieber, 1865) *comb. nov.***

Figure 7j

*Tettigometra sordida* Fieber, 1865 (original combination)

→*Erratometra sordida* (Fieber, 1865) ***comb. nov.*** (present transfer)

⁠

**Taxonomic status.** Valid species of *Erratometra* ***gen. nov.***, into which genus it is transferred. Although Holzinger (2009) placed *T. sordida* in synonymy with *T. griseola* Fieber, 1865, this synonymy seems unsupported and published without reference to evidence. Based on our dissected material, we maintain *sordida* as a distinct valid species from *Tettigometra griseola* by the shape of the mediodorsal aedeagal process, which in *sordida* is more robust, triangular, and apically rounded, whereas in *griseola* it is more slender, elongate, and subrectangular (cf. Lindberg, 1948 [38]; Logvinenko, 1975 [66]). The latter species was here transferred to the genus *Apexometra*. ***gen. nov.***

**Nomenclatural status.** Name available and valid (Fieber, 1865) [34].

⁠

**Illustrated.** Lindberg (1948) [38]; Logvinenko (1975) [66], both based on non-type material.

**Identified specimens.** Dlabola (MNHN collections).

**Dissected material.** Male genitalia examined from the Dlabola collection (MNHN).

⁠

- ***Erratometra stepposa* (Logvinenko, 1975) *comb. nov.***

*Tettigometra stepposa* Logvinenko, 1975 (original combination)

→*Erratometra stepposa* (Logvinenko, 1975) ***comb. nov.*** (present transfer)

⁠

**Taxonomic status.** Valid taxon species of *Erratometra* ***gen. nov.***

**Nomenclatural status.** Name available and valid (Logvinenko, 1975) [66]

⁠

**Illustrated.** Logvinenko (1975: Figure 225) [66], based on original type material.

**Identified specimens.** Not found in MNHN collections.

⁠

**Note.** The species was originally described from steppe localities in Ukraine. Examination of original description and figures (Logvinenko, 1975: 259, Figure 227) [66] confirms placement within the apexometrinan group, with a mediodorsal aedeagal process robust, triangular, and apically rounded, consistent with the generic diagnosis of *Erratometra* ***gen. nov.***

⁠

**(3) Misidentified specimens:**

- **Unidentified (new?) species**

*Tettigometra pantherina* Horváth, 1891 *sec.* Lindberg, 1948 *nec* Horváth 1891, *nec* auth.

⁠

**Illustrated.** Lindberg (1948: Figure 20C, based on non-type material) [38].

⁠

**Note.** This species is clearly distinct from *Tettigometra pantherina* as originally described (Horváth, 1891) [75], which belongs now to the genus *Persiametra* ***gen. nov.*** In Lindberg’s (1948: Figure 20C) [38], the male genitalia show a strongly developed mediodorsal aedeagal process, triangular to trapezoidal and apically rounded, together with a subapical anal tube process, both character states consistent with the genus *Erratometra* ***gen. nov.*** in the apexometrinan group. By contrast, *Persiametra pantherina* is characterized by absence of the mediodorsal aedeagal process and paired submedian ventral anal processes, the defining features of *Persiametra* ***gen. nov.*** This discrepancy strongly suggests that Lindberg inadvertently illustrated another species under Horváth’s name. Pending re-examination of Lindberg’s original material, we treat this concept as an unidentified species of *Erratometra* ***gen. nov.***, not cogeneric with *P. pantherina*.

⁠

- **Unidentified (misidentified?) species**

*Tettigometra cerina* Lindberg, 1948, *sec.* Mitjaev 1971 Figures 17. 14–17)

⁠

**Illustrated.** Mitjaev 1971 Figures 17. 14–17), based on non-type material.

⁠

**Note.** The specimens illustrated by Mitjaev (1971) [64] lack the characteristic digitiform mediodorsal aedeagal process diagnostic of *Mitricephalus*, the genus to which the nominal species is attributed in the present work. In Lindberg’s original description, this process is strongly developed, finger-like, and parallel-sided in lateral view. By contrast, Mitjaev figured a broad triangular mediodorsal process, apically rounded, fully consistent with the *Erratometra* generic concept as defined here. These specimens therefore do not correspond to *M. cerina* Lindberg, 1948, but represent material referable to *Erratometra* and are likely misidentified.

⁠

**3. Genus *Eurychila* Signoret 1866, *stat. rev.***

⁠

**Type species:** *Eurychila decorata* (Signoret, 1866) by subsequent designation by Baker, 1924: 93.

⁠

**Taxonomic status.** *Eurychila* Signoret, 1866 is here reinstated as a valid genus of the apexometrinan group within Tettigometrini, upgraded from its former subgeneric rank as treated by Emeljanov (1980) [29].

**Nomenclatural status.** Name available and valid (Signoret, 1866) [35]. Type species fixed by subsequent designation of Baker (1924) [36]. Gender: Feminine.

⁠

**Diagnosis.** Anal tube with paired ventral processes in subapical position (Figure 3). Together with *Persiametra* ***gen. nov.*** and *Stirometra **stat. nov.***, *Eurychila* ***stat. rev.*** differs from all other tettigometrine genera by lacking a distinct dorsal aedeagal process. It separates from *Persiametra* ***gen. nov.*** by having subapical (not submedian) ventral anal processes, and from *Stirometra **stat. nov.*** by the proportions of the anal tube and aedeagus: in *Eurychila* ***stat. rev.***, the anal tube is longer than high in lateral view and bears a regular pair of ventral processes, whereas in *Stirometra* ***stat. nov.***, it is shorter than high, and the ventral processes are more strongly developed. In addition, the aedeagus in *Eurychila* ***stat. rev.*** is distinctly weakly swollen dorsally in place of the mediodorsal aedeagal process (Figure 6g), versus straight, totally absent in *Stirometra* ***stat. nov.*** (Figure 3).

⁠

**Included species.** *Eurychila afra* (Kirschbaum, 1868) n. stat., ***comb. nov.***, *Eurychila decorata* (Signoret, 1866) ***comb. rev.***; *Eurychila fasciata* (Rambur, 1865) ***comb. nov.***; *Eurychila tafratensis* (Bergevin, 1920) ***comb. nov.***

**Geographical distribution:** Northern Africa [Algeria, Morocco, Tunisia], Southern Europe [Spain], Western Asia [Israel, Libya, Palestine, Syria] (Lindberg, 1963; Nast, 1972; Bourgoin, 1985, 2025).

⁠

**Specimens studied:**

- ***Eurychila afra*** **(Kirschbaum, 1868) *stat. nov., comb. nov.***

(Figure 7h)

*Tettigometra afra* Kirschbaum, 1868: 58 [104]. (original combination)

≠*Tettigometra brunnea* Signoret, 1866 (err. syn. by Horváth (1912), rejected herein.)

≠*Tettigometra picea* Kirschbaum, 1865 (err. syn. by Horváth (1912), rejected herein.)

→*Eurychila afra* (Kirschbaum, 1868) ***stat. nov.*, *comb. nov.***

⁠

**Taxonomic status.** Valid species of *Eurychila* Signoret 1866, ***stat. rev.***

**Nomenclatural status.** Name available and valid (Kirschbaum, 1868) [104].

⁠

**Illustrated.** No known illustrations of type material.

**Identified and dissected specimens.** Puton, and Noualhier–Lethierry (MNHN), identified by both authors as *T. afra* Kirschbaum.

**Dissected material.** Male genitalia examined from the Noualhier–Lethierry collection (MNHN).

⁠

**Additional diagnosis.** Body larger and broader than *T. picea*; coloration darker and more uniform; pronotum moderately convex; tegmina longer and more rounded apically, with a distinct costal groove. Frons without basal pale band, slightly broader and less convex in lateral view. Aedeagus lacking distinct dorsal process typical, only inflated here (Figure 6g).

⁠

**Note.** The synonymy proposed by Horváth (1912) [106] between *T. afra* and *T. brunnea* was based solely on superficial external morphology and is rejected here. Specimens identified as *afra* from the Puton, and Noualhier–Lethierry collections correspond to the same species concept and differ markedly from *brunnea sec.* Puton coll specimen which probably represent the original concept of *T. brunnea* Signoret, 1866.

⁠

- ***Eurychila decorata* (Signoret, 1866) *comb. rev.***

*Tettigometra* (*Eurychila*) *decorata* Signoret, 1866: 156 (original combination)

→*Tettigometra decorata* Signoret, 1866 (Fieber, 1872)

→*Eurychila decorata* (Signoret, 1866) (Metcalf, 1932)

→*Tettigometra* (*Eurychila*) *decorata* Signoret, 1866 (Emeljanov, 1980)

→*Eurychila decorata* (Signoret, 1866) ***comb. rev.***

⁠

**Taxonomic status.** Valid species of *Eurychila*.

**Nomenclatural status.** Name available and valid (Signoret, 1866) [35]. Type species of the genus *Eurychila* Signoret, 1866 by subsequent designation.

⁠

**Illustrated.** Lindberg (1948); Bourgoin (1988b), both based on non-type material.

**Identified specimens.** Bergevin, Puton, Noualhier-Lethierry (MNHN).

**Dissected material.** Male genitalia examined from Noualhier-Lethierry (MNHN).

⁠

**Note.** This species was originally described from Algeria by Signoret (1866). It is characterized by a distinct dorsal swelling of the aedeagal shaft and by metatibiae with 7 apical teeth, a feature consistent with Emeljanov’s (1980) key to genera for *Eurychila*. In contrast, specimens in the MNHN collection identified as *E. decorata* by Dlabola bear 9 metatibial apical teeth and lack the mediodorsal swelling. These specimens likely represent a probably undescribed species within the genus *Stirometra* of the apexometrinan group.

⁠

- ***Eurychila fasciata*** **(Rambur, 1840) *stat. nov., comb. nov.***

*Mijas fasciata* Rambur, 1840: 209 (original combination)

→*Tettigometra fasciata* (Rambur, 1840) (tranferred by Webb, 1979: 236)

→*Eurychila fasciata* (Rambur, 1840) ***stat. nov.*, *comb. nov.***

⁠

**Taxonomic status.** Valid species (Webb, 1979) [121], transferred in *Eurychila* Signoret 1866, ***stat. rev.***

**Nomenclatural status.** Originally described as *Mijas fasciata* Rambur, 1840 [122]; transferred to *Tettigometra* by Webb (1979), who redescribed the species from the type. Following *Nast* (1984: 394 [123]), the name *Tettigometra fasciata* Fieber, 1865 does not represent a distinct taxon but rather a subsequent usage of *Mijas fasciata* Rambur, 1840 as determined by Meyer-Dür, whose material Fieber had examined. Consequently, no homonymy exists between Rambur’s and Fieber’s names, and *Eurychila fasciata* (Rambur, 1840) is retained as the valid name.

⁠

**Illustrated.** Webb, 1979 (based on the male holotype in BMNH; figs 48).

**Identified specimens.** by Dlabola (however not fully comparable with Webb description), Noualhier–Lethierry, Puton (MNHN).

⁠

**Note.** According to Webb (1979) [121], the male pygophore and 10th segment are typical of the genus [*Tettigometra*] (sic), while the aedeagus is distinctive in having a swollen basal region without a dorsal tooth. Webb also suggested that *T. fasciata* Rambur might prove to be a senior synonym of *T. fasciata* Fieber, 1865, a conclusion confirmed later by Nast (1984: 394) [123], who demonstrated that Fieber’s name was not newly described (homonym) but a mere adoption of Rambur’s species via Meyer-Dür’s material. Material from the Noualhier–Lethierry and Puton collections (MNHN) identified as *T. fasciata* Fieber, 1865 agrees with Webb (1979) [121]. We therefore follow here Webb (1979) [121] and Nast (1984) [123] and in recognizing *Eurychila fasciata* (Rambur, 1840) as a valid species of *Eurychila* ***stat. rev.***

⁠

- ***Eurychila tafratensis*** Bergevin, 1920, ***comb. rev.***

*Tettigometra tafratensis* Bergevin, 1920 (original combination)

→*Eurychila tafratensis* (Bergevin, 1920) (Nast, 1972)

→*Tettigometra* (*Mitricephalus*) *tafratensis* Bergevin, 1920

→*Eurychila tafratensis* (Bergevin, 1920) ***comb. rev.***

⁠

**Taxonomic status.** Valid species transferred in *Eurychila* Signoret 1866, ***stat. rev.*** Nomenclatural status. Name available and valid (Bergevin, 1920) [124].

⁠

**Illustrated.** Lindberg (1948, based on non-type material).

**Identified specimens.** Bergevin (two ♀♀ in MNHN, one of which is a type).

**Dissected material.** No male specimens examined; female type material checked in MNHN.

⁠

**Note.** The species remains poorly known. Bergevin (1920) [124] described it originally from female material only from Morocco. According to Lindberg (1948) [38], *T. tafratensis* is remarkable within *Tettigometra s.l.*: the frons is irregularly rugose in its upper part, with a distinct median depression below the apex; the eyes are less broad than in related species; and the forewings are posteriorly widened. The aedeagus is distinctive, with a narrow median part slightly elevated, concave on the sides.

⁠

**4. Genus *Hystrigonia* Emeljanov, 1980, *stat. nov.***

(Figure 7a)

**Type species:** *Hystrigonia hexaspina* (Kolenati, 1857), by monotypy, Emeljanov, 1980: 55

⁠

**Taxonomic status.** *Hystrigonia* Emeljanov, 1980 is retained here as a valid monotypic genus of the apexometrinan group within Tettigometrini.

**Nomenclatural status.** Name available and valid (Emeljanov, 1980). Type species fixed by monotypy (Emeljanov, 1980: 55). Gender: Feminine.

⁠

**Amended diagnosis.** In addition to Emeljanov’s (1980) diagnosis [29], which established the subgenus *Hystrigonia* based solely on the presence of specific body setae—translucent with brittle apices (see his key, 1980: 54)—the anal tube is short: ventral margin less than twice as long as the dorsal one, versus about twice in *Micracanthometra* ***gen. nov.*** and *Apexometra **gen. nov.*** As in the former, the mediodorsal aedeagal process is short and triangular (i.e., pointed) (Figure 5d), in contrast to Emeljanov’s original description of it as a “conical process”.

⁠

**Note.** *Hystrigonia* is a very narrowly defined monotypic genus established by Emeljanov (1980) [29], whose current diagnosis likely captures only species-specific traits. As such, it may exclude closely related species now placed in *Micracanthometra* ***gen. nov.***, particularly or perhaps even *Apexometra **gen. nov.*** Indeed, a similar short triangular mediodorsal aedeagal process is present in *Micracanthometra impressifrons* and *M. picta*, but in these species, the anal tube is longer and its ventral margin is approximately twice the length of the dorsal margin. In other *Apexometra* ***gen. nov.*** species, the mediodorsal process is more developed and exhibits a distinctly transverse dorsal margin. Despite these potential affinities, *Hystrigonia* is retained here as a valid monotypic genus, pending a clearer understanding of the phylogenetic relationships within Tettigometrini, particularly by incorporating molecular sequence data in future studies.

⁠

**Included species:** *Hystrigonia hexaspina* (Kolenati, 1857)

⁠

**Geographical distribution:** Southern Europe [Italy, Greece, Portugal, Serbia, Spain], Eastern Europe [Bulgaria, Ukraine], Western Asia [Armenia, Dagestan, Georgia, Israel, Jordan, Turkey], Southern Asia [Iran] (Mozaffarian et al., 2018 [1]; Bourgoin, 2025 [2]; Gradojević, 1931 [9]; Nast, 1972 [52]; Demir, 2006 [65]; Logvinenko, 195 [66]; Mozaffarian & Wilson, 2011 [69]; Lindberg, 1949 [78]; Dlabola, 1965a [81]; Jankovic, 1966b [114]).

⁠

**Specimens studied:**

- ***Hystrigonia hexaspina*** **(Kolenati, 1857)**

(Figure 7a)

*Tettigometra hexaspina* Kolenati, 1857 (original combination)

=*Tettigometra hispidula* Fieber, 1865 (syn. by Oshanin, 1907: 282)

=*Tettigometra callosa* Signoret, 1866 (syn. by Fieber, 1872b: 2)

→*Hystrigonia hexaspina* (Kolenati, 1857) (transferred by Emeljanov, 1980: 55)

**Taxonomic status.** Valid species of *Hystrigonia*, the sole species of the genus.

**Nomenclatural status.** Name available and valid (Kolenati, 1857) [125]. Transferred to *Hystrigonia* by Emeljanov (1980) [29].

⁠

**Illustrated.** Lindberg (1948) [38], Logvinenko (1975) [66], Emeljanov (1980) [29] (all based on non-type material).

**Identified specimens.** As *T. hispidula* Fieber, 1865 by Bergevin, Noualhier–Lethierry, Puton (MNHN collections), and as *T. hexaspina* by Mozaffarian (HMIM).

**Dissected material.** Male genitalia dissected in HMIM (Figure 6d).

⁠

**5. Genus** ***Micracanthometra*** **Mozaffarian & Bourgoin, *gen. nov.***

urn:lsid:zoobank.org:act:36BF15E7-0BF3-424A-8273-7DD059DF8417

⁠

**Type species.** *Tettigometra impressifrons* Mulsant & Rey, 1855, here designated.

⁠

**Etymology.** From Greek *mikros* (small) and *akantha* (spine, thorn), in reference to the strongly reduced triangular mediodorsal process of the aedeagus, combined with the generic suffix *-metra* (as in *Tettigometra*). Feminine gender.

⁠

**Taxonomic status.** *Micracanthometra* ***gen. nov.*** is here established as a valid genus of the apexometrinan group within Tettigometrini, to accommodate species with a reduced triangular mediodorsal aedeagal process.

**Nomenclatural status.** Name newly proposed and available under ICZN Art. 13.1, with type species fixed by present designation.

⁠

**Diagnosis.** *Micracanthometra* ***gen. nov.*** is distinguished within the apexometrinan group by the following combination of characters: anal tube longer than high (Figure 6b), with a straight dorsal margin and the ventral anal process positioned subapically; mediodorsal aedeagal process strongly reduced, represented only by a minute triangular projection; tegument relatively smooth and shiny in *M. impressifrons* (Mulsant & Rey, 1855), ***comb. nov.*** and *M. contracta* (Lindberg, 1948) ***comb. nov.***, and densely punctate. Brachypterous forms occur. It differs from *Stirometra* ***stat. nov.*** (anal tube shorter than high) and *Eurychila* ***stat. rev.*** by the presence of a distinct mediodorsal process; from *Hystrigonia* ***stat. nov.*** by the straight dorsal margin of the anal tube (versus strongly concave in the latter) and absence of the bristle-like setae; and from *Erratometra* ***gen. nov.*** and *Apexometra* ***gen. nov.*** by the minute mediodorsal process (versus, respectively, stronger triangular conformations and a subquadrangular, double-pointed process) (Figure 3).

⁠

**Included species.** *Micracanthometra impressifrons* (Mulsant & Rey, 1855) ***comb. nov.***; *Micracanthometra picta* (Fieber, 1865) ***comb. nov.***; *Micracanthometra contracta* (Lindberg, 1948) ***comb. nov.***

⁠

**Geographical distribution:** Northern Africa [Algeria, Libya, Morocco, Tunisia], Southern Europe [Italy, Malta, Spain], Western Europe [Belgium, France], Eastern Europe [Greece], Western Asia [Israel, Jordan, Syria], Southern Asia [Iran] (Mozaffarian et al., 2018 [1]; Bourgoin, 2025 [2]; Fokker, 1900 [48]; Lindberg, 1948 [38]; Nast, 1972 [52]; Albre & Gibernau, 2019 [56]; D’Urso et al., 2019 [57]; Mozaffarian & Wilson, 2011 [69]; Lindberg, 1963 [79]; Dlabola, 1965a [81]; Linnavuori, 1965 [95]).

⁠

**Specimens studied:**

- ***Micracanthometra contracta* (Lindberg, 1948), *comb. nov.***

*Tettigometra contracta* Lindberg, 1948 (original combination)

→*Micracanthometra contracta* (Lindberg, 1948) ***comb. nov.*** (present transfer)

⁠

**Taxonomic status.** Species of *Micracanthometra* (transferred from *Tettigometra*).

**Nomenclatural status.** Name available and valid (Lindberg, 1948) [38].

⁠

**Illustrated.** Lindberg (1948) [38], based on type material.

**Identified specimens.** No specimen was available for examination in the present study.

⁠

- ***Micracanthometra impressifrons* (Mulsant & Rey, 1855), *comb. nov.***

*Tettigometra impressifrons* Mulsant & Rey, 1855 (original combination)

→*Tettigometra (Tettigometra) impressifrons* Mulsant & Rey, 1855 (Emeljanov, 1980: 54)

→*Micracanthometra impressifrons* (Mulsant & Rey, 1855), ***comb. nov.***(present transfer)

**Taxonomic status.** Type species of *Micracanthometra* ***gen. nov.*** (transferred from *Tettigometra* (*Tettigometra*).

**Nomenclatural status.** Name available and valid (Mulsant & Rey, 1855) [126].

⁠

**Illustrated.** Lindberg (1948) [38], based on non-type material.

**Identified specimens.** Bergevin, Dlabola, Noualhier–Lethierry, Perrier, Puton, Ribaut (MNHN), and Mozaffarian (HMIM).

**Dissected material.** Male genitalia examined in HMIM.

⁠

- ***Micracanthometra picta* (Fieber, 1865), *comb. nov.***

(Figure 7b)

*Tettigometra picta* Fieber, 1865 (original combination)

=*T. marginepunctata* Kirschbaum, 1868 (syn. by Fieber (1872a: 30).

→*Tettigometra (Tettigometra) picta* Mulsant & Rey, 1855 (Emeljanov, 1980: 54)

→*Micracanthometra picta* (Fieber, 1865), ***comb. nov.*** (present transfer)

**Taxonomic status.** Species of *Micracanthometra **gen. nov.***, transferred from *Tettigometra* (*Tettigometra*).

**Nomenclatural status.** Name available and valid (Fieber, 1865) [34]. Synonymized with *T. marginepunctata* Kirschbaum, 1868 by Fieber (1872a) [59].

⁠

**Illustrated.** Lindberg (1948) [38]; Logvinenko (1975) [66], both based on non-type material.

**Identified specimens.** Bergevin, Dlabola, Oshanin, Perrier, Puton, Ribaut (MNHN collections).

**Dissected material.** Male genitalia examined from the Dlabola collection (MNHN).

⁠

**Note. The *Mitricephalus*/*Macrometrina* issue.** The taxonomic distinction between *Mitricephalus* Signoret, 1866 and *Macrometrina* Lindberg, 1948 rests on weakly supported characters. Below, we summarize the historical treatments, the morphological bases originally proposed for their separation, and our rationale for retaining them provisionally as distinct genera.

⁠

Following Fieber (1872b) [60], Metcalf (1932) [37] treated *Mitricephalus longiceps* Signoret, 1866 as a junior synonym of *Tettigometra macrocephala* Fieber, 1865, thereby establishing *macrocephala* as the valid type species of *Mitricephalus* Signoret, 1866 (by subsequent designation of Baker, 1924) [36]. Lindberg (1948) [38], followed Fieber’s synonymy and left alone *T. macrocephala* in the subgenus *Mitricephalus*. He also erected a new genus, *Macrometrina*, also for a single species: *T. grossa*. Emeljanov (1980) [29], in his key, accepted Lindberg’s division and maintained both genera as distinct, but without offering any new justification beyond Lindberg’s original proposal.

⁠

According to Lindberg (1948) [38] and Emeljanov (1980) [29], Mitricephalus is characterized by a narrow ocular callus, the portion outside the eye margin being less than one-third of the eye’s width in dorsal view. The scutellum is distinctly shorter than the combined length of the vertex and pronotum, and the frons in profile is concave. In addition, the vertex is longer than the pronotum, the body is rather flattened, and the tegmina are only weakly elevated posteriorly in lateral view. In contrast, Macrometrina is distinguished by a broader ocular callus, equal to or exceeding one-third of the eye’s width in dorsal view. The scutellum is markedly longer than the vertex and pronotum together, and the frons is described as strongly concave in profile. Externally, the vertex remains longer than the pronotum, but the body is only moderately flattened and the tegmina are conspicuously more elevated posteriorly in lateral view.

⁠

In other words, the most reliable character states proposed by Lindberg’s (1948) key to separate *Mitricephalus* from *Macrometrina* are the relative proportions of the scutellum, much shorter than vertex plus pronotum in *Mitricephalus*, distinctly longer in *Macrometrina*, and the condition of the tegmina, weakly elevated posteriorly in *Mitricephalus* versus strongly elevated in *Macrometrina* (as emphasized by Emeljanov, 1980 [29]). These traits would appear to set both taxa apart from all other genera of the tribe. However, our observations indicate that the first character does not hold consistently, since in all Tettigometrini the scutellum is shorter than the combined length of vertex and pronotum. However, the alternative condition is observed in other Tettigometridae taxa, particularly members of African Nototettigometrinae. Moreover, the male genitalia are unequivocally of the same type in both genera, characterized by paired ventral anal processes in subapical position and a strongly developed, digitiform mediodorsal process, apically rounded, with parallel or slightly divergent margins.

⁠

Since the two genera erected by Lindberg were both monotypic, it is most likely that the character states he defined reflect species-level differences only, rather than supporting a generic separation. Based on the new generic framework developed here from male genitalia characters for the *Tettigometra* concept sensu lato, we therefore consider that the two taxa might represent the same generic concept, with *Macrometrina* Lindberg, 1948 as a junior synonym of *Mitricephalus* Signoret, 1866. However, as noted earlier, we deliberately refrain from establishing any formal synonymy in this paper pending further molecular analyses. For practical and operational reasons, we prefer to retain them as separate entities for now. Maintaining both names as valid facilitates consistent handling of existing identifications, historical literature, and associated biological data. Prematurely merging them would risk obscuring these records and create additional instability, particularly if future analyses were to support their separation again. Accordingly, we maintain the two genera as valid and keep them artificially separated in the key and in the present revision.

⁠

**6.** **Macrometrina** **Lindberg, 1948** **stat. nov.**

⁠

**Type species.** *Tettigometra grossa* Lindberg, 1948, by original designation and monotypy (Lindberg, 1948) [=*Tettigometra fusca* Melichar, 1902].

⁠

**Taxonomic status.** *Macrometrina* Lindberg, 1948 is provisionally retained here as a valid genus of the apexometrinan group within Tettigometrini. However, based on the male genitalia, it is likely congeneric with *Mitricephalus* Signoret, 1866 ***stat. rev.***, and may represent only a junior synonym of it. For practical reasons and pending molecular evidence, we maintain both as separate genera in this revision.

**Nomenclatural status.** Name available and valid (Lindberg, 1948) [38]. Type species fixation by original designation. Gender: Feminine.

⁠

**Diagnosis.** *Macrometrina* shares with *Mitricephalus* ***stat. rev.*** the distinctive configuration of the male genitalia, with paired ventral anal processes in subapical position and a strongly developed digitiform mediodorsal aedeagal process, apically rounded, with parallel or slightly divergent margins (Figure 3). The condition contrasts with the triangular, quadrangular, or otherwise modified processes found in other apexometrinan genera, and with the angulate, submedian configuration in *Mediodentometra* ***gen. nov.*** in the tettigometrinan group. Externally, however, *Macrometrina* differs from *Mitricephalus* in having tegmina shaped diffrently (Emeljanov, 1980, Figure 1. 6–7) [29] and more conspicuously elevated posteriorly in lateral view, giving the body a higher posterior profile.

⁠

**Note.** As indicated above, the tegmina character states attributed to *Macrometrina* are most likely of specific rather than generic value, and this monospecific genus might represent a junior synonym of *Mitricephalus*, both sharing the same generic concept. Molecular analyses will be necessary to formally accept this probable synonymy.

⁠

Included species: *Macrometrina fusca* (Melichar, 1902) ***comb. nov.***

⁠

**Geographical distribution:** Central Asia [Kazakhstan], Northern Asia [Siberia], Eastern Asia [China, Mongolia, Russian Far East, South Korea], Southeastern Asia [Maritime Territory] (Lindberg, 1948 [38]; Dlabola, 1965b [96], 1970 [97]; Anufriev, 1969 [98]; Nast, 1972 [52]; Kwon & Lee, 1978 [127]; Anufriev & Emeljanov, 1988 [68]; Bourgoin, 2025 [2]).

⁠

**Specimens studied:**

- ***Macrometrina fusca*** **(Melichar, 1902)**

⁠

Figure 7g

*Isthmia fusca Milichar, 1902*

→*Hilda fusca* (Melichar, 1902) (***comb. nov.*** by Oshanin, 1907: 275)

=*Macrometrina grossa* (Lindberg, 1948) (syn. by Anufriev, 1969: 179)

=*Tettigometra (Macrometrina*) *grossa* Lindberg, 1948; invalid replacement name owing to secondary homonymy by Emeljanov, 1980: 55)

=*Tettigometra koreana* Kwon & Lee, 1978 (syn. by Anufriev & Emeljanov, 1988: 320

→*Macrometrina fusca* (Melichar, 1902) (present combination)

⁠

**Taxonomic status.** Valid species of *Macrometrina* Lindberg, 1948 ***stat. nov.*** encompassing material previously referred to *Tettigometra grossa* Lindberg, 1948 and *T. koreana* Kwon & Lee, 1978.

**Nomenclatural status.** Name available and valid (Melichar, 1902). Under ICZN Article 23 (Principle of Priority), *fusca* Melichar, 1902 has precedence over *grossa* Lindberg, 1948. The synonymy proposed by Anufriev (1969) [98]. Emeljanov (1980) considered *grossa* as the valid name because of homonymy which was not in agreement with the Code. The homonymy of *fusca* Melichar 1902 with *fusca* Fieber, 1865 is only secondary (Art. 59.2), and does not invalidate *fusca* Melichar, which remains the correct and valid name. Anufriev & Emeljanov (1988) [68] subsequently treated *koreana* Kwon et Lee, 1978 as a junior synonym of *fusca* (Melichar, 1902), effectively restoring the correct usage of *fusca*.

⁠

**Illustrated.** As *Tettigometra grossa* Lindberg, 1948 by Lindberg (1948) [38], based on type material; as *Tettigometra fusca* (Melichar, 1902) by Emeljanov (1980) [29], based on non-type material; as *Tettigometra koreana* Kwon & Lee, 1978 by Kwon & Lee (1978) [127], based on type material.

**Identified specimens.** Dlabola, Noualhier–Lethierry (MNHN collections).

⁠

**Note.** Lindberg (1948) [38] described *T. grossa* as a large, strongly built species (5.2–5.8 mm), with tegmina conspicuously elevated posteriorly and frons strongly concave in profile, emphasizing the long, tongue-shaped mediodorsal aedeagal process. Anufriev (1969) [98] treated *grossa* as conspecific with *T. fusca* Melichar, 1902; then, Emeljanov (1980) regarded *grossa* as the valid name because of homonymy. Later, Anufriev & Emeljanov (1988) [68] synonymized *T. koreana* Kwon & Lee, 1978 with *T. fusca* (Melichar, 1902) but without comment, indirectly reinstating *fusca* as the valid name. We follow this treatment here, recognizing *Macrometrina fusca* (Melichar, 1902) as the valid name, with *grossa* and *koreana* as junior synonyms.

⁠

**7. Genus** ***Mitricephalus*** **Signoret, 1866 *stat. rev.***

⁠

**Type species.** *Tettigometra macrocephala* Fieber, 1865, by subsequent designation of Baker, 1924 (as clarified by Metcalf, 1932).

⁠

**Taxonomic status.** *Mitricephalus* Signoret, 1866 is retained here as a valid genus of the apexometrinan group within Tettigometrini. *Macrometrina* Lindberg, 1948 is likely a junior synonym of it, but synonymy is not formally established here.

Nomenclatural status. Name available and valid (Signoret, 1866) [35]. Type species fixation by subsequent designation of Baker (1924) [36], who referred to *Tettigometra macrocephala* Fieber, 1865 (following Fieber, 1872b [60], who synonymized *M. longiceps* with *macrocephalus*). Gender: Masculine.

⁠

**Diagnosis.** Members of *Mitricephalus* ***stat. rev.*** are characterized by a strongly developed, digitiform mediodorsal aedeagal process (Figure 6e), apically rounded, with parallel or slightly divergent margins. This condition contrasts with the triangular to trapezoidal, apically rounded process of *Erratometra **gen. nov.***, the quadrangular process of *Apexometra **gen. nov.***, and the other distinct shapes observed in remaining apexometrinan genera (Figure 3). In the tettigometrinan group, a superficially similar mediodorsal process occurs in *Mediodentometra **gen. nov.***, but *Mitricephalus* ***stat. rev.*** is readily separated by the subapical position of the paired ventral anal processes (submedian in *Mediodentometra **gen. nov.***, where the mediodorsal process is also angulate rather than rounded, Figure 3). Externally, *Mitricephalus* ***stat. rev.*** species are distinguished from *Macrometrina* ***stat. nov.*** by tegmina shape and conformation only weakly elevated posteriorly in lateral view, the body appearing more flattened (Emeljanov, 1980, Figure 1. 6–7) [29].

⁠

**Included species.** *Mitricephalus cerina* (Lindberg, 1948) ***comb. nov.***; *Mitricephalus macrocephalus* (Fieber, 1865); *Mitricephalus varia* (Fieber, 1865) and two unidentified species: *Mitricephalus sordida* Fieber, 1865 *sec.* Mitjaev, 1970 and *Mitricephalus eremi* Lindberg, 1948 *sec.* Mitjaev, 1971 *sec.* Logvinenko, 1975.

⁠

**Geographical distribution:** Northern Africa [Algeria], Southern Europe [Macedonia, Serbia, Slovenia], Western Europe [Austria, Belgium, France, Germany, Switzerland], Eastern Europe [Bulgaria, Czech Republic, Romania, Slovakia, Ukraine], Western Asia [Armenia, Azerbaijan, Jordan Georgia, Turkey], Southern Asia [Afghanistan, Iran], Central Asia [Kazakhstan, Kirghizia, Tadzhikistan, Uzbekistan], Northern Asia [Siberia] (Mozaffarian et al., 2018 [1]; Bourgoin, 2025 [2]; Signoret, 1866 [35]; Lindberg, 1948 [38]; Fokker, 1900 [48]; Jankovic, 1966 [49], 1971a [50]; Nast, 1972 [52]; Logvinenko, 1975 [66]; Dlabola, 1957, [67]; Mozaffarian & Wilson, 2011 [69]; Dlabola 1965a [81]; Holzinger & Seljak, 2001 [84]; Jankovic,1978 [128]).

⁠

**Specimens studied:**

- ***Mitricephalus cerina*** **(Lindberg, 1948) *comb. nov.***

*Tettigometra cerina* Lindberg, 1948 (original combination)

→*Mitricephalus cerina* (Lindberg, 1948) ***comb. nov.*** (present work)

⁠

**Taxonomic status.** Valid species of *Mitricephalus* ***stat. rev.***, here transferred from *Tettigometra*.

**Nomenclatural status.** Name available and valid (Lindberg, 1948) [38]. Original description based on type material from Lindberg’s study.

**Illustrated.** Lindberg (1948, Figure 6C [38] (based on type material).

⁠

**Note.** Lindberg (1948) [38] distinguished *cerina* by its pale yellowish coloration and the “relatively short” (sic) digitiform mediodorsal aedeagal process. Its genital structures fall fully within the diagnostic range of *Mitricephalus* ***stat. rev.*** (digitiform, apically rounded mediodorsal process with parallel margins). By contrast, *Tettigometra cerina sec.* Mitjaev (1971: Figure 17) [64], with a broadly triangular mediodorsal aedeagal process, does not belong here but instead corresponds to the genus *Erratometra **gen. nov.***, as discussed above.

⁠

- ***Mitricephalus macrocephalus*** **(Fieber, 1865)**

Figure 7f

*Tettigometra macrocephala* Fieber, 1865 (original combination)

=*Tettigometra longiceps* Signoret, 1866 (syn. Fieber, 1872b: 2)

→*Mitricephalus macrocephalus* (Fieber, 1865) (present combination)

**Taxonomic status.** Valid species of *Mitricephalus* ***stat. rev*.** Type species of the genus *Mitricephalus* Signoret, 1866 (by subsequent designation of Baker, 1924) [36].

**Nomenclatural status.** Name available and valid (Fieber, 1865) [34]. Type material: syntypes from Fieber’s collection, preserved in MNHN.

⁠

**Illustrated.** Lindberg (1948) [38], Mitjaev (1971) [64], Logvinenko (1975) [66], Emeljanov (1980) [29], all based on non-type material.

**Identified specimens.** Bergevin, Dlabola, Noualhier–Lethierry, Perrier (MNHN); Fieber syntypes (MNHN), and Mozaffarian (HMIM, MHND).

**Dissected material.** Male genitalia examined from HMIM and MHND specimens.

⁠

**Note.** Fieber (1872b) [60] placed *T. longiceps* Signoret, 1866 as a junior synonym of *T. macrocephala* Fieber, 1865 and followed by subsequent catalogues.

⁠

- ***Mitricephalus varia*** **(Fieber, 1865) *comb. nov.***

*Tettigometra varia* Fieber, 1865 (original combination)

→*Mitricephalus varia* (Fieber, 1865) (present combination)

⁠

**Taxonomic status.** Valid species of *Mitricephalus* ***stat. rev.***, here transferred from *Tettigometra*.

**Nomenclatural status.** Name available and valid (Fieber, 1865) [34]. Type material designated by Fieber; a lectotype is preserved in MNHN (Puton collection).

⁠

**Illustrated.** Lindberg (1948) [38] and Mitjaev (1971) [64], both based on non-type material.

**Identified specimens.** Puton, paratype/lectotype from Fieber (MNHN) and Mozaffarian (HMIM).

**Dissected material.** Male genitalia examined in HMIM.

⁠

**Note.** Puton (1875) [102] placed *T. varia* in synonymy with *T. pallicornis* Signoret, 1866, itself regarded as a synonym of *T. vitellina* Fieber (1872b: 2) [60], now placed in the genus *Mimarada* ***stat. nov.*** within the tettigometrinan group. Later authors (e.g., Lindberg 1948 [38]; Mitjaev 1971 [64]), however, treated *varia* as distinct, illustrating male genitalia consistent with the present *Mitricephalus* ***stat. rev.*** generic concept (digitiform mediodorsal aedeagal process, subapical paired ventral anal processes).

⁠

- **Unidentified species (potentially new)**

*Tettigometra eremi* Lindberg, 1948 *sensu* Emeljanov, 1969 [116]; Mitjaev, 1971 [64]; Logvinenko, 1975 [66] (non *T. obliqua* var. *eremi* Lindberg, 1948).

⁠

**Taxonomic status.** Misapplied name, not conspecific with *Erratometra eremi* (Lindberg, 1948) ***stat. nov.*** The material referred to as *eremi* by Emeljanov (1969) [116], Mitjaev (1971) [64], and Logvinenko (1975) [66] instead represents a distinct, as-yet unnamed species, here placed within *Mitricephalus* ***stat. rev.***

**Nomenclatural status.** No available name; the use of *eremi* by these authors constitutes a misidentification. Formal description of this taxon as a new species is required.

⁠

**Illustrated.** Mitjaev (1971: 73, Figure 8. 12–14) [64], Logvinenko (1975: Figures 223–224. 1–4) [66]. Not Lindberg (1948: Figure 14.A2) [38], not Logvinenko (1975, Figure 222.7) [66].

⁠

**Note.** Male genitalia are characterized by a basally constricted, high mediodorsal aedeagal process with an apically rounded apex, combined with uniform pale coloration and an elongated vertex. Although not fully finger-like, being basally constricted, this condition matches the *Mitricephalus* concept as redefined here. However, as figurated, the very sketchy anal tube shows the paired vental process is a less subapical than species of these group usually show. If confirmed by more precise observation, this taxa might be transferred into the *Mediodentometra* genus.

⁠

**• Unidentified species**

***Tettigometra sordida* Fieber, 1865** *sec.* Mitjaev, 1971, Figure 18. 18–20 [64].

⁠

**Taxonomic status.** Misapplied name for a taxon not conspecific with true *Tettigometra sordida* Fieber, 1865, tranferred in this paper to *Erratometra* ***gen. nov.***

**Nomenclatural status.** No available name; the use of *sordida* by this author constitutes a misidentification.

⁠

**Illustrated.** Mitjaev, 1971, Figure 18. 18–20 [64], based on non-type material.

⁠

**Note.** The specimens figured by Mitjaev (1971) [64] under the name *T. sordida* display a strongly developed, digitiform mediodorsal aedeagal process, apically rounded and posteriorly directed. This condition is consistent with the *Mitricephalus* ***stat. rev.*** generic concept here adopted, in contrast to the true *T. sordida* Fieber, 1865, which belongs to *Erratometra* ***gen. nov.*** and is characterized by a triangular mediodorsal process. However, Mitjaev’s illustrations are relatively schematic, and the peculiar posterior orientation of the digitiform process may reflect an artifact of drawing rather than a genuine morphological trait.

⁠

**8. Genus *Stirometra* Emeljanov 1980 *stat. nov.***

⁠

**Type species:** *Tettigometra costulata* Fieber, 1865 by monotypy Emeljanvov, 1980: 55

⁠

**Taxonomic status.** *Stirometra* Emeljanov, 1980 is here elevated to full generic rank (***stat. nov.***), upgraded from subgenus status in Emeljanov (1980) [29]. It is retained as a valid genus of the apexometrinan group within Tettigometrini.

**Nomenclatural status.** Name available and valid (Emeljanov, 1980) [29]. Type species fixation by monotypy (ICZN Art. 68.3). Gender: Feminine.

⁠

**Diagnosis.** In addition to the general character states shared by members of the genus-group, these species are characterized by the absence of mediodorsal process. Emeljanov (1980) [29] listed for subgenus *Stirometra* the following distinguishing features in his key: aedeagus without lateral thickenings (Figure 3.1), in contrast to the condition in subgenus *Eurychila*; pronotum and mesonotum bearing distinct longitudinal carinae (Figure 1.2), which are absent in all other *Tettigometrini* species. The tegument is rugose and matte. In *Stirometra* ***stat. nov.***, the metatibia bears nine apical teeth, according to Emeljanov, 1980 [29] and may be distinctive from genus *Eurychila,* where only seven teeth were observed in true “decorata”. Versus genus *Eurychila **stat. rev.**,* the anal tube in *Stirometra* ***stat. nov.*** is shorter than high (Figure 6a), with a more developed sharp apical ventral process.

⁠

**Included species:** *Stirometra costulata* (Fieber, 1865), and two unidentified species: *Stirometra decorata* (Signoret, 1866) *sec.* Dlabola (MNHN) and *Stirometra brunnea* (Signoret, 1866) *sec.* Dlabola (MNHN),

⁠

**Geographical distribution:** Northern Africa [Algeria, Egypt, Morocco, Tunisia], Southern Europe [Italy, Portugal, Spain, Ukraine], Western Asia [Armenia, Azerbaijan, Cyprus, Israel, Jordan, Syria], Southern Asia [Afghanistan, Iran], Central Asia [Kazakhstan, Tadzhikistan, Turkmenistan, Uzbekistan], Southeastern Asia [Maritime] (Mozaffarian et al., 2018 [1]; Bourgoin, 2025 [2]; Fokker, 1900 [48]; Nast, 1972 [52]; Dlabola, 1957 [67]; Logvinenko, 1971 [66]; Mozaffarian & Wilson, 2011 [69]; Lindberg, 1963 [79]; Dlabola, 1965a [81]; Linnavuori, 1965 [95]).

⁠

**Specimens studied:**

- ***Stirometra costulata* (Fieber, 1865) *comb. nov.***

(Figure 7i)

*Tettigometra costulata* Fieber, 1865 (original combination)

=*Tettigometra heydeni* Kirschbaum, 1868 (syn. by Fieber 1872b: 2)

=*Tettigometra parviceps* Signoret, 1866 (syn. by Fieber 1872b: 2)

→*Tettigometra (Stirometra) costulata* (Fieber, 1865) (transferred by Emeljanov, 1980: 55)

→*Stirometra costulata* (Fieber, 1865) ***comb. nov.*** present paper.

**Taxonomic status.** Valid species of *Stirometra* Emeljanov, 1980, ***stat. nov.*** upgraded here to full genus rank from subgenus status. It is the type species of the genus by monotypy.

**Nomenclatural status.** Name available and valid (Fieber, 1865) [34]. Originally described in *Tettigometra*, later designated as type species of the subgenus *Stirometra* by Emeljanov (1980) [29]. Formally transferred here to *Stirometra* ***stat. nov.***

⁠

**Illustrated.** Lindberg (1948) [38], Mitjaev (1971) [64], Logvinenko (1975) [66], Emeljanov (1980) [29], all based on non-type material.

**Identified specimens.** Dlabola, Mozaffarian (HMIM); Dlabola, Puton (MNHN).

**Dissected material.** Male genitalia examined in HMIM.

⁠

**Note.** The species was regarded by Fieber (1872b) [60] as the senior synonym of *Tettigometra heydeni* Kirschbaum, 1868 and *T. parviceps* Signor aet, 1866, a treatment followed in subsequent catalogues. Metcalf (1932) [37] further listed three infraspecific varieties (*abrupata, albofasciata, unifasciata*) described by Fieber in 1872b [60].

⁠

**Problematic taxa, possibly new species,**

**identified as part of the genus *Stirometra stat. nov.*:**

- **Stirometra unidentified species no. 1 (potentially new)**

⁠

*Tettigometra brunnea* Signoret, 1866 *sec.* Dlabola (MNHN), *nec* Signoret, 1866

≠*Tettigometra brunnea* Signoret, 1866 *sensu* Puton coll. (♀, *incertae sedis*).

≠*Eurychila afra* (Kirschbaum, 1868) ***stat. nov.***, ***comb. nov.***

⁠

**Taxonomic status.** A distinct, probably undescribed species of the apexometrinan group, not conspecific with *T. brunnea* Signoret, 1866 or *Eurychila afra* (Kirshbaum, 1868).

**Nomenclatural status.** Misapplied name (*sec. Dlabola*). This interpretation does not correspond to Signoret’s original species concept of *brunnea*, nor to *T. afra* Kirshbaum, and has no nomenclatural validity.

⁠

**Identified and dissected specimens.** Material identified by Dlabola (MNHN); male genitalia examined from specimens from the Dlabola collection (MNHN).

⁠

**Note.** The specimen identified as *T. brunnea* by Dlabola differs markedly from *T. brunnea* se. Puton coll. and from *Eurychila afra* (Kirschbaum, 1868) in both habitus and male genital structure. Its placement within the apexometrinan group is clear and justifies its transfer to *Stirometra* as defined in the present revision.

⁠

- ***Stirometra* unidentified species no. 2 (potentially new)**

*Tettigometra decorata* Signoret, 1866 *sec.* Dlabola (MNHN), *nec* Signoret, 1866

→*Stirometra* ***stat. nov.*** (present transfer)

⁠

**Taxonomic status.** Probably a distinct, undescribed species, not conspecific with *T. decorata* Signoret, 1866.

**Nomenclatural status.** Misapplied name (*sec. Dlabola*). This interpretation does not correspond to Signoret’s 1866 original species concept [35] and has no nomenclatural validity.

⁠

**Identified specimens.** By Dlabola in MNHN’s collections.

**Dissected material.** Male genitalia examined from specimens in the Dlabola collection (MNHN).

⁠

**Note.** Habitus and male genitalia of the examined specimens in the Dlabola collection in MNHN differ from those of *Eurychila decorata* as illustrated by Lindberg (1948) [38] and Bourgoin (1988b) [43], and from specimens in other collections. True *Eurychila decorata* (Signoret, 1866) is characterized by a swollen dorsal aedeagal margin and seven metatibial apical teeth, character states not found in the Dlabola specimens (swelling absence, nine apical metatibial teeth). These specimens identified by Dlabola might represent an undescribed species within the *Stirometra* genus concept.

⁠

**IV. Species *inquirendae* or in *incertae sedis* position**

⁠

Fourteen nominal species historically assigned to *Tettigometra* cannot be confidently placed in *Tettigometra* sensu stricto or in any of the newly defined generic groups proposed in this work. This is primarily due to incomplete or inadequate original descriptions, lack of access to male specimens, or the fact that some taxa were described from females only, whose morphological features are insufficient for reliable generic placement. Seven of these taxa, not recollected since their original descriptions and very poorly documented, are here regarded as *species inquirendae* of doubtful taxonomic status. The remaining six are treated as valid Tettigometrini species but are retained *incertae sedis*, as their generic placement cannot be resolved with the available evidence.

⁠

***Tettigometra atrovirens*** **O. Costa, 1834**

⁠

**Taxonomic status.** *Species inquirenda*.

**Nomenclatural status.** Valid name (Costa, 1834) [129]. Consistently cited in faunal lists in Fieber (1876) [130], Servadei (1967) [131], and Nast (1972) [52]. No synonymies or transfers proposed.

⁠

**Note.** Originally described by Costa (1834: 83) [129] from Italy. The species has not been re-collected since its original description, and its identity remains doubtful. Without type re-examination, the taxon is unassignable and is here retained as a *species inquirenda*.

⁠

***Tettigometra barbata*** **Mitjaev, 1971**

⁠

**Taxonomic status.** *Tettigometra incertae sedis*

**Nomenclatural status.** Valid name (Mitjaev, 1971) [64]. Transferred between genera by later authors: *Brachyceps* (Nast, 1972) [52] and *Tettigometra* (*Tettigometra*) (Emeljanov, 1980) [29]. No synonymies proposed.

⁠

**Note.** The species was originally described and illustrated as *Brachycephalus barbatus* Mitjaev (1971: 75) [64] in his identification key to the planthoppers of Kazakhstan, based on both male and female specimens. It was later listed by Nast (1972) [52] in *Brachyceps* and by Emeljanov (1980) [29] in the subgenus *Tettigometra*. The species was not available for examination in the present study. Mitjaev’s figures (1971: Figures 18, 30 and 31) [64] and Emeljanov’s key (1980) [29] indicate a convex frons, a feature consistent with the herein genus concept of *Brachyceps* ***stat.rev.*** and *Metroplaca*, ***stat. nov.***, but not with *Metroplaca* ***stat. nov.***, due to the absence of shiny, broad-margined forewings, nor with *Mimarada* ***stat. nov.***, which, according to Emeljanov’s definition, has a wrinkled and matte integument. Male genitalia were neither described by Mitjaev (1971) [64] nor treated in later works. Based on the external characters illustrated by Mitjaev (1971) [64] and Emeljanov’s (1980) [29] intrinsc definition from its key of the subgenus *Tettigometra*, the species could potentially belong to *Brachyceps, Mediodentomerta or Tettigometra s.l*., pending future re-examination of the male genitalia and type material.

⁠

***Tettigometra brachynota*** **Fieber, 1865**

⁠

**Taxonomic status.** *Tettigometra incertae sedis*.

**Nomenclatural status.** Valid name (Fieber, 1865). Subsequently listed by Puton (1875), Fieber (1876), Puton (1886), Melichar (1896), Lindberg (1948), and Nast (1972). Holzinger (2009) mentioned it as a probable synonym of *T. virescens*, but without citing a source; such a synonymy is therefore nomenclaturally unjustified (ICZN Art. 61.2).

⁠

**Note.** Originally described by Fieber (1865: 565) [34]. No specimens or data on male genitalia were available in this study. Pending re-examination of type material, the identity of the species cannot be clarified, and it is provisionally retained as *incertae sedis*.

⁠

***Tettigometra brunnea*** **Signoret 1866**

(Figure 8a)

⁠

**Taxonomic status.** *Tettigometra incertae sedis.*

**Nomenclatural status.** Valid name (Signoret, 1866: 157) [35]. Subsequently cited as a valid species by Puton (1899) [105], Oshanin (1907) [132], Horváth (1912) [106], Metcalf (1932) [37], Servadei (1967) [130], and Nast (1972) [52]. Earlier synonymies with *T. picea* Kirschbaum, 1865 and *T. afra* Kirschbaum, 1868, proposed by Puton (1899) [105] and Horváth (1912) [106] respectively, are rejected herein.

⁠

**Note.** In this study, the specimen labeled “*T. brunnea = picea*” from the Puton collection (MNHN, Paris) was re-examined. It consists of a female, which differs from *Eurychila afra* (Kirschbaum, 1868) and *T. picea* Kirschbaum, 1865 by its paler coloration, shorter tegmina with a weaker costal groove, shinier pronotum, and a pale whitish basal band on the frons. However, as no male specimens are available, its diagnostic features cannot be confirmed. The lack of male genital information and the uncertain correspondence with Signoret’s original concept of *brunnea* preclude confident placement within the revised generic framework. The species is therefore provisionally retained as *incertae sedis* within *Tettigometra s.l*.

⁠

***Tettigometra deltoton*** **Fennah, 1958**

Figure 8b

⁠

**Taxonomic status.** Treated here as *species inquirenda*, possibly a junior synonym of a European species.

**Nomenclatural status.** Valid name (Fennah, 1958) [133]. Not subsequently treated in detail in the literature, and no synonymies or transfers have been proposed.

⁠

**Note.** *T. deltoton* was described by Fennah (1958) [133] from South Africa, based only on female specimens. According to his account, the species most closely resembles *T. lepida* Fieber (=*Brachyceps laeta* (Herrich-Schäffer, 1835) ***comb. nov.***, but differs by its more tumid clypeus and tegminal coloration, and from *T. mongolica* Lindberg (= *Tettigometra burjata* Kusnezov, 1929) by the punctation of the head and thorax (smooth in *burjata*). It further differs from *Micracanthometra impressifrons* (Mulsant & Rey, 1855) ***comb. nov.*** by the frons, shallowly convex across the disc (indented in *impressifrons*), and from *T. atra* Hagenbach by its distinctly more transverse head. Fennah also emphasized that no *Tettigometra* had previously been recorded south of the Sahara and that *T. deltoton* was unlike any then-known African species, justifying description from female material alone.

⁠

However, the absence of male material and the weak diagnostic separation leave the identity of this species highly doubtful. Its morphology, as summarized by Fennah, overlaps with European taxa, raising the possibility that *T. deltoton* may represent an introduced species transported with cultivated plants to South Africa. Pending discovery of male specimens and re-examination of the type material, the species is here treated as a *species inquirenda* and may eventually prove to be a junior synonym of an already described European taxon.

⁠

***Tettigometra demavenda*** **Dlabola, 1981**

(Figure 8c)

⁠

**Taxonomic status.** *Species inquirenda*.

**Nomenclatural status.** Valid name (Dlabola, 1981) [83]. No subsequent combinations or synonymies have been proposed.

⁠

**Note.** Described from northern Iran by Dlabola (1981: 170) [83] and said to resemble *T. pseudovitellina*, differing slightly in vertex conformation. Male genitalia were neither described nor illustrated in the original publication. In the present study, one type specimen borrowed from the Prague museum was examined, but in the absence of diagnostic male genitalia characters, the taxon could not be placed further. Since its description, *T. demavenda* has never been recorded again and the taxon remains doubtful; it is therefore here regarded as a *species inquirenda*.

⁠

***Tettigometra diminuta*** **Matsumura, 1910**

⁠

**Taxonomic status.** *Species inquirenda* (probable synonym of *T. baranii* Signoret, 1866).

**Nomenclatural status.** Valid name (Matsumura, 1910) [134]. Later mentioned by Lindberg (1948) [38] and Nast (1972) [52], who transferred it to *Micrometrina*. Although Lindberg (1948) [38] listed *T. diminuta* and *T. baranii* as distinct species (nos. 34 and 35), he noted that he was unable to separate them in his key (couplet 25), however without formal synonymy. Accordingly, Holzinger et al. (2003) [28] placed it in synonymy with *T. baranii.* Although Holzinger mentioned that, in his view, he did not formally synonymize the species (pers. com.), the synonymy attributed by Holzinger et al. (2003) to Lindberg [38] should therefore be credited to Holzinger et al. (2003) [28] under ICZN authorship conventions.

⁠

**Note.** The species was not available for examination in this study. Given the conflicting interpretations in the literature and the absence of a re-examination of type material, *T. diminuta* is here provisionally retained as a distinct *species inquirenda*, pending confirmation of its synonymy with *T. baranii*.

⁠

**Figure 8:**
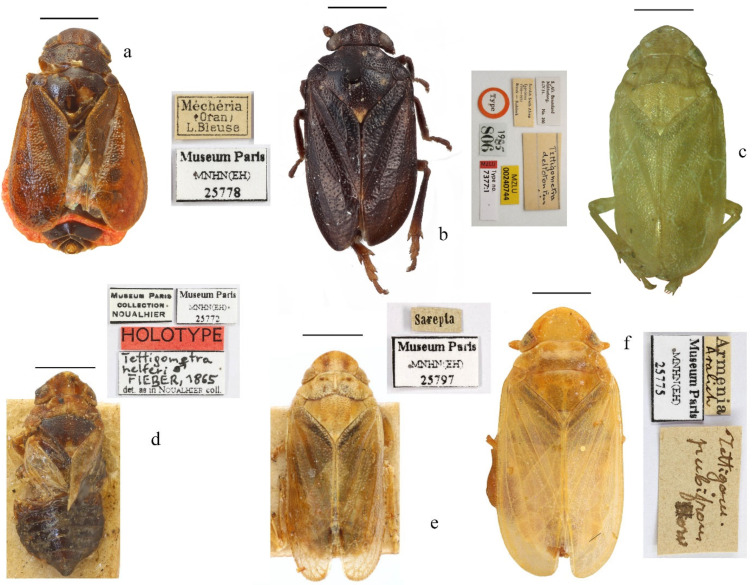
Habitus of four species in *Tettigometra s.l.* (**a**) *T. brunnea* Signoret, 1866 (Muséum national d’Histoire naturelle, Puton collection, photo by Laurent Fauvre), (**b**) *T. deltoton* Fennah, 1958 (type specimen in Biological Museum in Lund University, Sweden, photo by Rune Bygebjerg, (**c**) *T. demavenda* Dlabola, 1980 (type specimen in National Museum, Prague, Czech Republic, adapted from Mozaffarian et al., 2018), (**d**) *Tettigometra helferi* Fieber, 1865 (Holotype, Muséum national d’Histoire naturelle, photo by Laurent Fauvre) (**e**) *T. peliotaena* Fieber, 1865 (Muséum national d’Histoire naturelle, Puton collection, photo by Laurent Fauvre), (**f**) *T. publifrons* Horváth, 1894 (Muséum national d’Histoire naturelle, Puton collection, photo by Laurent Fauvre). Scale: 1 mm.

⁠

***Tettigometra exigua*** **(Horváth, 1901)**

⁠

**Taxonomic status.** *Tettigometra incertae sedis*.

**Nomenclatural status.** Valid name (Horváth, 1901) [135]. Later mentioned as a valid species by Lindberg (1948) [38] and Nast (1972) [52], the latter using the combination *Brachyceps exiguus* (Horváth, 1901). No synonymies have been proposed.

⁠

**Note.** Since its original description (Horváth, 1901) [135], the species has not been redescribed or revised. No specimens nor data on male genitalia were available in this study, making a reliable generic placement impossible. Although transferred to *Brachyceps* by Nast (1972) [52], the taxon is here provisionally retained as incertae sedis within *Tettigometra* until further evidence becomes available.

⁠

***Tettigometra helferi* Fieber, 1865**

(Figure 8d)

⁠

**Taxonomic status.** *Tettigometra incertae sedis.*

**Nomenclatural status.** Valid name (Fieber, 1865: 566) [34]. Subsequently cited by Puton (1875 [102], 1886 [44]), Fieber (1876) [130], Lindberg (1948) [38], and Nast (1972) [52] as a valid species. Considered a synonym of *Tettigometra impressifrons* Signoret, 1866 by Oshanin (1912: 124) [136], a treatment followed by Metcalf (1932: 29) [37] as “equals *T. impressifrons* Sign. [in pars]”. However, this synonymy is unsubstantiated and may have been intended only as a partial synonymy, since *helferi* and *impressifrons* were described independently by Fieber (1865) [34] and Signoret (1866) [35], respectively.

⁠

**Note.** Male genitalia of *T. helferi* were not examined in this study, and no illustrations are available in the literature. A photo of the female holotype is provided in Figure 8d. In the absence of diagnostic male characters, the taxon cannot be assigned with confidence to any of the revised genera. Given the unresolved synonymy issue with *T. impressifrons* and the lack of modern revision of type material, *T. helferi* is provisionally retained here as *incertae sedis* within *Tettigometra*

⁠

***Tettigometra nasikornis*** **Mitjaev, 1971**

⁠

**Taxonomic status.** *Species inquirenda* (taxon of doubtful identity, possibly not a valid species).

**Nomenclatural status.** Valid name (Mitjaev, 1971: 75) [64], described from Kazakhstan. Later transferred to subgenus *Tettigometra* by Emeljanov (1980) [29]. No synonymies proposed.

⁠

**Note.** Originally described only in an identification key (Mitjaev, 1971) [64] without reference to male genitalia, and illustrated merely by forebody features (Figures 25 and 26; p. 18). The main diagnostic character cited was a convex frons with a median spine. However, such a structure is typical of immatures of Tettigometridae, where a frontal spine represents an apomorphic character that normally disappears in adults. We are not aware of any new collects of the species since its description. This suggests that the condition described by Mitjaev may reflect a teratological or neotenic trait rather than a valid species character. Pending re-examination of type material and especially of male genitalia, *T. nasikornis* is here regarded as a *species inquirenda*.

⁠

***Tettigometra pallipes*** **(Lucas, 1853)**

⁠

**Taxonomic status.** *Species inquirenda.*

**Nomenclatural status.** Originally described as *Issus pallipes* Lucas, 1853 (Issidae) from Greece by Lucas (1853) [137]. Later transferred to *Tettigometra* by Gnezdilov (2017) [138], who demonstrated that the original illustration actually depicts a fifth-instar larva of Tettigometridae. No synonymies have been formally proposed.

⁠

**Note.** The species is based solely on a larval stage, making its diagnostic features unreliable for defining a valid adult taxon. No information on male genitalia is available, and the type material cannot provide the characters necessary for generic placement. Consequently, the identity of *T. pallipes* cannot be verified, and the species is here regarded as a *species inquirenda.*

⁠

***Tettigometra peliotaenia*** **Fieber, 1865**

(Figure 8e)

⁠

**Taxonomic status.** *Tettigometra* incertae sedis.

**Nomenclatural status.** Valid name (Fieber, 1865: 565) [34]. Subsequently cited as a valid species by Fieber (1876) [129], Puton (1886) [44], Melichar (1896) [139], Lindberg (1948) [38], Servadei (1967) [131], and Nast (1972) [52]. No synonymies have been proposed.

⁠

**Note.** In this study, several female specimens identified by Puton and Bergevin were examined in the MNHN collections, but no males were available. The lack of diagnostic information on male genitalia prevents reliable placement within the revised generic framework. Consequently, the species is provisionally retained as *incertae sedis* within Tettigometrini.

⁠

***Tettigometra pistacina*** **O. Costa, 1834**

⁠

**Taxonomic status.** *Species inquirenda*.

**Nomenclatural status.** Valid name, originally described as *pistacina* by Costa (1834: 83) [129]. The spelling *pistacina* must be retained as the correct original spelling under ICZN Article 32.2, since there is no evidence in the original description that it was a lapsus or inadvertent error. The alternative spelling *psittacina*, consistently used in subsequent literature (e.g., Fieber, 1872b [60], 1876 [129]; Puton, 1875 [102], 1886 [44], 1899 [105]; Oshanin, 1907 [131], 1912 [136]; Lindberg, 1948 [38]; Servadei, 1967 [131]; Nast, 1972 [52]), constitutes an unjustified emendation (ICZN Arts. 33.2, 33.3) and must be regarded as an incorrect subsequent spelling (ICZN Art. 33.3.1).

⁠

**Note.** The species has not been recollected since its original description, and its identity remains uncertain. Pending rediscovery or re-examination of the type material, *T. pistacina* is here regarded as a *species inquirenda* within Tettigometrini incertae sedis.

⁠

***Tettigometra pubifrons*** **Horváth, 1894**

(Figure 8f)

⁠

**Taxonomic status.** *Tettigometra incertae sedis.*

**Nomenclatural status.** Valid name (Horváth, 1894: 184) [140]. Subsequently cited as a valid species by Lindberg (1948) [38] and Nast (1972) [52]. No synonymies have been proposed.

⁠

**Note.** In this study, one female specimen identified by Puton was examined in the MNHN collections. However, no male specimens were available and no information on male genitalia has ever been published. In the absence of these diagnostic characters, the species cannot be reliably assigned to any of the revised genera and is provisionally retained as *incertae sedis* within Tettigometrini.

⁠

## 4. Discussion

### 4.1. Strategy and Methodology

- **Limitations of the current suprageneric classifications**

Despite long-standing efforts to structure the suprageneric classification of *Tettigometra*, each historical system has presented significant limitations. Signoret’s (1866) [35] initial subdivision into subgenera was based in part on erroneous observations of antennal segmentation. Lindberg’s (1948) [38] influential key, although pioneering in scope, often blurred the distinction between descriptive and diagnostic traits, focusing more on species-level identification than on supraspecific classification. As a result, it offered limited utility for group recognition or comparative analysis across species. Later, Mitjaev’s (1971) [64] treatment introduced inconsistencies in the identification of several described species, leading to parallel and sometimes contradictory paths of interpretation.

In an attempt to bring greater coherence, Emeljanov (1980) [29] proposed a revised supraspecific system based primarily on male genitalia (mainly the aedeagus), however supplemented by a very limited number of external morphological traits. He acknowledged that no clear diagnostic features could reliably distinguish *Tettigometra* from previously described genera (e.g., *Mitricephalus*, *Eurychila*, *Macrometrina*, *Micrometrina*, *Brachyceps*) and therefore synonymized them under *Tettigometra*, subdivided into eight subgenera, including several new ones.

However, in most of these groupings—six out of the eight currently recognized subgenera—the definitions rely on narrowly defined and highly specific characters, often applicable to only one or two species, or on subtle morphological tendencies that are difficult to delimit in a consistent and operational way. In addition, many species were left unassigned, and the identification key was published without accompanying descriptions or formal diagnoses, requiring users to infer defining features solely from the key.

Combined with the very limited number of other morphological characters, often vague, weakly diagnostic, or prone to intraspecific variation, Emeljanov’s 1980 [29] minimalist taxonomic approach raised concerns not only about the practical usability of its classification but also about its taxonomic validity and conceptual soundness. The result is a current suprageneric framework with limited predictive value, which neither enables consistent identification nor facilitates the integration of newly described species, particularly from undersampled or poorly known regions.

- **Taxonomic strategy and rationale for new generic delimitation**

**Identifying robust morphological groups.** To overcome these limitations, we adopted a strategy aimed at identifying consistent, morphologically coherent groups that would better reflect potential natural lineages and provide a more operational classification. Among the 50 morphological descriptors analyzed in our study, the male terminalia proved to be the most informative. In particular, the position of the ventral process of the anal tube emerged as the single most diagnostic feature, allowing for a primary division of *Tettigometra* s. l. species into two main coherent units: the tettigometrinan and apexometrinan groups. Although this character had already been noted by Lindberg (1948) [38], it had previously been applied only to a limited subset of species.

**Criteria for subgroup delimitation.** Within these two main groups, the presence or absence, and the shape, of the mediodorsal aedeagal process in lateral view served as a secondary criterion for defining several subgroups. This character, along with tegminal shape, had also been used by Emeljanov (1980) [29] to distinguish among his subgenera. The subgrouping proposed here is further supported by additional morphological traits, including the overall shape of the anal tube, the structure of the periandrium, and other features of the male terminalia, as well as tegminal morphology, the latter first emphasized by Signoret (1866) [35] and later refined by Emeljanov (1980) [29]. Together, these characters increase the likelihood that the groups defined in this study reflect natural (i.e., phylogenetically coherent) lineages.

This raises the question of whether the newly defined subgroups should be formally recognized as subgenera, as genera, or even whether the two larger clusters could merit recognition at the subtribal level.

Following the precedent set by Baker (1924) [36] and Metcalf (1932) [37], we chose to formally recognize the subgroups at the generic rank, based on the following considerations: (1) none of the groupings contradict previously described subgenera or morphotypes (e.g., Signoret, Fieber, Emeljanov); (2) each group displays strong internal morphological coherence, particularly in the structure of male genitalia; (3) their circumscription can be consistently and meaningfully extended to include additional described species; and (4) their geographic distributions show clear biogeographic consistency. In practical terms, these groups also (5) offer an operationally useful framework for species identification—especially when female characters are uninformative, and particularly in species with wide or altitudinally variable ranges where cryptic diversity has been suspected (Mozaffarian & Bourgoin, 2019) [39]. Moreover, (6) this new generic-level classification framework enhances nomenclatural stability for historically ambiguous species complexes, and (7) provides a robust and scalable foundation for future integrative taxonomic studies incorporating molecular, ecological, and biogeographic data.

Avoiding premature suprageneric classification: Nonetheless, we refrained from formally establishing new subtribes for the two broader genus groups identified, following a conservative strategy, similar to that recently adopted in Cixiidae and Flatidae (Luo et al., 2025 [141]; Ai et al., [142]). However, in contrast to the Cixiidae and Flatidae, where such informal groups are already supported as monophyletic and treated as “lineages” of unclear relationships, their monophyly within Tettigometridae remains unconfirmed. Consequently, we provisionally refer to these larger assemblages as the tettigometrinan and apexometrinan “taxonomic groups” contra “phylogenetic lineages”.

Indeed, based on the two alternative character states, the median versus apical position of the paired ventral anal tube processes (likely linked to whether the posterior and ventral margins of the anal tube are in smooth prolongation or form an angle), we provisionally regard the atypical submedian position characterizing the tettigometrinan group as the apomorphic condition. This, in turn, suggests that the apexometrinan group may be paraphyletic, representing a grade rather than a clade. For these reasons, and to avoid falling into the “autapomorphy trap” (Bourgoin & Campbell, 2002) [143], we conservatively retain both assemblages as informal “taxonomic groups,” refraining for the moment from assigning them formal taxonomic rank (which would require monophyly) or recognizing them as phylogenetic clades (which requires evidence of common ancestry), pending further corroboration, particularly from molecular phylogenetic analyses.

**A conservative and operational approach at the generic and specific levels.** Similarly, and in the interest of taxonomic stability and operational clarity, we deliberately refrained from proposing premature synonymies among previously established genera and species, with one exception for *Macrometrina* synomized with *Mitricephalus*, for which we could not find any differencing valid characters. In reverse, we underlined several new potential species but without formal description by a valid name, due to lack of specimens, only suggested by figures. Future molecular approaches will be necessary to resolve these taxonomic dilemmas with greater confidence.

At this stage of our revision, our objective was not to resolve all issues of genus- or species-level identification or nomenclatural priority, but rather to establish a coherent and morphologically consistent framework of taxonomic groups within Tettigometrini. In several cases we encountered divergent species concepts in the literature and the examined collections, and we acknowledge that it is currently not possible to determine with certainty which concept corresponds most closely to the original description. Rather than accepting old unsubstantiated synonymies for which doubts have been expressed, or introducing premature new synonymies or name applications, or describing new species, we have chosen to retain these names as secundum usages, pending a thorough examination of type material where possible, additional morphological analyses if available, and necessary new molecular evidence. We anticipate that future research, particularly integrating phylogenetic analyses and type revisions, will refine the species-level taxonomy within the framework proposed here.

**A pragmatic framework for future work.** This pragmatic approach preserves nomenclatural stability, avoids premature taxonomic inflation, and ensures operational clarity in the current state of knowledge. At the same time, it offers a flexible and coherent framework that can accommodate future phylogenetic refinements. The classification proposed here thus provides a solid and realistic foundation for the continued revision and study of *Tettigometrini.*

### 4.2. Taxonomic Results

- **A new generic framework for *Tettigometra s.l.***

**Figure 9:**
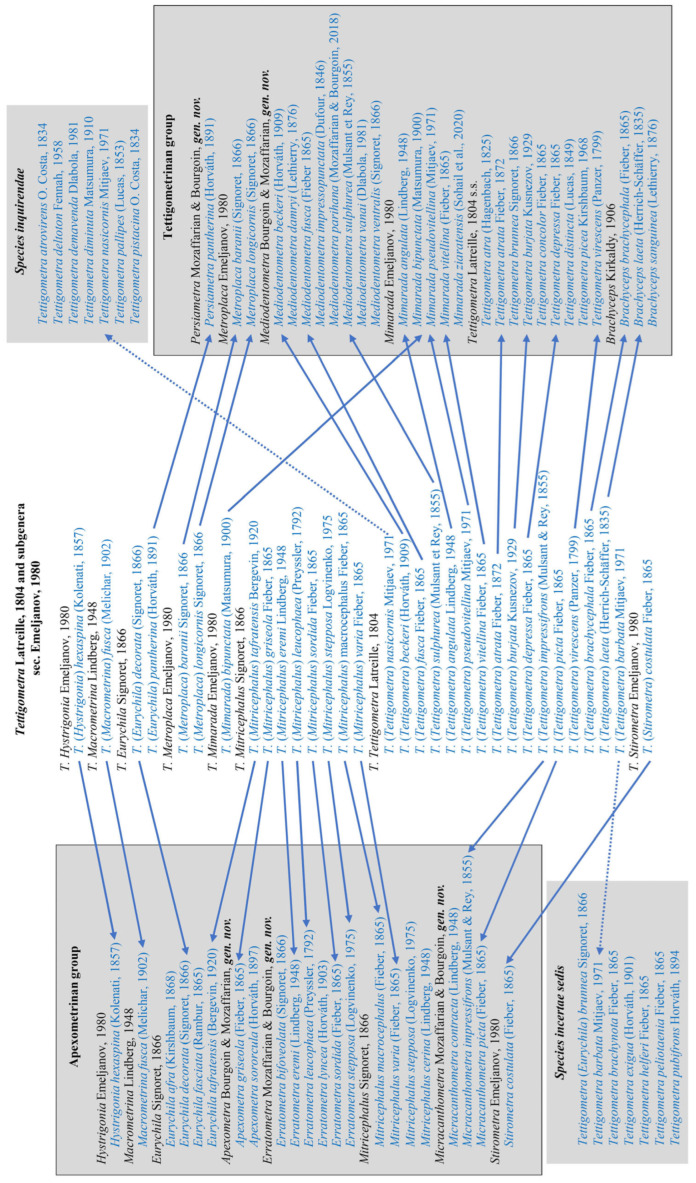
Summary of the new generic framework proposed for Tettigometrini, dividing *Tettigometra s.l.* and 8 subgenera *sec.* Emeljanov (1980) [29] into 14 new distinct genera.

The new classification of the Tettigometrini, which for a long time included only the broadly defined genus *Tettigometra*, is here revised to comprise 14 genera, arranged into two well-defined groups: tettigometrinan and apexometrinan. This framework refines the previous classification of Emeljanov (1980) [29], who recognized a single genus with eight subgenera (Figure 9). Following the methodological approach and taxonomic strategy outlined above, and in particular our choice to avoid premature synonymy in the absence of stronger molecular evidence, all of these subgenera are here elevated to full generic rank and no synonymy of previous genera is proposed. However, we underlined the case of the monospecific genera that might be based on characters of specific value only and particularly the *Macrometrina/Mitricephalus* issue. In addition, five new genera are proposed, each defined by a clear set of diagnostic character states.

The monotypic genera *Hystrigonia **stat. nov.***, *Macrometrina **stat. nov.***, and *Stirometra **stat. nov.*** remain unchanged from Emeljanov’s treatment, just upgraded to genus level. The species composition of *Metroplaca **stat. nov.*** is also retained as originally proposed. *Mimarada **stat. nov.***, however, is expanded to include four species previously placed in the subgenus *Tettigometra sensu* Emeljanov, together with a recently described species, *Mimarada ziaratiensis* (Sohail et al., 2020). In contrast, *Mitricephalus sensu* Emeljanov proves to be a polyphyletic taxonomic concept within the apexometrinan group, with its constituent species now redistributed among *Apexometra **gen. nov.***, *Erratometra **gen. nov.***, *Eurychila **stat. rev.***, and *Mitricephalus* ***stat. rev.***

Although most of the genera recognized by Emeljanov were consistent with the two broader groupings retained here, two subgeneric concepts of Emeljanov *Eurychila* and *Tettigometra* require particular correction and are no longer tenable. (1) *Eurychila* mixed apexometrinan and tettigometrinan species; the latter is segregated here as the monotypic *Persiametra* ***gen. nov.*** (*P. pantherina* ***comb. nov.***), while *Eurychila* itself now contains *E. decorata* plus *E. fasciata* ***comb. nov.*** and *E. tafratensis* ***comb. nov.***, forming a consistent apexometrinan unit. (2) The subgenus *Tettigometra sensu* Emeljanov also combined taxa from both groups. All apexometrinan elements are here transferred to the new genus *Micracanthometra* ***gen. nov.*** (which also includes *M. contracta* ***comb. nov.***, a species not considered by Emeljanov), while the remaining tettigometrinan species are redistributed among other genera of that group, revealing the polyphyly of Emeljanov’s concept of *Tettigometra* (*Tettigometra*).

**Figure 10:**
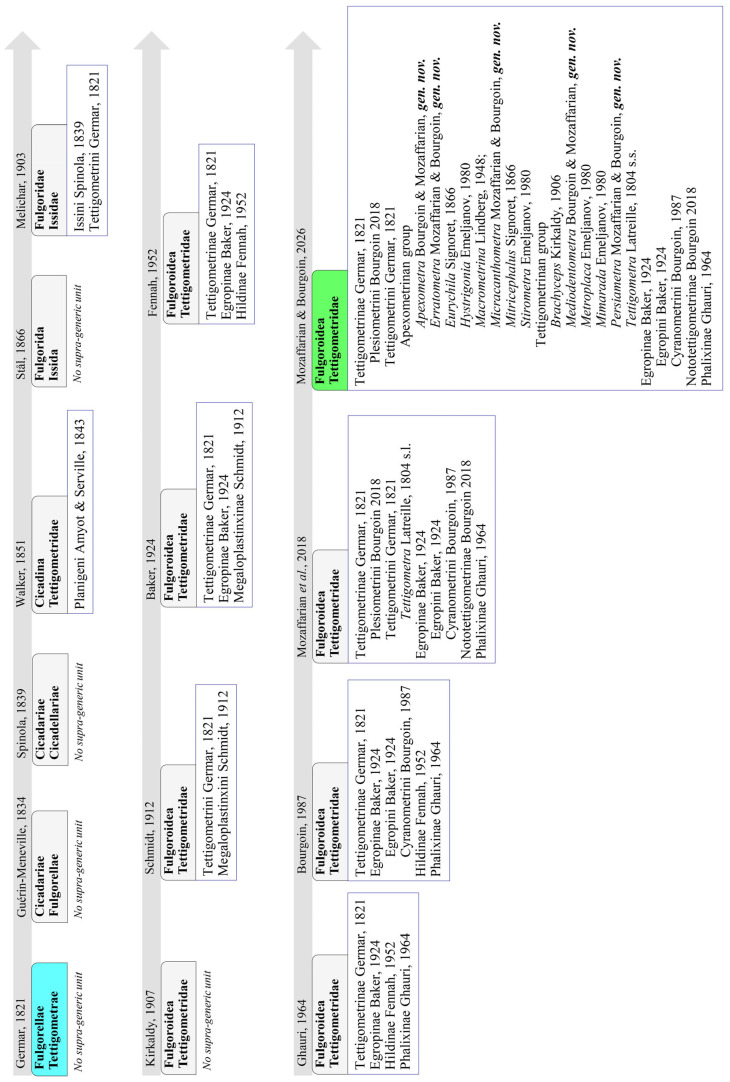
Evolution of the suprageneric classification of the family Tettigometridae from Germar (1821) to the present study (updated from Mozaffarian et al. (2018) [1] by integrating all genera of the tribe Tettigometrini, newly arranged into two informal groups (Apexometrinan and Tettigometrinan).

Figure 10 summarizes the new proposed supra generic classification of Tettigometridae, updated from Mozaffrian et al. (2018) [1] with all tettigometrini genera newly listed.

- **Recurrent identification conflicts and the limits of current diagnostic characters**

Although the new generic framework proposed here provides a more stable and operational classification, many problems remain unresolved at the species level. These issues are not restricted to simple misidentifications but often stem from deeper inconsistencies in how authors have historically conceived species boundaries. Several recurrent types of problems can be identified

1. Conflicting taxonomic species concepts: species names have often been applied with different meanings by different authors, resulting in conflicting taxonomic concepts.
  - *Mediodentometra beckeri* (Horváth, 1909) ***comb. nov.***: interpreted correctly by Lindberg (1948) [38], but misapplied as *T. beckeri sec.* Mitjaev (1971) to a taxon actually belonging to *Mimarada **stat. nov.***
  - *Erratometra pantherina* (Horváth, 1891) ***comb. nov.***: original concept of Horváth is clear, but Lindberg (1948) figured a different taxon under *T. pantherina*, corresponding instead to *Erratometra **gen. nov.***
  - *Mediodentometra fusca* (Fieber, 1865) ***comb. nov.***: three competing usages exist: Fieber’s original species; Mitjaev’s (1971) *T. fusca* representing a *Mimarada* sp.; and Dlabola’s interpretation closer to *Mediodentometra*.

2. Composite or mixed species concepts: some names have been used to cover morphologically heterogeneous assemblages, effectively mixing more than one taxon.
  - *Mimarada vitellina* (Fieber, 1865) ***comb. nov.***, *M. pseudovitellina* (Mitjaev, 1971) ***comb. nov.***, and *M. ziaratensis* (Sohail et al., 2020) ***comb. nov.***: a problematic trio with overlapping or poorly defined boundaries.
  - *Metroplaca baranii* (Signoret, 1866) ***comb. nov.*** and *M. diminuta* (Matsumura, 1910) ***comb. nov.***: treated as separate taxa by Lindberg (1948) [38] and Mitjaev (1971) [64], but synonymized by Holzinger et al. (2003) [28]; the historical material clearly represents heterogeneous concepts.

3. Hidden diversity under existing names: undescribed species have often been misfiled under available names.
  - *Mimarada* sp.: dissected specimens identified as *T. cerina sec.* Dlabola correspond to an undescribed taxon, distinct from *Mimarada cerina* (Lindberg, 1948) ***comb. nov.***
  - *Mimarada* sp.: the taxon figured as *T. fusca sec.* Mitjaev (1971) does not correspond to *Mediodentometra fusca* (Fieber, 1865) ***comb. nov.***, but to a separate undescribed *Mimarada* species.

4. Names based on inadequate or uninformative types: several names are anchored to types that are either female-only or insufficiently described, making them difficult to interpret.
  - *Mimarada akramowskajae* (Lindberg, 1960) ***comb. nov.***: described from only two specimens (1♂. 1♀). limiting its diagnostic value and interpretation
  - *Mimarada cerina* (Lindberg, 1948) ***comb. nov.***: originally described from female type material only; later inconsistently applied by Dlabola to dissected males actually belonging to another taxon.

5. Ambiguous synonymies: some synonymies were introduced without critical examination of type material, creating lasting confusion.
  - *Brachyceps brachycephala* (Fieber, 1865) ***comb. nov.***: synonymy involving *T. tumidifrons* Kirschbaum, 1868 and *T. lucida* Signoret, 1866 is accepted, but the historical basis of these synonymies remains uncertain.
  - *Mimarada bipunctata* (Matsumura, 1900) ***comb. nov.***: synonymized with *T. shikokuana* Ishihara, 1954 by Emeljanov (1977) [74], although some morphological differences suggest the latter may represent a distinct taxon.
  - *Metroplaca baranii* (Signoret, 1866) ***comb. nov.***: synonymized with *M. diminuta* (Matsumura, 1910) ***comb. nov.*** by Holzinger et al. (2003) [28], though earlier authors had treated them separately.

These categories of problems highlight that the instability at the species level is not merely due to careless identifications but also a structural issue. They reflect a long history of shifting species concepts, uneven attention to diagnostic characters and a choice of them which remains in any case poorly informative and focused on characters not sufficiently decisive, and the lack of systematic revision based on type material due to too-old, lost or not accessible, or limited to non-diagnostic useless females.

Female genital structures in Tettigometridae are remarkably uniform and provide no useful diagnostic variation at the generic level. External characters (color, size, wing shape) show continuous, overlapping variation and cannot reliably separate genera. Likewise, ant-association does not correlate with generic limits, as species of the same genus may occur with or without ants. These factors reinforce that only male genital morphology currently offers stable, reproducible characters for defining genera in Tettigometrini.

- ***Inquirenda*** **and *incertae sedis* species and the need for further investigation**

Fourteen nominal species historically assigned to *Tettigometra* could not be placed in *Tettigometra* sensu stricto or in any of the newly defined genera proposed in this work. Among them, seven nominal species (i.e., formally described species-level taxonomic concepts) (*T. barbata, T. brunnea, T. brachynota, T. exigua, T. helferi, T. peliotaenia,* and *T. pubifrons*) are here retained as Tettigometrini *incertae sedis*, i.e., valid species-level taxa whose generic placement cannot presently be resolved. In other words, their original species concepts are considered available, distinct, and biologically valid, but their assignment to a genus is not possible in the absence of adequate diagnostic male characters.

In contrast, seven nominal species (*T. atrovirens, T. deltoton, T. demavenda, T. diminuta, T. nasikornis, T. pallipes, and T. pistacina*) are treated as *species inquirendae.* These represent formally established species-level taxonomic concepts, but their identity cannot be reliably assessed based on the available evidence. However, while these taxonomic concepts are nomenclaturally available and were formally described, the correspondence of their original definitions to a distinct biological species entity remains uncertain or problematic.

To conclude, and given the well-known scarcity of reliable morphological characters in Tettigometrini and the necessary reliance of this revision on male terminalia, further taxonomic work, including re-examination of type specimens, collection of new male material, and the necessary integration of molecular data such as DNA barcoding, will be essential to clarify their status and generic placement. It should be stressed that several Tettigometrini species are based exclusively on external habitus characters alone, or female or even immature material, which provides insufficient diagnostic features in Tettigometrini. Additional junior synonyms or new cryptic species are also likely to emerge in the future from among these doubtful taxa once comparative analyses could be extended.

## Data Availability

The original contributions presented in this study are included in the article. Further inquiries can be directed to the corresponding author(s).
